# Tailoring Thermomechanical, Shape Memory and Self-Healing Properties of Furan-Based Polyketone via Diels-Alder Chemistry with Different Bismaleimide Crosslinkers

**DOI:** 10.3390/polym17050565

**Published:** 2025-02-20

**Authors:** Esteban Araya-Hermosilla, Marco Carlotti, Felipe Orozco, Guilherme Macedo R. Lima, Rodrigo Araya-Hermosilla, Daniela E. Ortega, Diego Cortés-Arriagada, Francesco Picchioni, Ranjita K. Bose, Virgilio Mattoli, Andrea Pucci

**Affiliations:** 1 Facultad de Ciencias Físicas y Matemáticas, Departamento de Ingeniería Química, Biotecnología y Materiales, Universidad de Chile, Beauchef 851, Santiago 8370456, Chile; 2 Dipartimento di Chimica e Chimica Industriale, Università di Pisa, Via Moruzzi 13, 56124 Pisa, Italy; marco.carlotti@unipi.it; 3 Center for Materials Interfaces, Istituto Italiano di Tecnologia Viale Rinaldo Piaggio 34, 56025 Pontedera, PI, Italy; virgilio.mattoli@iit.it; 4 Department of Chemical Product Engineering, ENTEG, University of Groningen, Nijenborgh 4, 9747 AG Groningen, The Netherlands; f.orozco.gutierrez@rug.nl (F.O.); g.de.macedo.rooweder.lima@rug.nl (G.M.R.L.); f.picchioni@rug.nl (F.P.); r.k.bose@rug.nl (R.K.B.); 5 Instituto Universitario de Investigación y Desarrollo Tecnológico (IDT), Universidad Tecnológica Metropolitana, Ignacio Valdivieso 2409, San Joaquín, Santiago 8940577, Chile; rodrigo.araya@utem.cl (R.A.-H.); dcortes@utem.cl (D.C.-A.); 6 Centro Integrativo de Biología y Química Aplicada (CIBQA), Facultad de Salud, Universidad Bernardo O’Higgins, General Gana 1702, Santiago 8370854, Chile; daniela.ortega@ubo.cl

**Keywords:** Paal-Knorr reaction, polyketone, Diels-Alder click chemistry, self-healing, shape-memory polymers

## Abstract

Furan/maleimide dynamic covalent chemistry has been extensively used to fabricate re-workable and self-healing thermosets. Understanding the relationship between crosslinker structure, network dynamics, and material final properties, however, remains a challenge. This study introduces self-healing and shape-memory thermosets derived from furan-functionalized polyketones (PKFU) crosslinked with aromatic bis-maleimides, i.e., 1,1′-(methylenedi-4,1-phenylene)bis-maleimide (BISM1) and bis(3-ethyl-5-methyl-4-maleimidophenyl)methane (BISM2), via a thermally reversible Diels-Alder reaction. Polyketones were chemically modified with furfurylamine through the Paal-Knorr reaction, achieving varying furan grafting ratios. The resulting networks, characterized by ATR-FTIR, ^1^H-NMR, gel permeation chromatography (GPC), differential scanning calorimetry (DSC), and rheology, demonstrated tunable thermomechanical properties. BISM2-based thermosets exhibited enhanced thermal stability and reversibility over a broad temperature range (20–120 °C), with a shape recovery ratio of up to 89% and complete self-healing at 120 °C within 5 min. These findings highlight the potential of polyketone-based thermosets for applications requiring adaptive thermomechanical properties, efficient self-repair, and sustainability.

## 1. Introduction

Thermosets are renowned for their exceptional mechanical properties, outperforming thermoplastics in key areas such as high modulus, excellent solvent resistance, dimensional stability, and impressive fracture strength—attributes that stem from their robust covalently crosslinked networks. These qualities allow thermosets to perform reliably under extreme conditions, making them indispensable for demanding applications [1]. However, the irreversible nature of these crosslinked networks results in inherent sustainability challenges, as they cannot be reprocessed, reshaped, recycled, or repaired [2]. These limitations have led to significant implications in managing thermoset waste. Researchers have explored various recycling strategies to address this issue, including mechanical grinding, high-temperature processing, and chemical recycling [3]. Despite these efforts, recycled products often suffer from degraded mechanical properties, and the methods themselves typically require harsh conditions [4]. As a result, most thermoset waste is reduced to low-value reinforcement fillers or subjected to thermal treatments such as pyrolysis or incineration to recover fibers or generate energy [5]. These approaches, however, fall short of achieving true sustainability.

To address these challenges, researchers have developed advanced material solutions that integrate sustainability with enhanced functionality, aiming to prolong product lifespans. A notable advancement in this field is represented by re-workable thermosets, a class of cutting-edge materials that preserve the advantageous properties of traditional thermosets while incorporating reprocessability and recyclability, characteristics typically associated with thermoplastics [6,7].

As previously discussed, another complementary approach to enhancing sustainability involves extending the lifespan of materials by preserving their functional properties and aesthetic qualities [8]. In this regard, self-healing polymeric materials have attracted considerable attention in recent years due to their remarkable capacity to autonomously repair damage and restore functionality following mechanical or thermal stress [9,10]. These advanced materials exhibit an inherent capacity to mend themselves, thereby maintaining structural integrity and extending their service life [11]. Self-healing polymers can operate autonomously, requiring no external intervention, or respond to external stimuli, with temperature being the most utilized trigger [9]. Broadly, self-healing polymers are classified into extrinsic and intrinsic systems [12]. Extrinsic self-healing polymers rely on pre-embedded healing agents [13], which are typically encapsulated within microcapsules [14] or delivered through vascular networks [15]. In contrast, intrinsic self-healing polymers are designed with functional groups that enable reversible interactions within the polymer matrix. To fabricate these reversible networks, supramolecular interactions [16] or dynamic covalent bonds are commonly used [17].

Natural hydrogels, such as alginate, chitosan, and gelatin-based systems, have been extensively investigated for biomedical applications due to their biocompatibility [18], water retention capabilities, and intrinsic self-healing properties [19,20,21]. However, their applicability in high-performance engineering applications is limited by poor mechanical strength, high water sensitivity, and restricted thermal stability [22]. Similarly, polyurea-urethane-based self-healing materials offer good mechanical recovery but often require external healing agents or microencapsulated repair systems, which may reduce long-term reliability [23]. In contrast, synthetic polymer networks utilizing dynamic covalent bonds, such as Diels-Alder (DA) chemistry, provide a robust and scalable alternative, ensuring both high mechanical performance and efficient thermal reversibility [24,25]. Indeed, the thermoreversible Diels-Alder (DA) reaction between furan (a diene) and maleimide (a dienophile) has emerged as a prominent strategy as it allows for repeatable self-healing, tunable thermomechanical properties, and enhanced durability compared to conventional polymeric systems [26]. In addition, this reaction offers several advantages: the equilibrium between the forward DA and reverse retro-DA (r-DA) reactions can be cycled repeatedly across a wide temperature range (e.g., from room temperature to 120 °C) rapid kinetics, high yield, and reversibility [27]. Moreover, the selection of specific crosslinkers can significantly influence network reversibility, mechanical flexibility, and overall healing efficiency, making it crucial to optimize both polymer composition and crosslinking architecture [24].

In this context, our group has extensively focused on self-healing thermosets utilizing the r-DA reaction, emphasizing scalable thermoreversible network production. To achieve this, we have focused on the chemical modification of polyketones with primary amines via the Paal-Knorr reaction, a straightforward and industrially viable chemical route. Polyketones are a class of engineering thermoplastic polymers obtained by copolymerizing ethylene and/or propylene with carbon monoxide [28]. Their backbone is characterized by the presence of carbonyl groups spaced by two sp^3^ carbon atoms, which give them good mechanical properties and excellent solvent resistance. This structure also opens interesting possibilities for functionalizing these polymers with various side pendant groups via the Paal-Knorr pyrrolization [29]. This latter involves forming pyrrole rings through a condensation reaction between primary amine derivatives and the polyketone 1,4 di-ketone group [30]. This approach offers several advantages: it proceeds rapidly (4 h), operates in bulk without catalysts, yields high efficiency under mild conditions (100–110 °C), and produces water as the sole by-product. Additionally, the process is highly versatile, accommodating a wide range of primary amine derivatives, making it an economical and attractive approach for synthesizing polymers with diverse functional pendant groups. Our research group has successfully developed thermoreversible self-healing polymeric networks within this context by modifying polyketones with furan groups, enabling thermally r-DA reaction with maleimide compounds. Moreover, polyketones are an excellent platform for implementing DA-based networks due to the degree of furan grafting and the choice of crosslinker, which can significantly influence the resulting material’s properties, from its glass transition temperature to its ability to undergo reversibility that leads to self-healing and shape memory macromolecular transitions. Nevertheless, understanding the relationship between crosslinker structure, network dynamics, and material final behavior remains an unresolved challenge.

**Figure 1:**
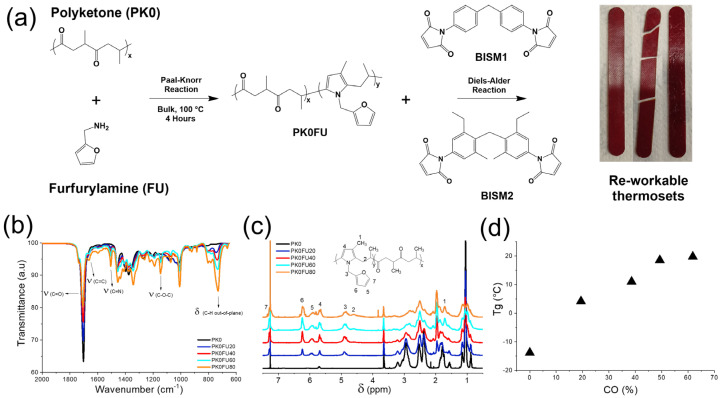
(**a**) Schematic representation of PK0 chemically modified with furfurylamine via the Paal–Knorr reaction (PK0FU), bismaleimides BISM1 and BISM2 used in the crosslinking process, and the resulting thermoreversible thermoset. (**b**) ATR–FTIR and (**c**) ^1^H–NMR spectra of PK0 and modified with FU at different CO % (PKFU20, PKFU40, PKFU60, PKFU80). (**d**) PKFUs T_g_ at different di-carbonyl conversion (CO %) calculated from DSC analysis (see Figure S2 for full thermal history and all polymer series).

In this study, we investigate the thermomechanical properties of thermosets derived from furan-functionalized polyketones crosslinked with two bismaleimides, BISM1 and BISM2 (Figure 1a), varying crosslinking densities. While BISM1 and BISM2 exhibit comparable chemical structures, the alkyl and ethyl substituents present in BISM2 can affect the torsional angle and the rigidity of the crosslinker, which, in turn, possibly affect its reactivity towards furane. In addition, unlike natural hydrogel-based self-healing materials, our approach leverages a synthetic thermoset platform with high structural stability, processability, and long-term durability, making it suitable for applications requiring recyclability, adaptability, and mechanical robustness. Notably, this work focuses on the reversibility of the crosslinking networks and the thermal and mechanical properties of these thermosets, synthesized using polyketones with different degrees of functionalization and the two distinct bismaleimides. Comprehensive material characterization was performed using differential scanning calorimetry (DSC), dynamic mechanical analysis (DMA), and rheological measurements. In addition, the shape memory behavior and self-healing capabilities of the thermosets were systematically evaluated to assess their potential for advanced functional applications. To complement the experimental data, theoretical studies were undertaken using DFT calculations to elucidate the mechanisms underlying the DA reaction, thus providing a deeper understanding of the factors influencing the reversibility and performance of the crosslinked networks. This integrated approach offers valuable insights into the design and optimization of thermally reversible thermoset systems for self-healing and shape memory applications.

## 2. Materials and Methods

### 2.1. Reagents

Furfurylamine (FU, Merck Life Sciences S.r.l., Milan, Italy) was distilled before use. 1,1-(methylenedi-4,1-phenylene)bis-maleimide (BISM1, Merck Life Sciences S.r.l., Milan, Italy), tetrahydrofuran (THF, Merck Life Sciences S.r.l., Milan, Italy), chloroform (CHL, Merck Life Sciences S.r.l., Milan, Italy) and Bis(3-ethyl-5-methyl-4-maleimidophenyl)methane (BISM2, abcr, Karlsruhe, Germany) were used as received. Polyketone composed of propylene and carbon monoxide was synthesized according to the literature [31]. Polyketone polymer (PK0) comprising 50% propylene and 50% carbonyl molar ratio units was used. CDCl_3_ (Sigma-Aldrich, Milan, Italy) was used as deuterated solvent for ^1^H-NMR investigations.

### 2.2. Functionalization of Polyketone with Furan Groups

The functionalization of polyketone with furan groups (Figure 1a) was performed following a procedure reported in the literature [32]. Different molar ratios of the 1,4-dicarbonyl functionalities of the starting polymer (PK0) and FU were employed as specified in Table S1. Thus, the grafting target of FU was defined as molar ratios in percentages with respect to the PK0 carbonyl content and named di-carbonyl conversions (CO %). This chemical modification of PK0 yielded viscous polymer products grafted with different amounts of furan groups (PK0xFUy) that were analyzed by ^1^H-NMR, ATR-FTIR, and elemental analysis.

### 2.3. Preparation of Polyketone Thermoset for Stiff-Modulated Studies

Thermally reversible thermosets were prepared using the Diels-Alder reaction between PK0 functionalized with furan groups (PK0FU) and the bismaleimides. First, PK0FU polymers were dissolved in 20 mL of THF inside a round bottom flask provided with a reflux and oil heating unit under vigorous magnetic stirring at 50 °C. Different amounts of BISM1 or BISM2 were dissolved in 25 mL of THF to reach FU:BISM molar ratios of 1:0.5, 1:1, and 1:2. The maleimide solutions were added to the polymer solution dropwise for 30 min. The reaction was set under vigorous stirring at 50 °C for 24 h. The solvent was then evaporated, and each sample was placed in a vacuum oven at 80 °C for 48 h at low pressure for drying. The resulting thermoset materials were grained and characterized by DMTA and DSC analysis.

### 2.4. Diels-Alder Computational Studies

Density functional theory (DFT) calculations were carried out by using the dispersion-corrected B3LYP [33] hybrid exchange-correlation functional using the Grimme’s DFT-D3 dispersion correction [34] with the standard 6–31G (d,p) basis set [35] in Gaussian16 (v.C.01, Gaussian, Inc., Wallingford, CT, USA, 2016). This level of theory has been shown to be suitable for analyzing both geometric and electronic properties of DA reaction [36,37,38,39,40]. The nature of all the stationary points was determined by vibrational analysis, with only one imaginary frequency mode for the transition state structures identified and no imaginary frequencies for ground states. Relative enthalpies (∆H) have been calculated based on the total energies of the stationary points at the standard statistical thermodynamics at 298.15 K and 1 atm.

### 2.5. Characterization

The elemental composition of the functionalized polymers was determined by using an Elementar Vario Micro Cube (Milano, Italy) for nitrogen, carbon, and hydrogen. ^1^H-NMR spectra were recorded at room temperature in CDCl_3_ solution with a Bruker Avance DRX 400 spectrometer (Bruker, Billerica, MA, USA), using the residual solvent peak as the internal reference. ATR–FT–IR spectra were recorded using a Perkin-Elmer Spectrum One with a diamond crystal detector (Perkin-Elmer, Waltham, MA, USA), a resolution of 4 cm^−1^ within a spectral range of the 4000–650 cm^−1^ and averaged over 32 scans. Differential scanning calorimetry (DSC, TA Instruments, New Castle, DE, USA) was carried out using a TA DSC250 under N_2_. For polymer thermal analysis, only two cycles were performed, and for thermosets’ DA-rDA analysis, 4 cycles were performed. We obtained the T_g_ of the polymer and enthalpy of the rDA reaction using the software TRIOS v6.1 (TA Instruments (New Castle, DE, USA). Gel Permeation Chromatography (GPC) measurements were performed with an HP1100 Hewlett-Packard (Hewlett-Packard, Wilmington, Philadelphia, PA, USA) using chloroform as eluent and polystyrene as standards. Thermomechanical analyses were conducted on a Perkin Elmer Dynamic Mechanical Analyzer DMA 8000 (Waltham, MA, USA) using single cantilever mode at an oscillation frequency of 1 Hz and a heating rate of 3 °C/min. The samples for DMA analysis were prepared by graining 20 mg of the sample and placing it on a Metallic Pocket system [41]. Rheology measurements were performed with a Discovery HR-2 rheometer from TA Instruments (New Castle, DE, USA) with TRIOS v5.1.1.46572 software (TA Instruments (New Castle, DE, USA)). The samples were prepared by compression molding of grinded thermoset materials into uniform discs with 8 mm diameter and 1 mm of thickness at 120 °C for 20 min. Then, the samples were placed in an oven at 50 °C overnight for DA crosslinking and thermoset consolidation before analysis. The samples were measured using parallel plates of the same diameter pressing the sample with a constant axial force of 2 N. The temperature scans between 120 and 40 °C (0.5 K/min) were carried out in oscillation mode with 0.05% strain and 1 Hz. Amplitude sweeps were also carried out at 120 and 40 °C to verify that the strain percentage was within the viscoelastic linear regime. The first heating scan was conducted to erase the thermal history of the thermoset. Shape memory was characterized on a Rheometer (Discovery HR20, TA Instruments, New Castle, DE, USA) with Clamps (Rectangular solid sample—48158). The data were analyzed with the software TRIOS v5.1.1.46572 (TA Instruments, New Castle, DE, USA). The samples were set at 80 °C and deformed at 3.14 rad to perform the experiments. Subsequently, the deformation was kept for 3600 s until the sample cooled down to room temperature. Finally, the deformation stress was removed, and the temperature was increased to 80 °C for 40 min. Shape recovery rate = (fixed angle-recovered angle)/fixed angle.

## 3. Results and Discussion

### 3.1. Polyketone Functionalized with Furan Groups via the Paal-Knorr Reaction

We successfully grafted furan groups onto polyketone (PK0) by reacting the polymer with furfurylamine (FU) through the Paal-Knorr reaction [32]. The PK0FU samples were named PK0FU20, PK0FU40, PK0FU60, and PK0FU80 according to the target FU molar ratios in percentages with respect to the PK carbonyl content. The prepared four polymer products reached a final di-carbonyl conversion (CO %) of 19.4%, 38.6%, 49.3%, and 61.8%, respectively (Table 1, refers to Table S1 for the stoichiometry of reactants and calculations). The total di-carbonyl efficiency was determined through elemental analysis based on the relative nitrogen content in the products (Table S1).

We evaluated the extent of the functionalization of PK0 with FU using ATR-FTIR and ^1^H-NMR spectroscopies. Figure 1b illustrates the 2000–600 cm^−1^ region of the FTIR spectra, comparing the unmodified PK with its FU-functionalized derivatives at various CO %. As the CO % increased from PK0FU20 to PK0FU80, the intensity of the carbonyl group around 1700 cm^−1^ decreased, indicating the progressive disappearance of the 1,4-dicarbonyl moieties following the formation of pyrrole rings through the Paal-Knorr reaction. Simultaneously, the four modified polyketones exhibited two notable peaks at 730 cm^−1^ and 1010 cm^−1^, corresponding to out-of-plane proton bending and C-O-C stretching from the furan pendant group. Signals at 1650 cm^−1^ and 1500 cm^−1^ were attributed to the C=C and C=N bonds of the furan and pyrrole rings. The full spectra of these materials are presented in Figure S1, where additional signals at 3100 cm^−1^, 2969, and 2873 cm^−1^, were ascribed to the C-H stretching of the pyrrole ring and to the asymmetric and symmetric stretching bands of the aliphatic C-H groups of PK0, respectively. Overall, the decrease in the intensity of the carbonyl group and the increase in that of the peaks at 730 cm^−1^ and 1010 cm^−1^ indicates the successful grafting of PK0 with furan groups.

Figure 1c shows the ^1^H-NMR spectra of PK0 before and after functionalization with FU at different di-carbonyl conversions. The appearance of new proton signals in the spectra of functionalized PKFUs indicates the successful polymer modification. These signals were attributed to the furan and pyrrole rings formed during the Paal-Knorr reaction, along with the adjacent methyl and methylene groups, as highlighted in the inset of the figure. The ^1^H-NMR spectra of PK0FUs indicate signals attributed to the formation of pyrrole rings around 5.7 ppm (H4). Peaks at 4.9 (H3), 5.9 (H5), 6.2 (H6), and 7.3 ppm (H7) corroborate the presence of furan groups.

Finally, Figure 1d shows the glass transition temperature (T_g_) of PK0FU polymers as a function of CO %. The observed ascending trend indicates that an increased formation of pyrrole groups within the polymer backbone and furan pendant groups enhances the rigidity of the chains. This behavior is attributed to the planar structures of the pyrroles and furan heterocycles, which contribute to restricting molecular mobility due to π-π stacking and electrostatic interactions.

### 3.2. Preparation of Polyketones Thermoset via Diels-Alder Crosslinking with BISM1 and BISM2

The presence of furane groups in PK0FUs enables the use of bismaleimides to crosslink the polymer via Diels-Alder (DA) cycloaddition. Given the thermally reversible nature of the DA reaction, this approach has been widely utilized to create thermally reversible polymer networks [42]. The thermomechanical response and reversibility of the polyketone-based thermosets are possibly influenced by the choice of crosslinker, BISM1 or BISM2. Despite their structural similarities, the presence of alkyl (ethyl and methyl) substituents in BISM2 could result in notable differences in crosslinking behavior.

We prepared different polyketone thermosets by crosslinking PK0FU20, 40, 60, and 80 with two bismaleimides, BISM1 and BISM2 (structures are reported in Figure 1a). These dienophile crosslinkers feature a similar diphenylmethane core; however, the substituents in the ortho-position of BISM2 significantly impact their torsional angle, rigidity, reactivity, and selectivity in forming exo and endo adduct [27]. These aspects will be explored in detail later in this section. Specifically, we investigated molar ratios of 1:0.5, 1:1, and 1:2 (FU:BISM), maintaining a constant number of furan groups while varying the quantities of BISM1 and BISM2 to achieve the desired ratios and crosslink densities.

We used differential scanning calorimetry (DSC) to investigate the thermal transition of the thermosets, focusing on the T_g_ and the dynamics of the rDA reaction within the crosslinked systems. The thermosets prepared with PK0FU20, crosslinked with either BISM1 or BISM2 at all molar ratios (Figure S3), as well as PK0FU40, crosslinked with either BISM1 or BISM2 synthesized at a 1:0.5 FU:BISM ratio (Figure S4), exhibited two distinct transitions. The first transition is attributed to the T_g_ of non-crosslinked segments of the polymers. It is observed in the range of 20–50 °C, while the second and broad endothermic transition observed between 80 to 160 °C points out to the rDA reaction [27]. In contrast, the polymer networks produced with PK0FU40, 60, and 80 and crosslinked with BISM1 or BISM2 showed the endothermic transition between 80 to 160 °C related to the rDA reaction (Figures S4–S6).

**Figure 2:**
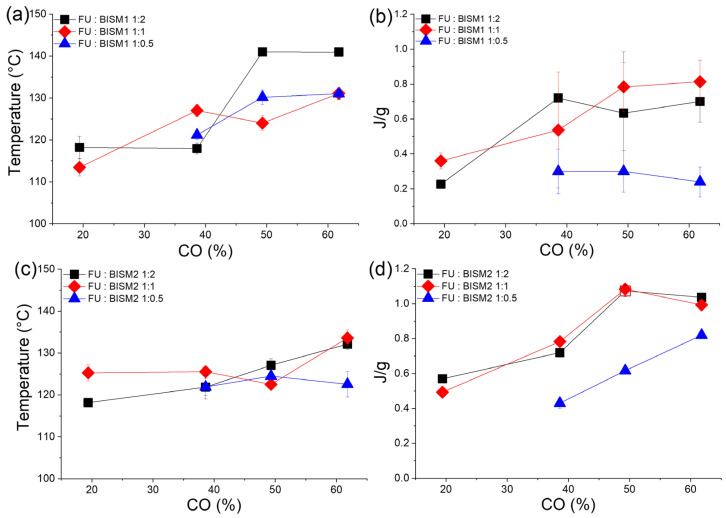
(**a**) temperature and (**b**) energy required for rDA reaction in polyketone thermosets crosslinked with BISM1 and (**c**) temperature and (**d**) energy required for rDA reaction in polyketone thermosets crosslinked with BISM2, plotted as a function of carbonyl conversion.

The systems exhibited notable differences between BISM1 and BISM2. DSC results indicate that BISM2-based thermosets exhibit superior reversibility across multiple heating cycles compared to BISM1-based systems. Specifically, BISM1 networks show a gradual reduction in the endothermic peak intensity associated with the retro-Diels-Alder (rDA) reaction upon successive heating cycles (Figures S3–S6). This is attributed to the incomplete reformation of DA adducts during the cooling process or the loss of furan/maleimide reversible functionality over multiple cycles due to possible side reactions that compromise network integrity [43]. In contrast, BISM2 crosslinked thermosets maintain consistent reversibility, likely due to the increased regioselectivity and stability of the DA adducts, as suggested by the higher enthalpy values required for the rDA reaction (Figures S3–S6). This stability strongly supports the hypothesis that the crosslinking process mediated by the DA reaction with BISM2 is both highly reversible and repeatable under the tested conditions. We further analyzed the temperature and energy required for the rDA reaction for both BISM1 and BISM2 as a function of the carbonyl conversion in polyketone, which correlates with an increase in polymer T_g_, crosslinking density, and consequently, polymer matrix rigidity. As reported by Orozco et al. [27] the thermo-reversibility of the DA process in polyketone thermosets is faster and favored at lower temperatures in matrices with lower crosslinking densities and with BISM1 as a crosslinker. Figure 2 illustrates the peak temperature and energy required for the rDA reaction in systems using BISM1 and BISM2. As discussed above, both systems exhibit an upward trend in onset temperature and energy requirements as a function of CO % and crosslinking densities, which is related to the rigidity of the polymer networks. However, the trend is less pronounced for BISM2, indicating that its rDA reaction is less affected by increased rigidity. Notably, in systems with a 1:0.5 ratio, the temperature required for the rDA reaction with BISM2 remains constant, regardless of the degree of conversion crosslinking density. This suggests that BISM2 provides a more adaptable response, maintaining effective reversibility even as the polymer network becomes more rigid. Moreover, enthalpy values are higher for networks containing BISM2. It might be explained by the ethyl and methyl groups present in BISM2 conferring more regioselectivity and stability of DA adduct. The latter required more energy for rDA reaction reflected as a higher enthalpy (Figure 2d).

### 3.3. Diels-Alder Reaction Theoretical Studies

**Figure 3:**
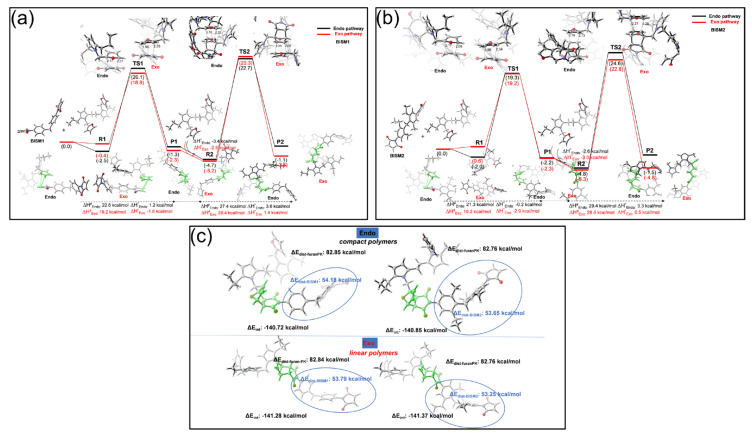
(**a**) Enthalpy reaction pathways of endo-(black) and exo−(red) stereoisomers for the Diels-Alder cycloaddition reactions with BISM1 as a dienophile. (**b**) Enthalpy reaction pathways of endo-(black) and exo−(red) stereoisomers for the Diels−Alder cycloaddition reactions with BISM2 as a dienophile. (**c**) Distortion-interaction analysis in the P1 endo and exo product structures according to Figure 3a,b. Fragment 1 is the BISM1 or BISM2 dienophiles, and fragment 2 is the furan−PK diene. ΔE_dist−BISM1_ and ΔE_dist−BISM2_ represent the distortion energy of BISM1 and BISM2 dienophiles, respectively, while ΔE_dist−furanPK_ represents the distortion energy of diene. ΔE_int represents_ the interaction energy between each dienophile and diene molecule.

To answer the latter uncertainties, we explored the dynamics of the DA reaction in our system by the enthalpy formation of the endo- and exo-stereoisomers using a computational assessment through DFT calculations (Figure 3a,b; details are given in Supplementary Materials in Table S3).

The DA reaction mechanism between the furan-PK (diene) and the dienophiles BISM1 (Figure 3a) and BISM2 (Figure 3b) provides a detailed understanding of the energy profiles associated with each step of the process. For both dienophiles, the initial endo-approaches are thermodynamically more stable than their exo-counterparts, with enthalpic differences of −2.5 kcal/mol vs. −0.4 kcal/mol for BISM1 (Figure 3a) and −2.0 kcal/mol vs. 0.6 kcal/mol for BISM2 (Figure 3b). However, the activation barriers (ΔH^ǂ^) for the formation of the exo products are slightly lower in both cases, being 19.2 kcal/mol compared to 22.6 kcal/mol in the endo pathway for BISM1 (Figure 3a) and 19.2 kcal/mol vs. 21.3 kcal/mol for BISM2 (Figure 3b). These results suggest that while the endo approach is thermodynamically preferred in the pre-transition state region, the formation of exo products is kinetically more accessible.

The reaction energies (ΔH°) further demonstrate differentiated behaviors: for BISM1, the exo product is exothermic (−1.8 kcal/mol), whereas the endo product is slightly endothermic (1.2 kcal/mol). In BISM2, both products are exothermic, with the exo product being more stable (−2.9 kcal/mol) than the endo product (−0.2 kcal/mol). These differences in final energetic stability highlight a greater tendency toward forming exo products for both dienophiles, with BISM2 showing relatively higher stability.

Density Functional Theory (DFT) calculations highlight that BISM2 favors exo-stereoisomer formation with a slightly lower activation barrier than BISM1. This kinetic preference may enhance the crosslinking efficiency and contribute to the observed thermal stability and self-healing efficiency. The reduced distortion energy of BISM2-containing DA adducts results in lower steric hindrance, which is crucial for maintaining network integrity over multiple thermal cycles. Given that self-healing relies on the reversible dissociation and reformation of the Diels-Alder bonds, the regioselectivity may play a role in the efficiency of the healing process. However, due to the complex interplay between network mobility and stereoisomeric composition, a precise determination of the preferred endo/exo ratio for optimal healing efficiency requires additional structural and kinetic studies beyond the scope of this work.

This result aligns with experimental observations, which indicate a trend toward more linear and less folded polymer structures for BISM2 network systems, characteristic of exo regioselectivity (see Figure 3c). The structural linearity of the polymer in exo products suggests lower distortion energies (ΔE_dist_) compared to endo products (Figure 3c), reinforcing the kinetic and thermodynamic preference for this regioselectivity. The main difference between BISM1 and BISM2 lies in their substituents. BISM2, with ethyl and methyl groups in peripheral positions, exhibits lower structural rigidity than BISM1, which has a rigid phenylene-based core. This contributes to lower distortion energies in the networks formed with BISM2, favoring the stability of the exo products. Additionally, the larger flexibility of BISM2 could decrease the steric hindrance during product formation, reducing activation barriers and stabilizing the final products. These structural differences are also consistent with experimental evidence showing larger thermal reversibility in systems employing BISM2. This behavior can be explained by the lower distortion energies and reduced rigidity of the dienophile, which facilitate the dissociation of products in reversible reactions such as rDA.

To study the different crosslinking densities of network systems, we also explored the potential for double Diels-Alder (DDA) reactions in both BISM1 (Figure 3a) and BISM2 (Figure 3b). The mechanism involves the successive reaction of two diene molecules with the bis-maleimide core. For BISM1, the calculated activation barriers for the DDA pathway were slightly higher compared to the single DA reaction, with values of 27.4 kcal/mol for the endo regioselectivity and 28.4 kcal/mol for the exo (R2 → P2; Figure 3a). Thermodynamically, the DDA pathway for BISM1 is less favorable than the single reaction (R1 → P1; Figure 3a), with positive reaction energies of 3.6 kcal/mol (endo) and 1.4 kcal/mol (exo). These values indicate that, while kinetically accessible, the DDA products formed with BISM1 are energetically less stable and, therefore, less likely to accumulate under standard reaction conditions.

For BISM2 (Figure 3b), the DDA pathway shows slightly lower activation barriers compared to BISM1, with 29.4 kcal/mol for the endo approach and 28.0 kcal/mol for the exo approach. Additionally, the reaction energies are less unfavorable for BISM2, with 3.3 kcal/mol (endo) and 0.5 kcal/mol (exo). These differences suggest that BISM2 is slightly more accommodating to DDA pathways due to its structural flexibility and reduced distortion energy in the final products (Figure 3c). However, the endothermicity reaction energies for both regioselectivities imply that DDA products, while feasible, are not significantly favored thermodynamically.

### 3.4. Thermomechanical Studies of Furan Functionalized Polyketones Crosslinked with BISM2

**Figure 4:**
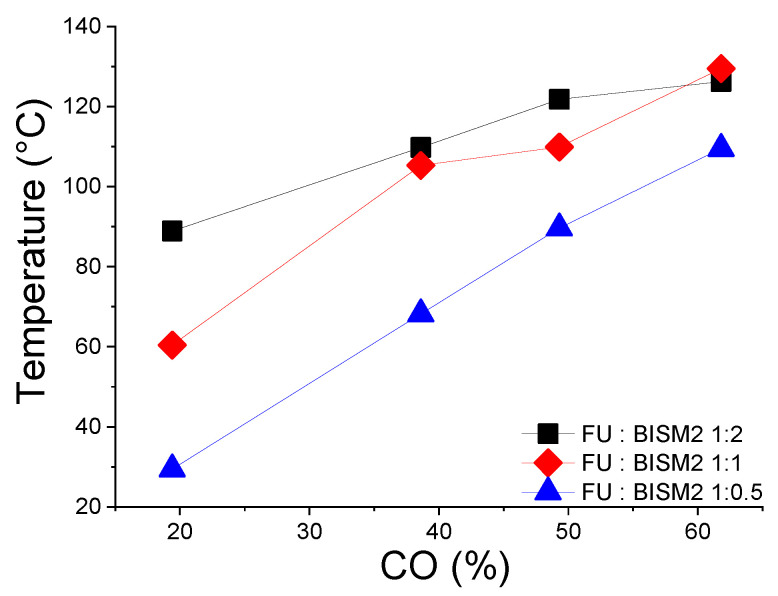
Softening point trend of the polyketone thermosets at different conversion percentages.

We further investigated the thermomechanical properties of the promising PKFU:BISM2 thermosets via DMA analysis across several compositions. BISM2 was selected for its superior reversibility and performance, as evidenced by the DSC and DFT analysis. While the closed-pocket design of Pocket DMA can trap residual solvent, potentially acting as a plasticizer and affecting T_g_, it nonetheless provided valuable insights into the viscoelastic trends of these thermosets over a 0–180 °C temperature range. In Figure S7, storage modulus (E′) and loss modulus (E″) trends are shown and illustrate that lower crosslinked thermosets (PK0FU20 and PK0FU40) display two E′ transitions, one due to the T_g_ and one related to the rDA at a higher temperature. Higher crosslinked samples (PK0FU40 and PK0FU60) show only one transition at the rDA temperature, reflecting greater rigidity. As the furan content increases from PK0FU20 to PK0FU80, T_g_ shifts upwards, indicating a progressive increase in polymer rigidity due to enhanced crosslinking density (Figure 4 (tan δ; raw data available in Figure S8)). This effect restricts molecular mobility and enhances thermal stability. Notably, samples with higher BISM2 ratios exhibit an even greater improvement in thermal stability, particularly when fabricated with polymers at higher carbonyl conversion (CO %). These findings are consistent with the trends observed in both DMA and DSC analyses.

By adjusting the carbonyl conversion and BISM2 ratios, the softening points can be finely tuned, enabling the design of materials tailored for applications requiring specific thermal and mechanical properties. These findings correlate with the DA/rDA equilibrium observed in Figure 2, as the thermally reversible nature of the DA crosslinks directly influences the mechanical relaxation behavior and, consequently, the self-healing efficiency of the material. The structural flexibility of BISM2, in particular, contributes to improved reversibility and self-repair capabilities, as observed in the healing tests (Figure 8).

**Figure 5:**
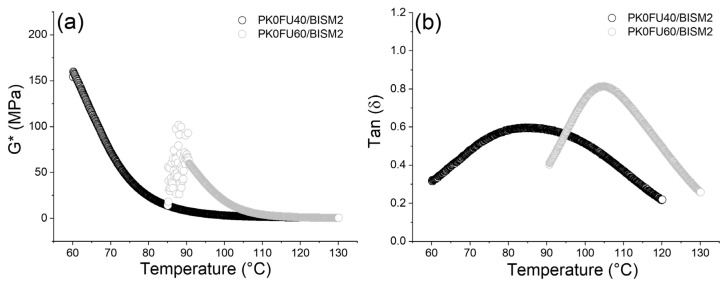
(**a**) Thermomechanical properties (complex modulus G*) and (**b**) Softening point (Tan δ) of the PK0FU40 and PK0FU60 crosslinked at a molar ratio of 1:0.5 FU:BISM with BISM1 and BISM2 determined by rheology.

To deepen the understanding of these systems, we also performed the rheological characterization of PK0FU40 and PK0FU60 crosslinked with BISM2 at a crosslinking ratio of 1:0.5 FU:BISM to corroborate the findings from DSC and DMA discussed above. Rheological measurements were conducted over a temperature range of 60–130 °C. Below 60 °C, sample slippage occurred between the rheometer plates, while above 130 °C, the samples softened excessively, preventing consistent data collection. As expected, and based on the DSC and DMA results, the thermo-mechanical profiles of the samples (Figure S9) showed high reproducibility across heating-cooling cycles and between replicates. This consistency highlights the reliability of material behavior and the reversibility of DA and rDA reactions. Figure 5a shows the sample complex modulus (G*) as a function of temperature (only the second heating cycle is shown for brevity). G* represents both storage G′ and loss G′′ moduli becomes smaller at higher temperatures. This decrease is due to the increased mobility of the polymer chains within the amorphous regions, aligning with the low T_g_ around 40–50 °C for PK0FU40 (Figure S4). Furthermore, at temperatures above 90 °C, the samples start undergoing partial decrosslinking via the rDA reaction, consistent with the DSC data (Figures S4 and S5). Above T_g_, PK0FU40 becomes a soft rubbery material, while PK0FU60 transitions similarly when the rDA process initiates. Additionally, Figure 5b shows the Tan δ values for PK0FU40 and PK0FU60 samples produced with BISM2. The thermoset prepared with PK0FU60 exhibited a Tan δ peak at a higher temperature than PK0FU40, indicating greater crosslinking and rigidity, which aligns with the previous results (Figure 4). Both samples also exhibited a softening point approximately 20 °C higher than that observed in DMA measurements. This discrepancy, as previously explained, is attributed to the sensitivity of the pocket system to residual solvents.

When the CO % increases, T_g_ shifts upwards, indicating a progressive increase in polymer rigidity due to enhanced crosslinking density. However, in BISM2-based thermosets, this effect is less pronounced than in BISM1, suggesting that the steric hindrance of the crosslinker mitigates excessive stiffness and allows for a more uniform network distribution.

**Figure 6:**
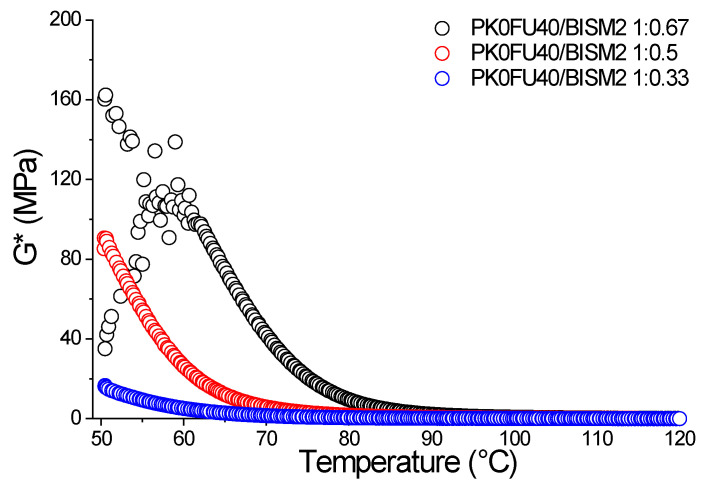
Thermomechanical properties (complex modulus G*) PK0FU40 crosslinked with BISM2 at different molar ratios 1:0.67, 1:0.5, and 1:0.33 FU: BISM determined by rheology.

We prepared six new thermosets based on PK0FU40 with reduced BISM2 loadings—ratios of 1:0.67, 1:0.5, and 1:0.33 FU:BISM—designed to allow greater molecular mobility and provide deeper insights into the crosslinking mechanism. These systems were subsequently analyzed using rheology and DSC. Figure 6 presents the rheological measurements performed on the thermosets within the 50–120 °C temperature range, selected to ensure consistent and reliable data collection. For clarity and brevity, only the results from the second heating cycle are displayed. A higher crosslinking density, indicated by a lower FU:BISM ratio, provides a higher G* value and shifts the thermoreversible process to higher temperatures, as expected from the earlier results. Conversely, thermoreversible behavior occurs at lower temperatures in thermosets with reduced crosslinking density, consistent with the DSC data shown in Figure 2. We can expect that, since BISM2 has more steric hindrance on the aromatic rings than BISM1 (Figure 1a, complex modulus G* of thermoset also fabricated with BISM1 in Figure S10), it is less susceptible to forming aromatic-aromatic interactions, which would provide additional crosslinking points (although weaker). On the other hand, this difference is not evident for the formulations with higher crosslinking densities, likely because the extended covalent crosslinking dominates the effect of weaker aromatic interactions (Figure S10).

**Figure 7:**
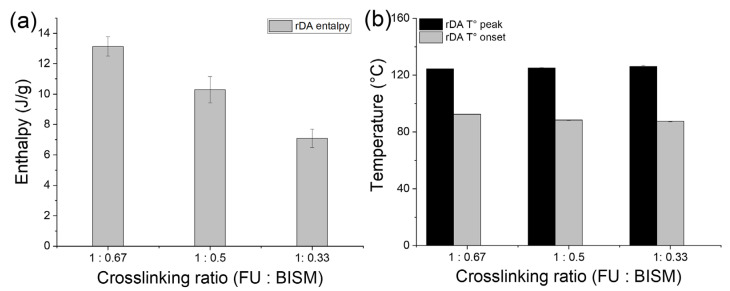
(**a**) Energy required for rDA reaction, (**b**) maximum temperature energy required for rDA reaction, onset of the temperature required for rDA, and T_g_ for thermosets composed by PK0FU40 crosslinked with BISM2 at different molar ratios 1:0.67, 1:0.5, and 1:0.33 FU:BISM determined by DSC.

The crosslinking reversibility of the thermosets was assessed via DSC within a temperature range of −20 to 150 °C at a heating rate of 5 °C/min, focusing on the rDA reaction to provide a clear understanding of the reversibility (Figure 7). As noted earlier, thermosets with higher crosslinking density require greater energy for the rDA reaction than those with lower crosslinking density (Figure 7a). Interestingly, no differences were observed in the temperature peak and onset of the rDA reaction among the thermosets (Figure 7b), suggesting that polymer chain mobility plays a critical role in the reversibility of DA/rDA reaction [27] and the robustness of the thermal crosslinking reversibility of the PK0FU40/BISM2 system is evident. Notably, the onset temperature of the rDA reaction varies significantly depending on whether BISM1 or BISM2 is utilized (Figure S11C). Systems incorporating BISM2 demonstrate a significantly higher onset temperature—approximately 20 °C higher—than those utilizing BISM1. This phenomenon is likely attributed to the stereoselective and more stable conformation of FU/BISM2 crosslinking, as proposed in the rheological and DFT calculation studies. These findings corroborate the influence of the crosslinker type, BISM1 or BISM2, on the thermal behavior and reversibility of the DA-rDA reaction.

Dynamic Mechanical Analysis (DMA) and rheology confirm that BISM2-crosslinked thermosets exhibit a more gradual modulus transition compared to BISM1, reflecting a higher degree of flexibility. This can be attributed to the steric effects of the ethyl and methyl groups in BISM2, which reduce aromatic stacking interactions, thereby preventing excessive stiffening of the network. The increased structural flexibility of BISM2 facilitates more efficient self-healing and shape memory performance by allowing a more effective macromolecular rearrangement upon thermal activation.

### 3.5. Shape Memory Effect and Thermosets Self-Healing Features

We selected the thermoset synthesized from PK0FU40 and BISM2 at a 1:0.5 (FU:BISM) crosslinking molar ratio to investigate the self-healing and shape-memory properties. This material was selected for its exceptional reversibility in the DA/rDA reaction, combined with a low softening temperature of approximately 40–50 °C. These characteristics indicate high macromolecular chain mobility and efficient network dynamics, as documented in the literature [44].

Figure S12 illustrates the shape memory behavior of the selected thermoset. Initially, the material was heated to 80 °C, above its T_g_ (as determined by DSC; Figure S4), but still below the rDA maximum temperature. The thermoset was then deformed by applying a 180° twist (π rad) for 3600 s. To fix the deformation, the sample was cooled to room temperature, effectively freezing the polymer network and locking the temporary shape (Figure S13). Finally, the shape recovery was initiated by heating the material back to 80 °C and removing the deformation stress [44]. The recovery process was monitored over 40 min, as shown in the second phase of the test. As observed, the thermoset demonstrated a high extent of recovery (Figure S13), with the material returning almost entirely to its original shape. This test was repeated 4 times (Table 2), and the average shape recovery ratio for the thermoset was calculated to be 0.89 ± 0.09, confirming its robust shape-memory capability. This performance highlights the suitability of the PKFU40 and BISM2 thermoset for applications requiring high recovery efficiency combined with thermal stability and reprocessability.

DSC and rheological data confirm that lower furan grafting ratios (PK0FU20 and PK0FU40) exhibit broader rDA transitions, indicative of a more dynamic and flexible network. This may lead to enhanced self-healing capabilities due to increased chain mobility at moderate temperatures (~120 °C). Conversely, higher grafting ratios (PK0FU60 and PK0FU80) result in sharper rDA transitions and increased energy requirements for crosslink cleavage, implying that the denser network structure restricts segmental motion, thus may slightly reducing self-healing efficiency

**Figure 8:**
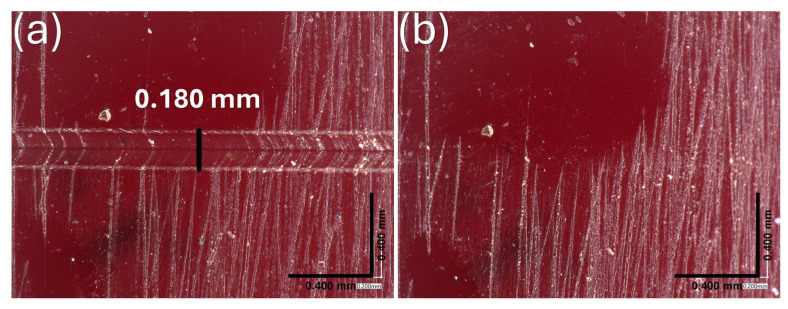
Self-healing test of thermoset PK0FU40 and BISM2 at a 1:0.5 (FU:BISM) at a 1:0.5 (FU:BISM) crosslinking molar ratio after (**a**) scratching and (**b**) thermal treatment at 120 °C for 5 min.

The self-healing process of the thermoset was initially tested at 100 °C for 20 min, but no significant repair was observed (Figure S14). However, when the material was exposed to an elevated temperature of 120 °C for 5 min, it exhibited effective crack healing, demonstrating its thermally activated self-repair capability (Figure 8).

The scratch (of 0.180 mm) was completely repaired, achieving a healing efficiency of 100%. While the exact role of endo/exo selectivity in the healing process remains to be fully elucidated, our results suggest that the thermally activated mobility of the crosslinked network, rather than stereochemical preference alone, plays a dominant role in achieving efficient self-repair. This is consistent with previous studies showing that self-healing in DA-based networks is primarily driven by crosslinking density and dynamic exchange kinetics [19]. These observations align with the differential scanning calorimetry (DSC) results, which reveal an rDA peak at 120 °C. This temperature corresponds to the point at which the crosslinked network dissociates effectively, enabling macromolecular chain rearrangement and reforming covalent bonds necessary for self-healing without side reactions [45].

To provide concrete evidence of the self-healing capabilities of the polyketone thermosets, we systematically evaluated the recovery of mechanical properties after thermal treatment at 120 °C for 5 min (Table 3). The assessment was performed using rheological tests to quantify the extent to which the material regains its original stiffness and elasticity following damage. To assess the restoration of mechanical integrity, rheology tests and DMA measurements were performed on PK0FU40-BISM2 (1:0.5 FU:BISM). Modulus E′ and E″ before and after treatment were used as a key indicator of the material recovery. The complex modulus (G*) was monitored over multiple thermal cycles. After stress and thermal treatment, the samples’ modulus was restored to ~95% of its original value, highlighting the efficiency of the reversible Diels-Alder (DA) network in re-establishing crosslinking interactions. DMA experiments confirmed that the storage and loss moduli remained consistent across multiple thermal cycles, demonstrating the material’s ability to recover its mechanical strength without significant degradation, as similarly reported for the same polyketone materials also doped by graphitic fillers [46]. Furthermore, rheology tests and DMA measurements demonstrated that the tan δ peak position remained virtually unchanged, indicating that the material’s viscoelastic properties were effectively preserved even after mechanical and thermal stress. The healing process was highly repeatable, with three consecutive damage-healing cycles demonstrating consistent mechanical recovery of at least 90% in each cycle.

The self-healing thermosets demonstrated a high level of mechanical recovery, with restoration of stiffness, strength, and elasticity exceeding 90% across different evaluation methods. The strong correlation between rheological and DMA underscores the efficiency of the thermoreversible Diels-Alder crosslinks in enabling rapid and effective self-repair. These findings reinforce the potential of the PK0FU-BISM2 thermosets for applications requiring durable, self-healing materials with repeatable mechanical recovery.

## 4. Conclusions

This study successfully demonstrated the synthesis of thermally reversible, self-healing, and shape-memory polyketone-based thermosets using furan-functionalized polyketones crosslinked via Diels-Alder reactions with two bismaleimides, BISM1 and BISM2. The functionalization of polyketones through the Paal-Knorr reaction provided a versatile platform to incorporate furan groups, achieving tailored crosslinking densities and enhanced thermomechanical properties. Detailed characterization revealed that the choice of crosslinker significantly impacts the thermal reversibility, rigidity, and overall performance of the thermosets.

The computational and experimental findings highlight the influence of dienophile structural features on the mechanism and regioselectivity of Diels-Alder reactions. While both systems favor exo regioselectivity, BISM2 demonstrates more favorable thermal and kinetic properties, making it a more efficient dienophile for applications in reversible materials. The higher activation barriers and less favorable reaction energies for DDA pathways in both dienophiles (BISM1 and BISM2) suggest that these reactions are less likely to dominate under standard experimental conditions. However, the slight energetic advantage of the exo approach in BISM2 reinforces its overall preference for exo regioselectivity. The ability of BISM2 to better accommodate steric demands during the DDA reaction may also reflect its superior thermal reversibility, as observed experimentally.

BISM2-based thermosets exhibited superior dynamic properties, including higher thermal stability, better crosslinking reversibility, and a remarkable shape-memory recovery ratio of up to 90%. Additionally, self-healing tests confirmed rapid and complete crack repair at 120 °C within 5 min, highlighting the effectiveness of the reversible Diels-Alder network in maintaining structural integrity under thermal stress. These findings open exciting possibilities for using these materials in adaptive technologies, such as soft robotics, flexible electronics, smart coatings, and reconfigurable actuators, where materials that can respond to stimuli, self-heal, and adapt their mechanical properties are crucial. The reversible Diels-Alder crosslinking not only enhances their performance but also makes them promising candidates for sustainable polymeric solutions, allowing for recyclability, repairability, and extended durability. By offering a deeper understanding of reversible polymer networks, this study lays the groundwork for future advancements in areas like energy storage, biomedical scaffolds, and next-generation structural composites, where smart and sustainable materials are increasingly in demand.

## Figures and Tables

**Table 1:** Elemental analysis of PK alone and chemically modified with FU at different di-carbonyl conversions.

Sample	Molar Feed Ratio FU/CO	Experimental Di-Carbonyl Conversion (%) ^1^
PK0	0	-
PK0FU20	0.2	19.4
PK0FU40	0.4	38.6
PK0FU60	0.6	49.3
PK0FU80	0.8	61.8

^1^ Di-carbonyl conversion (CO %) obtained from EA.

**Table 2:** Shape recovery of the thermoset synthesized from PK0FU40 and BISM2 at a 1:0.5 (FU:BISM) crosslinking molar ratio.

Sample	Fixed Deformation (rad)	Recovered Shape (rad)	Shape Recovery Ratio
1	3.14	0.3	0.90
2	3.14	0.22	0.93
3	3.14	0.08	0.97
4	3.14	0.74	0.76

**Table 3:** The table below summarizes the mechanical property recovery efficiency.

Property	Pristine Sample	Post-Healing	Recovery Efficiency (%)
Complex Modulus (G*)	100% (baseline)	~95%	95
E′, E″	100% (baseline)	~90–95%	90

## Data Availability

The original contributions presented in this study are included in the article/Supplementary Materials. Further inquiries can be directed to the corresponding author(s).

## Supplementary Materials

The following supporting information can be downloaded at: https://www.mdpi.com/article/10.3390/polym17050565/s1, Figure S1. ATR-FT-IR spectrum of PK chemically modified with FU at different CO %; Figure S2. DSC first cycle after thermal history erase of PK0 (PK) grafted with FU at different CO %; Figure S3. DSC thermal cycles of PK0FU20 series crosslinked with BISM1 or BISM2 at different molar ratios; Figure S4. DSC thermal cycles of PK0FU40 series cross-linked with BISM1 or BISM2 at different molar ratios; Figure S5. DSC thermal cycles of PK0FU60 series cross-linked with BISM1 or BISM2 at different molar ratios; Figure S6. DSC thermal cycles of PK0FU80 series cross-linked with BISM1 or BISM2 at different molar ratios; Figure S7. Modulus E′ and E″ of PK0FU20, PK0FU40, PK0FU60, and PK0FU80 crosslinked at different molar ratios with BISM2; Figure S8. Tan (δ) of PK0FU20, PK0FU40, PK0FU60, and PK0FU80 crosslinked at the different molar ratios with BISM2; Figure S9. Complex modulus and Tan δ of PK0FU40 crosslinked with BISM1 and BISM2 at a molar ratio of 1:0.5. Complex modulus and Tan δ of PK0FU60 crosslinked with BISM1 and BISM2 at a molar ratio of 1:0.5; Figure S10. Thermomechanical properties (complex modulus G*) PK0FU40 crosslinked with BISM1 and BISM2 at different molar ratios determined by rheology; Figure S11. Energy required for the retro-Diels-Alder (rDA), maximum temperature energy required for the retro-Diels-Alder (rDA), onset of the temperature required for the retro-Diels-Alder (rDA) for the thermoset composed by PKFU_B crosslinked with BISM1 and BISM2 at different molar ratios determined by DSC; Figure S12. Shape memory analysis of thermoset composed of PK0FU40 and BISM2 at a 1:0.5 (FU:BISM); Figure S13. Shape memory analysis of thermoset composed of PK0FU40 and BISM2 at a 1:0.5 (FU:BISM); Figure S14. Self-healing test of thermoset PK0FU40 and BISM2 at 100 °C for and 20 min; Table S1: Composition of polyketones functionalized with FU; Table S2. GPC measurements of PK alone and chemically modified with FU at different di-carbonyl conversions; Table S3. Cartesian coordinates of the optimized structures; Reference [47] is cited in the Supplementary Materials.
