# Supplementary material for: Tailoring Thermomechanical, Shape Memory and Self-Healing Properties of Furan-Based Polyketone via Diels-Alder Chemistry with Different Bismaleimide Crosslinkers
Source: Polymers (Basel). 2025 Feb 20;17(5):565. doi: 10.3390/polym17050565 (PMC11902186; doi:10.3390/polym17050565)
Supplement: Supplementary file 1 [file polymers-17-00565-s001.zip › polymers-3479907-supplementary.pdf]

Tailoring Thermomechanical, Shape Memory and Self-Healing Properties of Furan-Based Polyketone via Diels-Alder Chemistry with Different Bismaleimide Crosslinkers

Esteban Araya-Hermosilla [1,*], Marco Carlotti [2,3], Felipe Orozco [4], Guilherme Macedo R. Lima [4], Rodrigo Araya-Hermosilla [5], Daniela E. Ortega [6], Diego Cortés-Arriagada [5], Francesco Picchioni [4], Ranjita K. Bose [4], Virgilio Mattoli [3] and Andrea Pucci [2,*]

1 Facultad de Ciencias Físicas y Matemáticas, Departamento de Ingeniería Química, Biotecnología y Materiales, Universidad de Chile, Beauchef 851, Santiago 8370456, Chile
2 Dipartimento di Chimica e Chimica Industriale, Università di Pisa, Via Moruzzi 13, 56124 Pisa, Italy; marco.carlotti@unipi.it
3 Center for Materials Interfaces, Istituto Italiano di Tecnologia Viale Rinaldo Piaggio 34, 56025 Pontedera, PI, Italy; virgilio.mattoli@iit.it
4 Department of Chemical Product Engineering, ENTEG, University of Groningen, Nijenborgh 4, 9747 AG Groningen, The Netherlands; f.orozco.gutierrez@rug.nl (F.O.); g.de.macedo.rooweder.lima@rug.nl (G.M.R.L.); f.picchioni@rug.nl (F.P.); r.k.bose@rug.nl (R.K.B.)
5 Instituto Universitario de Investigación y Desarrollo Tecnológico (IDT), Universidad Tecnológica Metropolitana, Ignacio Valdivieso 2409, San Joaquín, Santiago 8940577, Chile; rodrigo.araya@utem.cl (R.A.-H.); dcortes@utem.cl (D.C.-A.)
6 Centro Integrativo de Biología y Química Aplicada (CIBQA), Facultad de Salud, Universidad Bernardo O'Higgins, General Gana 1702, Santiago 8370854, Chile; daniela.ortega@ubo.cl
* Correspondence: earayahermosilla@ing.uchile.cl (E.A.-H.); andrea.pucci@unipi.it (A.P.); Tel.: +39-050-2219270 (A.P.)

Table S1. Composition of polyketones functionalized with FU. The molar ratio between the reactants (primary amine and di-carbonyl groups) in the feed is represented by numerical codes indicating the respective percentages.

Sample	PK (g)	Ratio $NH_2/C{=}O$	Di-carbonyl groups (mmol)	Primary amine group (mmol)	FU (g)	Wt.% (N)
PK0FU20	30.4	0.2	43.4	43.4	4.22	1.79
PK0FU40	38	0.4	108.6	108.6	10.54	3.31
PK0FU60	35.6	0.6	152.6	152.6	14.94	4.06
PK0FU80	32.4	0.8	185.1	185.1	17.98	4.87

The PK was preheated to reach the liquid state at 110 °C. Afterward, FU was added dropwise to the reactor during the first 30 min. The stirring speed was set at a constant value of 400 rpm, and the employed reaction time was 4 h from the moment that the last drop was added. During the reaction, the color of the reaction mixtures changed progressively from light yellow to a range of brown. The resulting products were then allowed to cool down to room temperature before being dissolved with chloroform for the further product purification by solvent extraction technique with brine as a co-solvent. The process was repeated three times to remove any remaining amine compound. The resulting polymer solutions were poured in a round bottom flask for solvent evaporation in a rotavapor, in sequence the product was transferred to a Teflon Petry dish and placed in a vacuum oven at 50 °C for 24 h to remove the solvent completely. To avoid hydration, the samples were sealed in brown glass vials and stored at 6 °C for further characterization.

The percentage of carbonyl conversion (C_{co}) into pyrrole groups was calculated as follows:

$$C_{co} = \frac{y}{y + x} \times 100\% \qquad (S1)$$

Where x and y represent the moles of di-ketone and pyrrolic units after conversion, respectively. Y can be calculated as follows:

$$y = \frac{wt(N)}{A_m(N)} \qquad (S2)$$

Where wt(N) represents the grams of nitrogen in the final product according to the elemental analysis and $A_m(N)$ is the atomic mass of nitrogen. X can be calculated as follows:

$$x = \frac{g_{prod} - y \times M_w^y}{M_w^{pk}} \qquad (S3)$$

Where g_{prod} represents the grams of the product after conversion, M_w^y the molecular weight of the pyrrolic unit and M_w^{pk} the molecular weight of a 1,4 di-ketone unit (140 g/mol). The conversion efficiency η is defined as the ratio between the carbonyl conversion C_{co} and the targeted one according to the amount of polymer and amine compounds provided in the feed (C_{co}^{feed})

$$\eta = \frac{C_{co}}{C_{co}^{feed}} \times 100\% \qquad (S4)$$

The C_{co}^{feed} is calculated as follows:

$$C_{co}^{feed} = \frac{Mol_{amine}}{Mol_{d-co}} \times 100\% \qquad (S5)$$

With Mol_{amine} representing the moles of amine compounds and Mol_{d-co} the moles of di-carbonyl units in the feed.

Table S2. GPC measurements of PK alone and chemically modified with FU at different di-carbonyl conversions.

Sample	*Mn (x10³ M)*	*Mw (x10³ M)*	*PDI*
PK0	3104	5192	1.67
PK0FU20	2743	5671	2.07
PK0FU40	2684	4935	1.84
PK0FU50	2696	5068	1.88
PK0FU60	2537	4910	1.93

GPC analysis was performed on polyketone (PK) and its functionalized derivatives to determine the number average molecular weight (Mn), weight average molecular weight (Mw), and polydispersity index (PDI), as summarized in Table S2. The results for the pristine PK exhibit characteristic Mn, Mw, and PDI values consistent with its oligomeric nature, as previously reported [1]. While the Paal-Knorr reaction does not alter the degree of polymerization, the observed variations in molecular weights are attributed to changes in the chemical structure of the polymer.

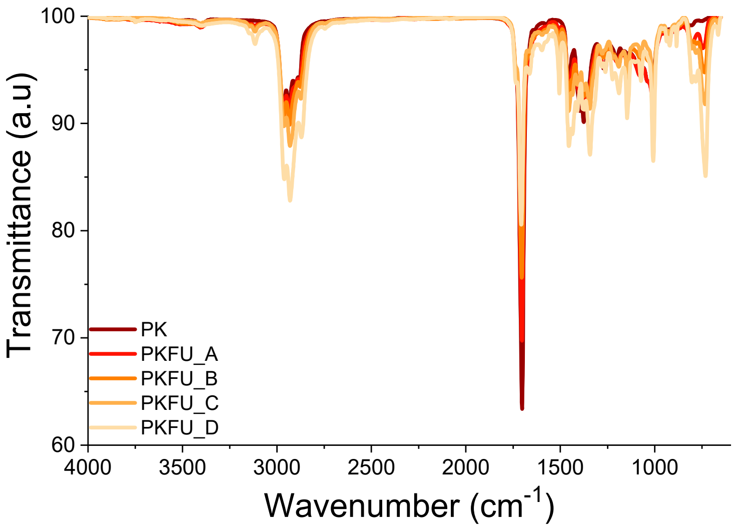

Figure S1. ATR-FT-IR spectrum of PK chemically modified with FU at different CO %. PKFU_A, PKFU_B, PKFU_C, PKFU_D. A=20, B=40. C=60, D=80.

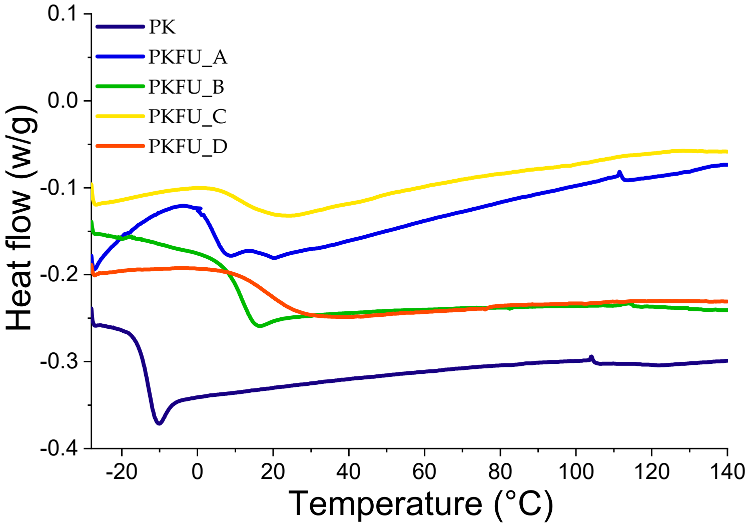

Figure S2. DSC first cycle after thermal history erase of PK0 (PK) grafted with FU at different CO %. PKFU_A, PKFU_B, PKFU_C, PKFU_D. A=20, B=40. C=60, D=80.

.

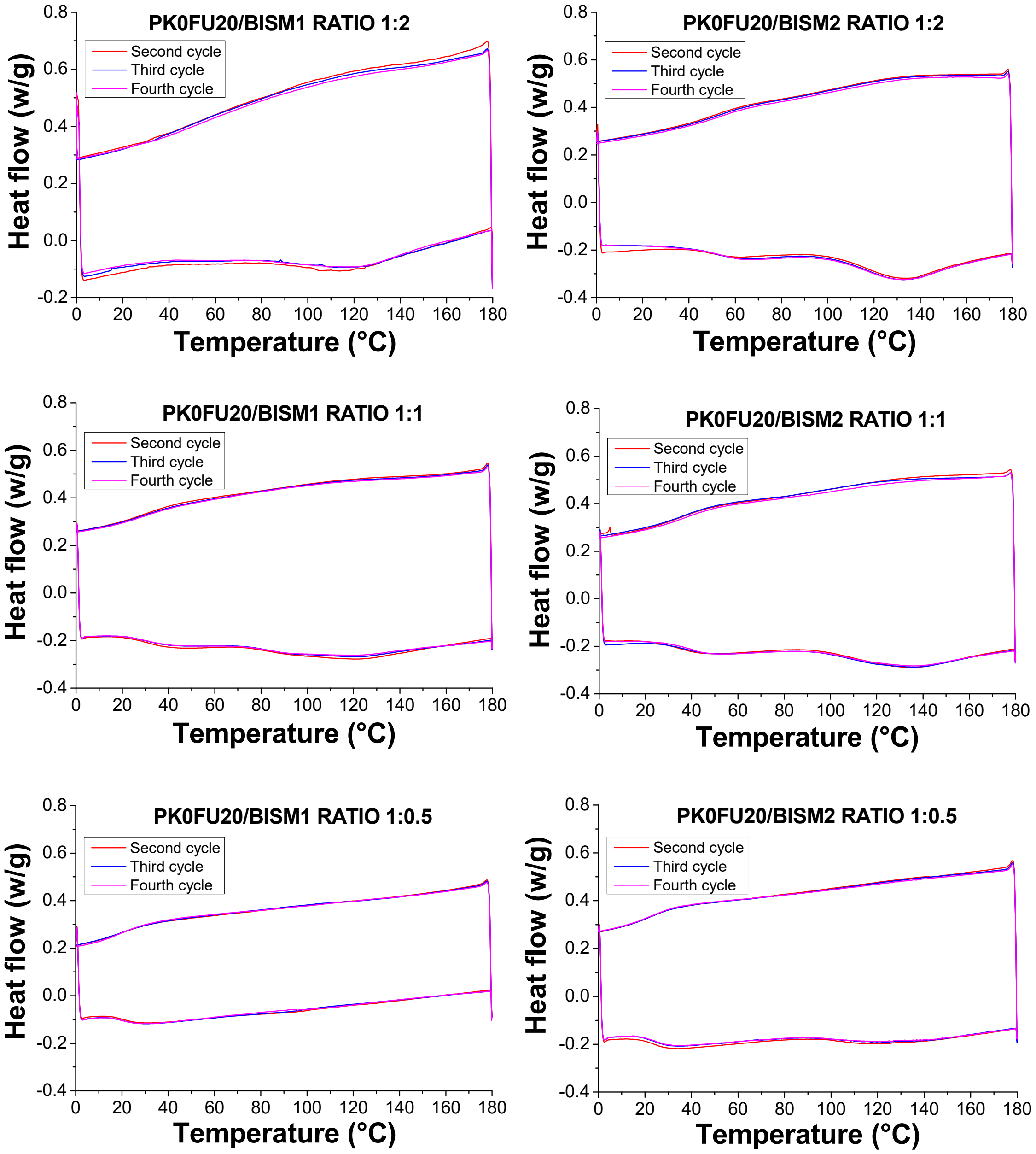

Figure S3. DSC thermal cycles of PK0FU20 series crosslinked with BISM1 or BISM2 at different molar ratios.

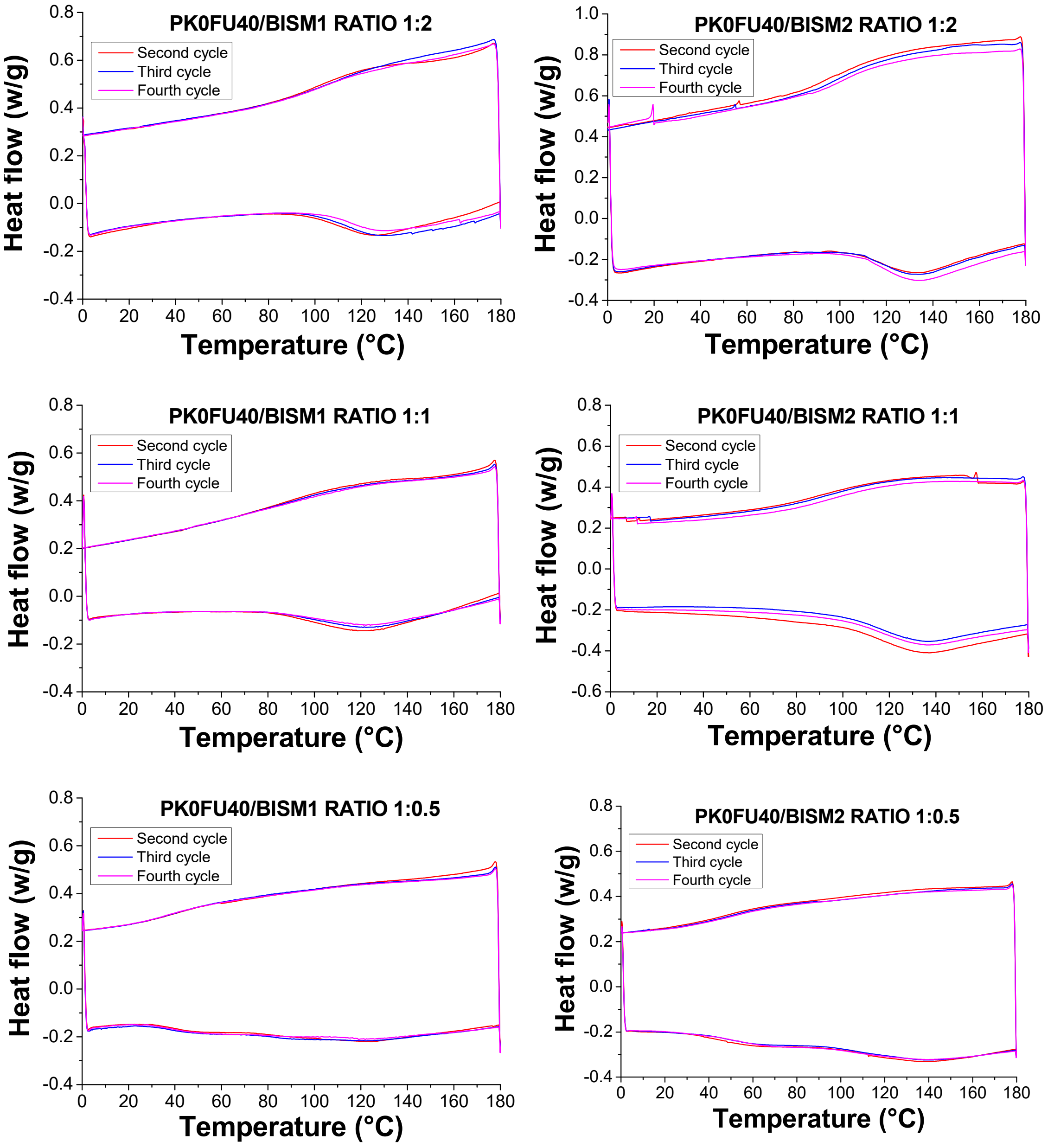

Figure S4. DSC thermal cycles of PK0FU40 series cross-linked with BISM1 or BISM2 at different molar ratios.

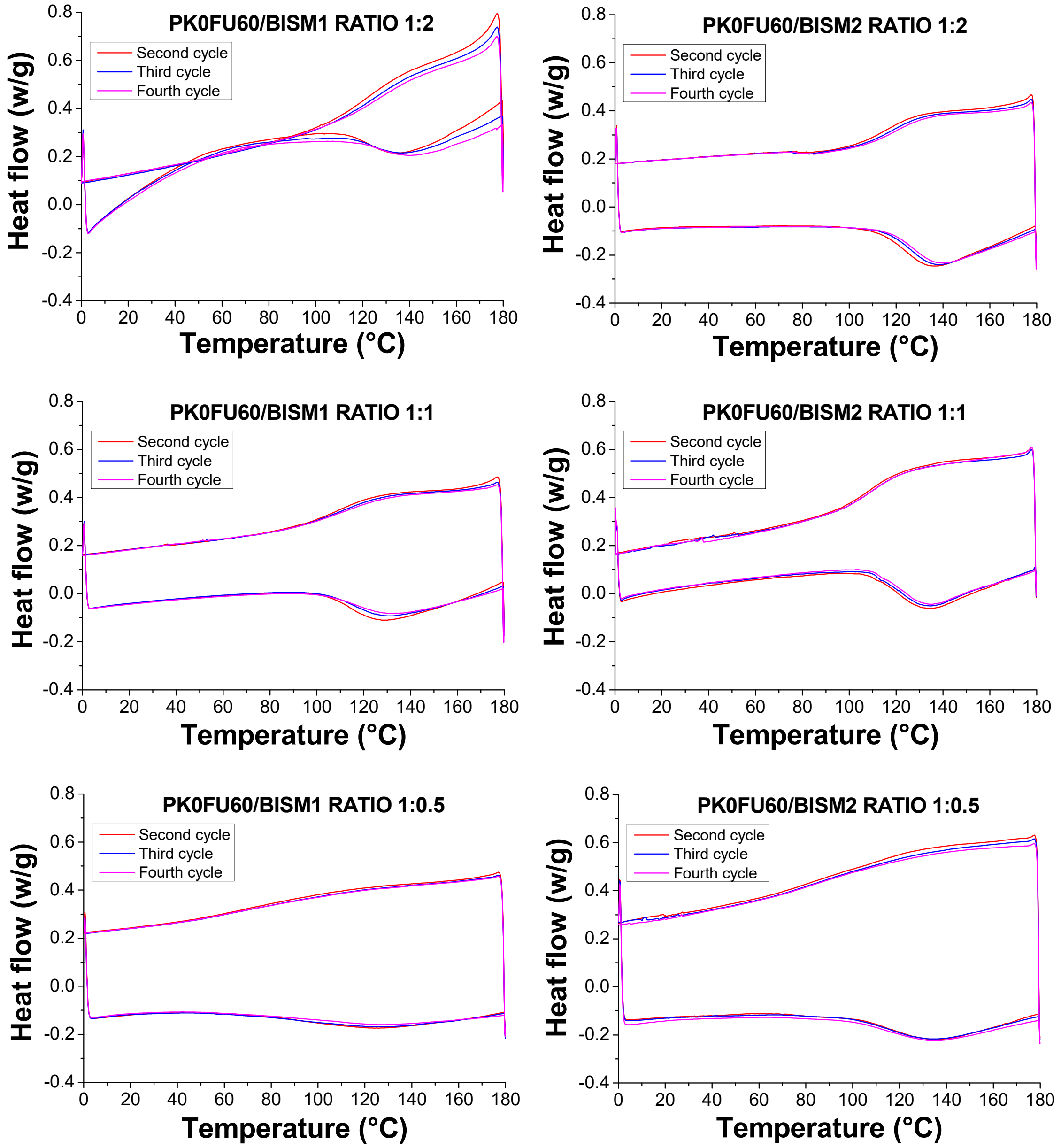

Figure S5. DSC thermal cycles of PK0FU60 series cross-linked with BISM1 or BISM2 at different molar ratios.

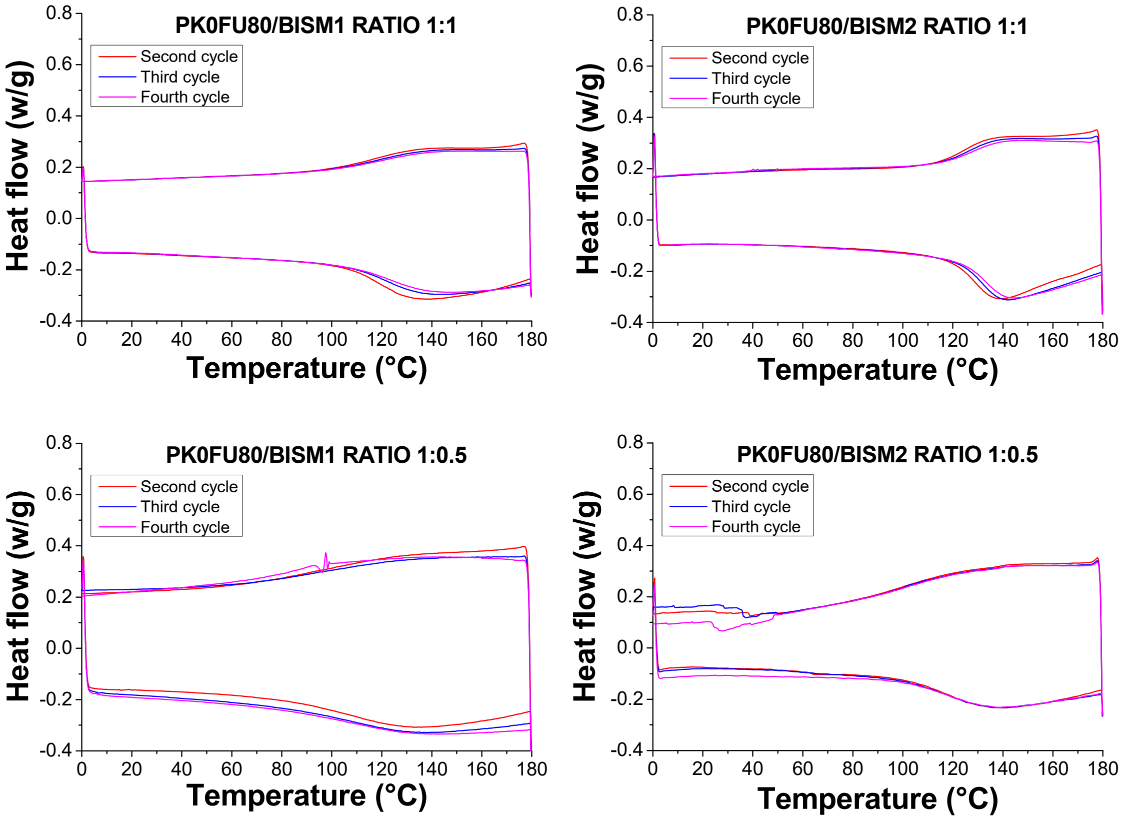

Figure S6. DSC thermal cycles of PK0FU80 series cross-linked with BISM1 or BISM2 at different molar ratios.

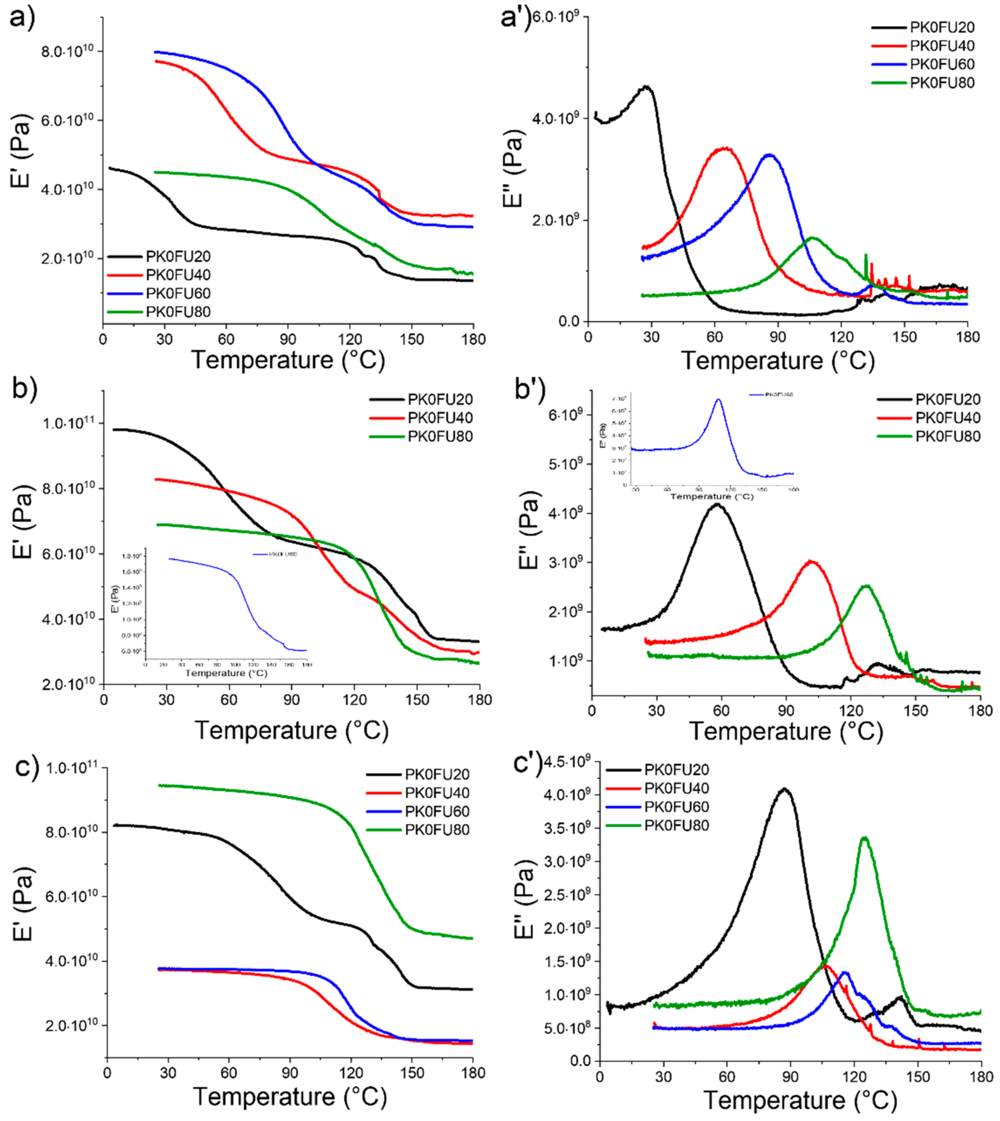

Figure S7. Modulus E′ and E′′ of PK0FU20, PK0FU40, PK0FU60, PK0FU80 crosslinked at different molar ratios with BISM2 (a, a′) 1:0.5, (b, b′) 1:1, (c, c′) 1:2

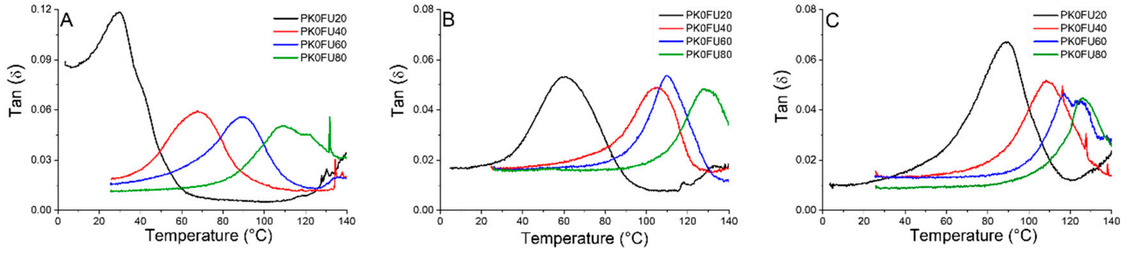

Figure S8. Tan (δ) of PK0FU20, PK0FU40, PK0FU60, PK0FU80 crosslinked at the different molar ratios with BISM2 (A) 1:0.5, (B) 1:1, (C) 1:2

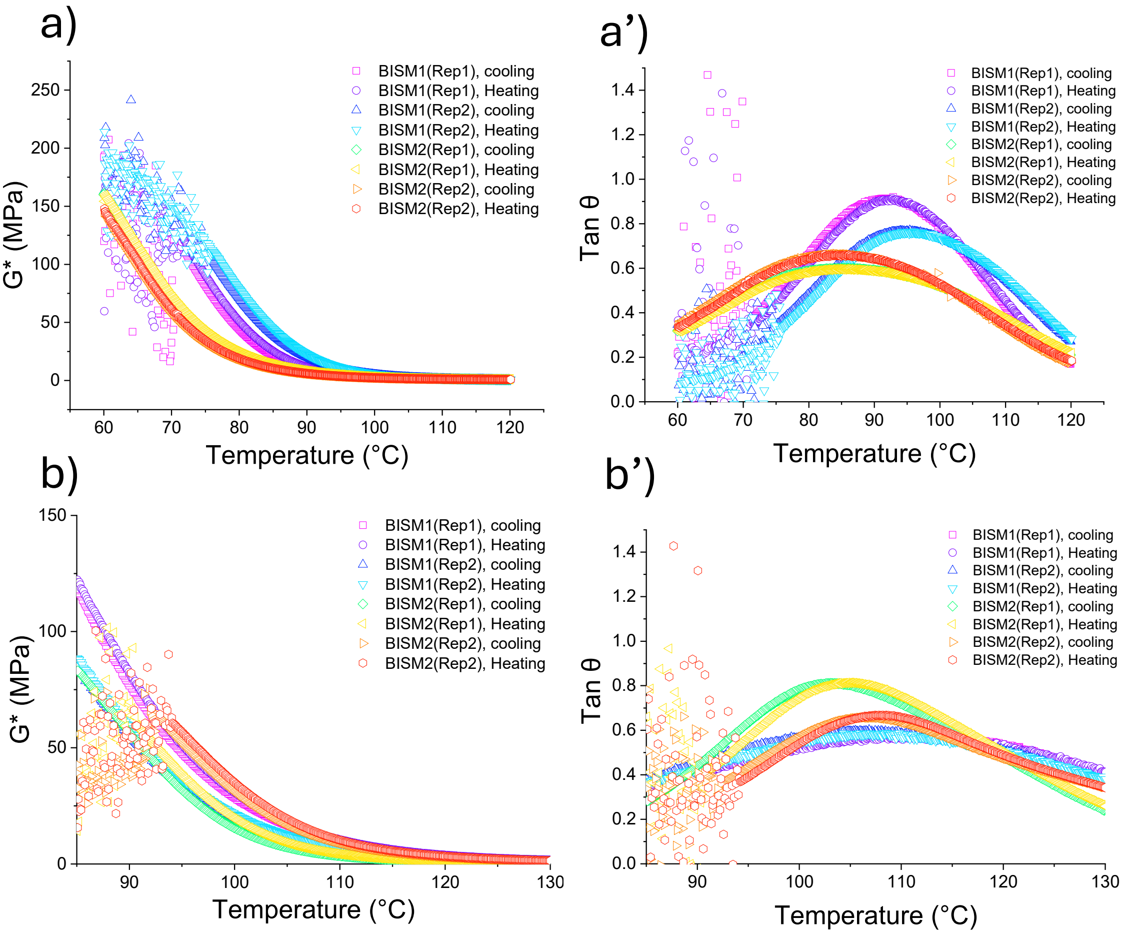

Figure S9. a) Clomplex modulus and a′) Tan δ of PK0FU40 crosslinked with BISM1 and BISM2 at a molar ratio of 1:0.5. b) Clomplex modulus and b′) Tan δ of PK0FU60 crosslinked with BISM1 and BISM2 at a molar ratio of 1:0.5.

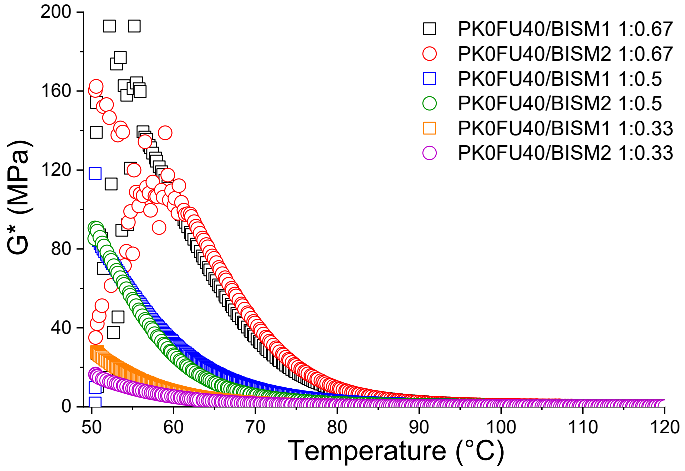

Figure S10. Thermomechanical properties (complex modulus G*) PK0FU40 crosslinked with BISM1 and BISM2 at different molar ratios 1:0.67, 1:0.5, and 1:0.33 FU: BISM determined by rheology

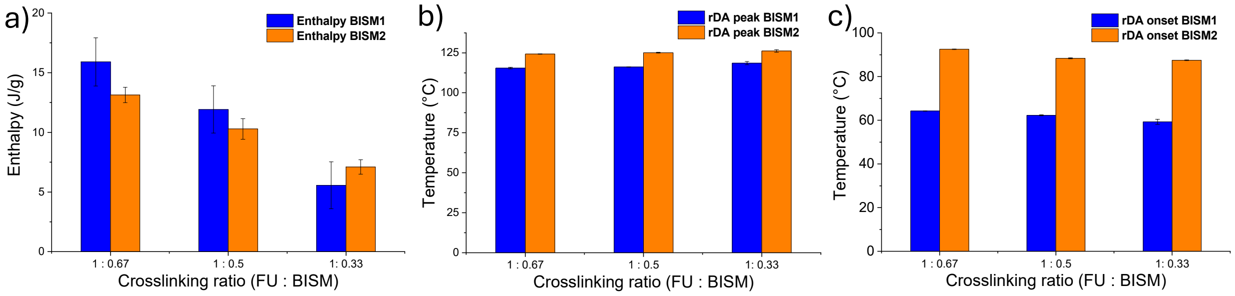

Figure S11. a) Energy required for the retro-Diels-Alder (rDA), b) maximum temperature energy required for the retro-Diels-Alder (rDA), c) onset of the temperature required for the retro-Diels-Alder (rDA) for the thermoset composed by PKFU_B crosslinked with BISM1 and BISM2 at different molar ratios 1:0.67, 1:0.5, and 1:0.33 FU:BISM determined by DSC.

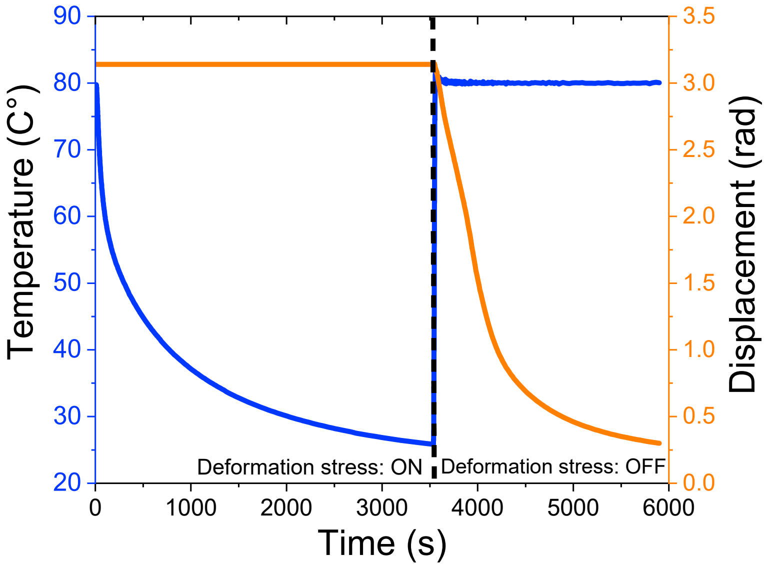

Figure S12. Shape memory analysis of thermoset composed of PK0FU40 and BISM2 at a 1:0.5 (FU:BISM).

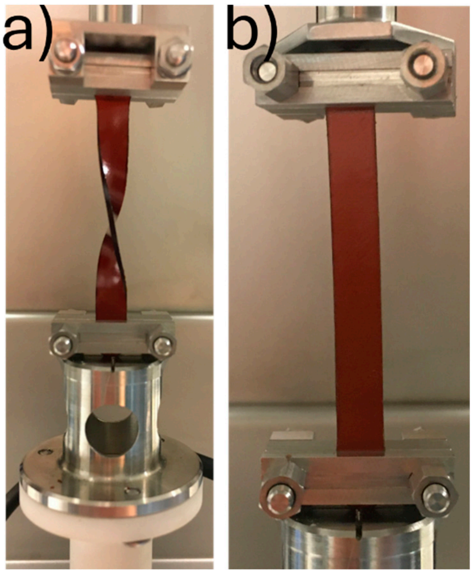

Figure S13. Shape memory analysis of thermoset composed of PK0FU40 and BISM2 at a 1:0.5 (FU:BISM). a) deformation process at 80°C and b) shape recovery after heating at 80°C.

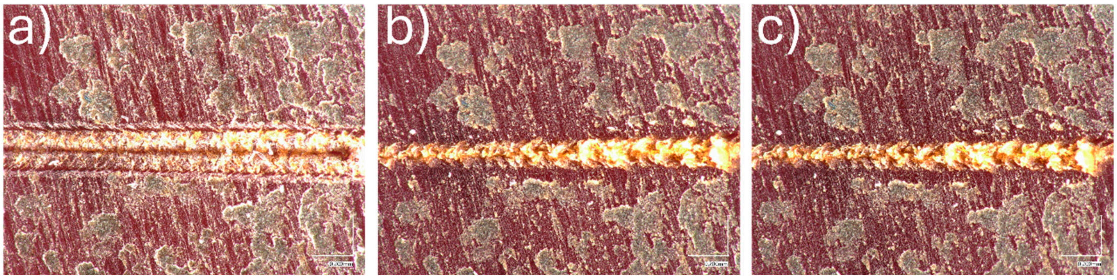

Figure S14. Self-healing test of thermoset PK0FU40 and BISM2 at a 1:0.5 (FU:BISM) crosslinking molar ratio after a) scratching, b) thermal treatment at 100 °C for 5 min, and c) thermal treatment at 100 °C for 20 min.

Table S3. Cartesian coordinates of the optimized structures

Diene

C	-3.149064000000	-1.876616000000	0.406216000000
C	-4.208164000000	-2.548752000000	0.942957000000
C	-3.816322000000	-2.949120000000	2.263264000000
C	-2.547773000000	-2.490695000000	2.433108000000
O	-2.123229000000	-1.839705000000	1.311033000000
H	-5.154712000000	-2.736136000000	0.456367000000
H	-4.403640000000	-3.498952000000	2.984120000000
H	-1.840342000000	-2.534515000000	3.246111000000
C	-2.908832000000	-1.257363000000	-0.935782000000
H	-3.776388000000	-1.483749000000	-1.563854000000
H	-2.045108000000	-1.729709000000	-1.410735000000
N	-2.676344000000	0.183877000000	-0.929643000000
C	-1.457592000000	0.817734000000	-1.166225000000
C	-3.669741000000	1.141163000000	-0.744136000000
C	-1.691328000000	2.175493000000	-1.116194000000
C	-3.072070000000	2.379215000000	-0.851247000000

H	-0.940329000000	2.940048000000	-1.264398000000
H	-3.577271000000	3.329910000000	-0.759980000000
C	-5.107592000000	0.773242000000	-0.513045000000
H	-5.471211000000	0.132634000000	-1.332850000000
H	-5.204120000000	0.164740000000	0.397022000000
C	-6.038694000000	1.987389000000	-0.399506000000
H	-5.695494000000	2.626345000000	0.423141000000
H	-5.959082000000	2.590413000000	-1.312043000000
C	-7.498956000000	1.590391000000	-0.167846000000
H	-7.612582000000	1.012208000000	0.756230000000
H	-8.142276000000	2.472383000000	-0.088223000000
H	-7.880238000000	0.975541000000	-0.991311000000
C	-0.156414000000	0.107887000000	-1.397447000000
H	-0.288025000000	-0.806420000000	-1.988749000000
H	0.465464000000	0.762538000000	-2.018425000000
C	0.622743000000	-0.256265000000	-0.100049000000
H	0.777088000000	0.659531000000	0.483208000000
H	-0.006243000000	-0.901437000000	0.518207000000
C	1.917081000000	-0.969387000000	-0.349870000000
C	2.189349000000	-2.320616000000	-0.343473000000
N	3.093563000000	-0.317792000000	-0.710689000000
C	3.557752000000	-2.498755000000	-0.692777000000
H	1.471306000000	-3.097233000000	-0.116362000000
C	4.105464000000	-1.251871000000	-0.907769000000
H	4.083568000000	-3.438636000000	-0.781254000000
C	3.290131000000	1.119948000000	-0.699275000000
H	4.028599000000	1.387999000000	-1.462833000000
H	2.357584000000	1.611804000000	-0.994656000000
C	3.735221000000	1.628246000000	0.641370000000
C	3.934951000000	1.040797000000	1.854482000000
O	4.002382000000	2.967519000000	0.715069000000
C	4.355149000000	2.088110000000	2.743266000000
H	3.797170000000	-0.005729000000	2.082021000000
C	4.378091000000	3.228452000000	2.004679000000
H	4.604557000000	1.995977000000	3.790431000000
H	4.623566000000	4.255920000000	2.222565000000
C	5.501955000000	-0.845495000000	-1.282536000000
H	5.490573000000	-0.282596000000	-2.228297000000
H	5.905032000000	-0.152498000000	-0.530351000000
C	6.456818000000	-2.034077000000	-1.429132000000
H	6.533192000000	-2.595171000000	-0.492730000000
H	6.113329000000	-2.723605000000	-2.206394000000
H	7.459300000000	-1.691035000000	-1.700676000000

BISM1

C	-5.617437000000	0.831224000000	-1.166086000000
C	-6.790603000000	1.633485000000	-0.687607000000
C	-6.718061000000	1.757506000000	0.639029000000
C	-5.494210000000	1.042201000000	1.128357000000
H	-7.532695000000	2.013267000000	-1.377098000000
H	-7.383468000000	2.268214000000	1.322219000000
N	-4.868241000000	0.502239000000	-0.015800000000
O	-5.355974000000	0.523909000000	-2.308860000000
O	-5.113264000000	0.940357000000	2.274405000000

C	-3.660802000000	-0.259484000000	-0.010816000000
C	-2.706549000000	-0.067099000000	-1.017647000000
C	-3.426628000000	-1.195310000000	0.999504000000
C	-1.534678000000	-0.816107000000	-1.009297000000
H	-2.889097000000	0.652164000000	-1.805848000000
C	-2.238840000000	-1.926055000000	0.999494000000
H	-4.156190000000	-1.339096000000	1.786202000000
C	-1.278081000000	-1.756726000000	-0.002160000000
H	-0.799624000000	-0.656653000000	-1.793194000000
H	-2.065011000000	-2.648483000000	1.792655000000
C	0.000008000000	-2.578419000000	-0.010958000000
H	-0.003475000000	-3.242376000000	0.862254000000
H	0.003417000000	-3.234922000000	-0.889795000000
C	1.278138000000	-1.756784000000	-0.012754000000
C	2.238370000000	-1.916744000000	-1.016448000000
C	1.535249000000	-0.825589000000	1.002982000000
C	3.426148000000	-1.185998000000	-1.010264000000
H	2.064135000000	-2.631752000000	-1.816221000000
C	2.707097000000	-0.076647000000	1.017690000000
H	0.800616000000	-0.673497000000	1.788741000000
C	3.660809000000	-0.259628000000	0.008604000000
H	4.155330000000	-1.322439000000	-1.798627000000
H	2.890050000000	0.635264000000	1.812450000000
N	4.868239000000	0.502057000000	0.020058000000
C	5.618018000000	0.820309000000	1.172967000000
C	5.493595000000	1.052675000000	-1.119336000000
C	6.790891000000	1.627079000000	0.701390000000
C	6.717637000000	1.763486000000	-0.623991000000
H	7.533302000000	2.000423000000	1.394045000000
H	7.382605000000	2.280618000000	-1.302761000000
O	5.357114000000	0.502409000000	2.312962000000
O	5.112085000000	0.961442000000	-2.266099000000

BISM2

C	6.205658000000	-0.509813000000	0.067384000000
C	7.160994000000	-1.609553000000	-0.290830000000
C	6.464526000000	-2.710060000000	-0.581196000000
C	5.001751000000	-2.412743000000	-0.432277000000
H	8.231044000000	-1.450209000000	-0.287843000000
H	6.813478000000	-3.688878000000	-0.882100000000
N	4.911763000000	-1.061371000000	-0.036419000000
O	6.483456000000	0.627908000000	0.381209000000
O	4.076774000000	-3.174903000000	-0.612380000000
C	3.693344000000	-0.360547000000	0.220196000000
C	3.540882000000	0.955584000000	-0.212055000000
C	2.662129000000	-0.983214000000	0.916328000000
C	2.358104000000	1.657158000000	0.035710000000
H	4.361956000000	1.436269000000	-0.723574000000
C	1.459797000000	-0.312336000000	1.162329000000
H	2.785798000000	-2.000601000000	1.266713000000
C	1.285376000000	1.012301000000	0.702788000000
C	-0.006275000000	1.783441000000	0.988953000000
H	0.005380000000	2.053154000000	2.049207000000
H	0.031178000000	2.738654000000	0.458465000000
C	-1.355993000000	1.141702000000	0.665573000000

C	-1.651410000000	0.667952000000	-0.636200000000
C	-2.359633000000	1.092921000000	1.659595000000
C	-2.900726000000	0.097594000000	-0.895729000000
C	-3.609827000000	0.540489000000	1.365029000000
C	-3.875609000000	0.029093000000	0.098302000000
H	-3.132967000000	-0.272662000000	-1.883818000000
H	-4.372112000000	0.499009000000	2.133156000000
N	-5.149731000000	-0.550745000000	-0.187799000000
C	-5.880889000000	-0.362246000000	-1.379329000000
C	-5.867511000000	-1.399997000000	0.680560000000
C	-7.142926000000	-1.158925000000	-1.227987000000
C	-7.134592000000	-1.760369000000	-0.036903000000
H	-7.890151000000	-1.185506000000	-2.009876000000
H	-7.871620000000	-2.412287000000	0.412743000000
O	-5.549137000000	0.309789000000	-2.332241000000
O	-5.523359000000	-1.759518000000	1.785802000000
C	0.398850000000	-1.050859000000	1.948547000000
H	0.022595000000	-0.453611000000	2.784736000000
H	-0.465148000000	-1.314212000000	1.331941000000
H	0.808716000000	-1.975689000000	2.362456000000
C	-2.153805000000	1.648549000000	3.054566000000
H	-1.342523000000	1.144859000000	3.592198000000
H	-1.913716000000	2.717741000000	3.040159000000
H	-3.061332000000	1.525415000000	3.650460000000
C	2.252160000000	3.113866000000	-0.406290000000
H	1.402545000000	3.224233000000	-1.093381000000
H	1.999127000000	3.729059000000	0.467218000000
C	3.489604000000	3.719928000000	-1.075089000000
H	4.364877000000	3.686680000000	-0.419029000000
H	3.745473000000	3.202837000000	-2.005496000000
H	3.299514000000	4.768288000000	-1.323853000000
C	-0.633275000000	0.788257000000	-1.761407000000
H	-0.263054000000	1.821709000000	-1.777813000000
H	0.243235000000	0.175706000000	-1.523840000000
C	-1.121862000000	0.428553000000	-3.167643000000
H	-1.395383000000	-0.628529000000	-3.247376000000
H	-1.991115000000	1.024187000000	-3.464204000000
H	-0.324934000000	0.612635000000	-3.894389000000

$R1_{endo\text{-}BISM1}$

C	0.242363000000	1.554664000000	2.371393000000
C	0.558933000000	1.703795000000	1.057617000000
C	-0.624865000000	2.197403000000	0.410786000000
C	-1.572386000000	2.312369000000	1.384229000000
O	-1.058695000000	1.922524000000	2.590434000000
H	0.789634000000	1.211457000000	3.235006000000
H	1.512160000000	1.484580000000	0.599209000000
H	-0.757344000000	2.437284000000	-0.633685000000
C	-3.007848000000	2.753617000000	1.402861000000
H	-3.137787000000	3.547337000000	2.145073000000
H	-3.644543000000	1.924627000000	1.733577000000
C	0.626809000000	-1.826846000000	2.169640000000
C	-0.811335000000	-1.896527000000	2.581977000000
C	-1.571168000000	-2.034327000000	1.493117000000
C	-0.682234000000	-2.052139000000	0.286644000000

H	-1.103163000000	-1.836988000000	3.621889000000
H	-2.646370000000	-2.119056000000	1.396859000000
N	0.641210000000	-1.947106000000	0.762740000000
O	1.594644000000	-1.695907000000	2.888943000000
O	-1.021280000000	-2.134559000000	-0.876177000000
C	1.811202000000	-1.901008000000	-0.051409000000
C	2.977483000000	-2.556358000000	0.363154000000
C	1.799829000000	-1.203798000000	-1.262730000000
C	4.119239000000	-2.502472000000	-0.429593000000
H	2.991636000000	-3.089179000000	1.305465000000
C	2.949300000000	-1.175702000000	-2.052296000000
H	0.897939000000	-0.703518000000	-1.590996000000
C	4.125080000000	-1.819129000000	-1.653503000000
H	5.022417000000	-3.002128000000	-0.090806000000
H	2.925952000000	-0.639144000000	-2.997262000000
N	-3.476151000000	3.233337000000	0.115341000000
C	-3.880716000000	2.415191000000	-0.936914000000
C	-3.513404000000	4.566369000000	-0.289688000000
C	-4.175674000000	3.236768000000	-2.003703000000
C	-3.950357000000	4.579829000000	-1.598137000000
H	-4.529718000000	2.898335000000	-2.968147000000
H	-4.099634000000	5.472034000000	-2.190900000000
C	-3.143603000000	5.721736000000	0.594786000000
H	-3.576860000000	5.591310000000	1.596148000000
H	-3.630624000000	6.611664000000	0.178659000000
C	-1.630291000000	6.010305000000	0.737549000000
H	-1.503667000000	6.805258000000	1.483713000000
H	-1.122922000000	5.125243000000	1.138287000000
C	-0.963565000000	6.428396000000	-0.574947000000
H	-1.407362000000	7.351099000000	-0.967159000000
H	0.107126000000	6.607602000000	-0.434048000000
H	-1.080804000000	5.655395000000	-1.340538000000
C	-4.014790000000	0.925834000000	-0.820058000000
H	-3.928415000000	0.500022000000	-1.824674000000
H	-3.185047000000	0.501118000000	-0.243244000000
C	-5.357728000000	0.470426000000	-0.207847000000
H	-5.467151000000	0.921410000000	0.790370000000
H	-6.171610000000	0.896790000000	-0.809754000000
C	-5.491099000000	-1.022967000000	-0.121740000000
C	-4.632719000000	-2.032720000000	-0.507005000000
N	-6.626024000000	-1.628281000000	0.408997000000
C	-5.262310000000	-3.277608000000	-0.208657000000
H	-3.658320000000	-1.904290000000	-0.957606000000
C	-6.493298000000	-3.012139000000	0.349867000000
H	-4.851833000000	-4.260959000000	-0.387897000000
C	-7.568956000000	-3.936787000000	0.843892000000
H	-7.754639000000	-3.764094000000	1.915033000000
H	-8.518010000000	-3.708852000000	0.338459000000
C	-7.233873000000	-5.416731000000	0.634958000000
H	-6.319209000000	-5.694467000000	1.167702000000
H	-8.045148000000	-6.050429000000	1.004383000000
H	-7.085176000000	-5.640340000000	-0.425767000000
C	-7.822951000000	-0.927172000000	0.834365000000
H	-7.539033000000	0.039007000000	1.265524000000
H	-8.300875000000	-1.491427000000	1.642757000000
C	-8.796048000000	-0.718826000000	-0.289865000000

C	-8.793018000000	-1.042422000000	-1.613536000000
O	-9.952603000000	-0.065626000000	0.034655000000
C	-10.034376000000	-0.555793000000	-2.148220000000
H	-8.004715000000	-1.561149000000	-2.138499000000
C	-10.694413000000	0.024238000000	-1.111885000000
H	-10.380447000000	-0.631220000000	-3.168851000000
H	-11.646695000000	0.521590000000	-1.016100000000
C	5.365173000000	-1.800226000000	-2.531751000000
H	5.551693000000	-2.810500000000	-2.916890000000
H	5.162320000000	-1.176068000000	-3.410869000000
C	6.624093000000	-1.303336000000	-1.841938000000
C	7.782053000000	-2.085954000000	-1.800701000000
C	6.663390000000	-0.037174000000	-1.241806000000
C	8.950571000000	-1.625706000000	-1.194504000000
H	7.779197000000	-3.072037000000	-2.257703000000
C	7.814472000000	0.432844000000	-0.618627000000
H	5.774137000000	0.586811000000	-1.249995000000
C	8.966827000000	-0.362798000000	-0.597607000000
H	9.835205000000	-2.249054000000	-1.170454000000
H	7.826638000000	1.414834000000	-0.163263000000
N	10.153544000000	0.114852000000	0.036199000000
C	10.628030000000	1.443110000000	-0.021149000000
C	11.030547000000	-0.668483000000	0.817332000000
C	11.890952000000	1.473116000000	0.786860000000
C	12.122397000000	0.252524000000	1.273801000000
H	12.463315000000	2.383539000000	0.904522000000
H	12.932697000000	-0.099781000000	1.897992000000
O	10.110375000000	2.368961000000	-0.607406000000
O	10.917898000000	-1.850091000000	1.062235000000

R1$_{endo\text{-}BISM2}$

C	0.819462000000	1.908308000000	-2.673989000000
C	0.437939000000	2.062388000000	-1.378226000000
C	1.621225000000	2.426555000000	-0.649881000000
C	2.633817000000	2.467204000000	-1.561771000000
O	2.160711000000	2.152506000000	-2.805884000000
H	0.296123000000	1.635419000000	-3.576590000000
H	-0.557966000000	1.925222000000	-0.983012000000
H	1.711890000000	2.631295000000	0.406477000000
C	4.104089000000	2.762686000000	-1.482385000000
H	4.358514000000	3.554981000000	-2.193210000000
H	4.674878000000	1.880970000000	-1.796751000000
C	0.013742000000	-1.398209000000	-2.494422000000
C	1.449527000000	-1.639112000000	-2.847313000000
C	2.138009000000	-1.881902000000	-1.729843000000
C	1.200139000000	-1.807245000000	-0.562758000000
H	1.792119000000	-1.602399000000	-3.872720000000
H	3.189886000000	-2.097127000000	-1.589685000000
N	-0.077405000000	-1.534835000000	-1.092337000000
O	-0.897091000000	-1.137640000000	-3.252051000000
O	1.476345000000	-1.946843000000	0.611537000000
C	-1.270565000000	-1.356683000000	-0.329935000000
C	-2.477769000000	-1.876343000000	-0.790602000000
C	-1.239615000000	-0.686908000000	0.890186000000
C	-3.666003000000	-1.708272000000	-0.070195000000
H	-2.501212000000	-2.419202000000	-1.727418000000

C	-2.408250000000	-0.512918000000	1.638443000000
H	-0.296896000000	-0.322250000000	1.279549000000
C	-3.650064000000	-0.989647000000	1.150146000000
N	4.535624000000	3.165458000000	-0.156156000000
C	4.784975000000	2.289107000000	0.897440000000
C	4.682604000000	4.479096000000	0.285302000000
C	5.092597000000	3.054519000000	2.001826000000
C	5.032761000000	4.421473000000	1.618406000000
H	5.346756000000	2.662220000000	2.977308000000
H	5.234006000000	5.281546000000	2.242488000000
C	4.492346000000	5.683566000000	-0.590393000000
H	4.980461000000	5.530590000000	-1.562955000000
H	5.034435000000	6.511184000000	-0.117643000000
C	3.029168000000	6.126055000000	-0.828769000000
H	3.035563000000	6.945800000000	-1.558648000000
H	2.466403000000	5.305730000000	-1.288940000000
C	2.314607000000	6.580612000000	0.445948000000
H	2.819457000000	7.443867000000	0.895481000000
H	1.280459000000	6.871858000000	0.235934000000
H	2.297400000000	5.782723000000	1.194624000000
C	4.770985000000	0.796410000000	0.750592000000
H	4.578837000000	0.363099000000	1.737172000000
H	3.939077000000	0.472397000000	0.114615000000
C	6.094150000000	0.213320000000	0.207766000000
H	6.311057000000	0.668838000000	-0.770575000000
H	6.909908000000	0.539532000000	0.867080000000
C	6.075018000000	-1.283889000000	0.091923000000
C	5.096447000000	-2.204151000000	0.408435000000
N	7.166817000000	-1.995745000000	-0.395377000000
C	5.607061000000	-3.502785000000	0.111909000000
H	4.117931000000	-1.982030000000	0.811193000000
C	6.886848000000	-3.358620000000	-0.377185000000
H	5.086908000000	-4.440363000000	0.246084000000
C	7.883991000000	-4.382404000000	-0.839504000000
H	8.141826000000	-4.210310000000	-1.895707000000
H	8.824370000000	-4.265756000000	-0.282518000000
C	7.385511000000	-5.822292000000	-0.683177000000
H	6.475569000000	-5.991699000000	-1.267014000000
H	8.144015000000	-6.531169000000	-1.027268000000
H	7.159576000000	-6.048846000000	0.363188000000
C	8.450958000000	-1.417390000000	-0.743854000000
H	8.292691000000	-0.419229000000	-1.166482000000
H	8.908890000000	-2.014760000000	-1.539950000000
C	9.380433000000	-1.332495000000	0.432192000000
C	9.276531000000	-1.682322000000	1.745066000000
O	10.612113000000	-0.793832000000	0.182357000000
C	10.531336000000	-1.336305000000	2.352861000000
H	8.413909000000	-2.129066000000	2.216639000000
C	11.299049000000	-0.804573000000	1.365964000000
H	10.814901000000	-1.468363000000	3.386931000000
H	12.300229000000	-0.404889000000	1.330345000000
C	-4.893205000000	-0.763694000000	2.026979000000
H	-4.905561000000	-1.549905000000	2.788497000000
H	-4.729807000000	0.162083000000	2.586747000000
C	-6.281535000000	-0.654451000000	1.410821000000
C	-7.271411000000	-1.612601000000	1.718185000000

C	-6.617136000000	0.450754000000	0.592554000000
C	-8.559178000000	-1.478769000000	1.190223000000
C	-7.903380000000	0.547760000000	0.056077000000
C	-8.871893000000	-0.411372000000	0.352669000000
H	-9.313467000000	-2.220786000000	1.420828000000
H	-8.167196000000	1.382323000000	-0.577496000000
N	-10.185417000000	-0.293074000000	-0.196948000000
C	-10.909281000000	0.911359000000	-0.322900000000
C	-10.955611000000	-1.364176000000	-0.697539000000
C	-12.223290000000	0.548234000000	-0.948625000000
C	-12.249516000000	-0.767888000000	-1.166736000000
H	-12.975563000000	1.297720000000	-1.155365000000
H	-13.027583000000	-1.380412000000	-1.602408000000
O	-10.536785000000	2.016317000000	0.008690000000
O	-10.630512000000	-2.531277000000	-0.735039000000
C	-6.997344000000	-2.798545000000	2.620533000000
H	-6.164245000000	-3.412652000000	2.260922000000
H	-6.748057000000	-2.488085000000	3.642077000000
H	-7.876770000000	-3.444112000000	2.681230000000
C	-4.905963000000	-2.351463000000	-0.657858000000
H	-5.435952000000	-2.969855000000	0.070487000000
H	-5.623452000000	-1.614599000000	-1.028817000000
H	-4.627748000000	-2.995061000000	-1.496409000000
C	-2.250791000000	0.197367000000	2.976312000000
H	-2.976230000000	-0.174297000000	3.705917000000
H	-1.266413000000	-0.063619000000	3.380358000000
C	-2.347308000000	1.730911000000	2.886891000000
H	-3.324649000000	2.056728000000	2.518003000000
H	-2.188664000000	2.188921000000	3.868673000000
H	-1.590308000000	2.127802000000	2.203009000000
C	-5.588021000000	1.534160000000	0.306980000000
H	-5.279139000000	1.972645000000	1.266648000000
H	-4.681656000000	1.069362000000	-0.097354000000
C	-6.024126000000	2.668062000000	-0.624417000000
H	-6.289083000000	2.295971000000	-1.619566000000
H	-6.885249000000	3.215874000000	-0.228821000000
H	-5.205172000000	3.382805000000	-0.748430000000

$TS1_{endo\text{-}BISM1}$

C	-5.254178000000	-3.182378000000	0.449234000000
C	-4.336108000000	-2.640636000000	1.396596000000
C	-3.881895000000	-1.467892000000	0.848123000000
C	-4.556576000000	-1.311970000000	-0.398848000000
O	-5.639909000000	-2.164943000000	-0.395126000000
H	-6.004154000000	-3.942387000000	0.622896000000
H	-3.973484000000	-3.149814000000	2.278395000000
H	-3.092608000000	-0.829356000000	1.216154000000
C	-4.609030000000	-0.095840000000	-1.290566000000
H	-5.299015000000	-0.302542000000	-2.112036000000
H	-3.616639000000	0.026579000000	-1.730241000000
C	-2.745182000000	-4.505721000000	-0.229956000000
C	-3.944476000000	-3.968331000000	-0.951604000000
C	-3.536202000000	-2.795370000000	-1.617658000000
C	-2.103171000000	-2.566890000000	-1.344576000000
H	-4.649693000000	-4.685092000000	-1.354735000000
H	-3.952826000000	-2.433440000000	-2.548487000000

N	-1.695541000000	-3.576439000000	-0.436058000000
O	-2.666025000000	-5.528262000000	0.420241000000
O	-1.394228000000	-1.679608000000	-1.784651000000
C	-0.395048000000	-3.656484000000	0.144444000000
C	0.232699000000	-4.894533000000	0.313645000000
C	0.268760000000	-2.484487000000	0.530741000000
C	1.514233000000	-4.948333000000	0.860986000000
H	-0.278497000000	-5.803577000000	0.025878000000
C	1.549842000000	-2.557869000000	1.066458000000
H	-0.198566000000	-1.519450000000	0.383617000000
C	2.197203000000	-3.788464000000	1.240962000000
H	1.993045000000	-5.916551000000	0.983645000000
H	2.059396000000	-1.638338000000	1.338662000000
N	-4.981368000000	1.154238000000	-0.643989000000
C	-4.070179000000	2.064666000000	-0.108645000000
C	-6.278125000000	1.645997000000	-0.488317000000
C	-4.798137000000	3.127079000000	0.383278000000
C	-6.172801000000	2.869997000000	0.140164000000
H	-4.372522000000	3.999045000000	0.861558000000
H	-7.007916000000	3.510241000000	0.389645000000
C	-7.517662000000	0.923454000000	-0.933065000000
H	-7.307791000000	0.302491000000	-1.812638000000
H	-8.236910000000	1.679497000000	-1.271775000000
C	-8.204565000000	0.043252000000	0.139732000000
H	-9.016191000000	-0.511543000000	-0.349053000000
H	-7.493418000000	-0.706655000000	0.500711000000
C	-8.769461000000	0.835045000000	1.321076000000
H	-9.525569000000	1.556738000000	0.989468000000
H	-9.244192000000	0.169085000000	2.049177000000
H	-7.981498000000	1.392198000000	1.836337000000
C	-2.578258000000	1.901837000000	-0.138787000000
H	-2.172123000000	2.507104000000	0.677165000000
H	-2.269221000000	0.871408000000	0.070615000000
C	-1.879032000000	2.335771000000	-1.461201000000
H	-2.176726000000	1.661271000000	-2.271292000000
H	-2.256802000000	3.325085000000	-1.743873000000
C	-0.381277000000	2.316041000000	-1.374521000000
C	0.487933000000	1.270096000000	-1.604469000000
N	0.386835000000	3.409972000000	-0.975530000000
C	1.809365000000	1.729080000000	-1.341507000000
H	0.193584000000	0.281225000000	-1.928813000000
C	1.731816000000	3.051030000000	-0.956928000000
H	2.722494000000	1.159511000000	-1.444178000000
C	2.811278000000	4.034625000000	-0.603447000000
H	2.854174000000	4.836661000000	-1.356959000000
H	2.568614000000	4.533006000000	0.345464000000
C	4.197411000000	3.392386000000	-0.489431000000
H	4.502187000000	2.922421000000	-1.428375000000
H	4.946283000000	4.146173000000	-0.226532000000
H	4.206998000000	2.618758000000	0.284689000000
C	-0.116523000000	4.713249000000	-0.588148000000
H	-1.063143000000	4.904075000000	-1.102997000000
H	0.578711000000	5.484097000000	-0.941188000000
C	-0.315297000000	4.850080000000	0.894616000000
C	-0.025921000000	4.060874000000	1.968101000000
O	-0.895368000000	6.016195000000	1.311409000000

C	-0.457976000000	4.786971000000	3.130201000000
H	0.439491000000	3.087216000000	1.930402000000
C	-0.973922000000	5.959120000000	2.676513000000
H	-0.391054000000	4.470685000000	4.161076000000
H	-1.417418000000	6.816998000000	3.156869000000
C	3.592352000000	-3.862044000000	1.839302000000
H	3.977438000000	-4.881209000000	1.705919000000
H	3.533839000000	-3.706295000000	2.924398000000
C	4.584261000000	-2.867891000000	1.260224000000
C	4.756680000000	-2.746752000000	-0.125826000000
C	5.359180000000	-2.054526000000	2.093379000000
C	5.671026000000	-1.847356000000	-0.663955000000
H	4.160140000000	-3.359581000000	-0.795087000000
C	6.270857000000	-1.137632000000	1.571270000000
H	5.244622000000	-2.128552000000	3.171631000000
C	6.426398000000	-1.033045000000	0.187425000000
H	5.786909000000	-1.762257000000	-1.737376000000
H	6.860034000000	-0.514059000000	2.232200000000
N	7.348828000000	-0.092287000000	-0.361598000000
C	8.648896000000	0.154185000000	0.128682000000
C	7.089656000000	0.744085000000	-1.466375000000
C	9.233957000000	1.216416000000	-0.754200000000
C	8.331143000000	1.559249000000	-1.675468000000
H	10.237046000000	1.590461000000	-0.598459000000
H	8.399700000000	2.289439000000	-2.470744000000
O	6.063268000000	0.788538000000	-2.111024000000
O	9.174552000000	-0.386916000000	1.076972000000

$TS1_{endo\text{-}BISM2}$

C	-5.367934000000	-3.311939000000	0.319887000000
C	-4.492653000000	-2.715768000000	1.274710000000
C	-4.162608000000	-1.486503000000	0.762403000000
C	-4.866716000000	-1.355623000000	-0.471143000000
O	-5.863076000000	-2.309457000000	-0.483991000000
H	-6.037277000000	-4.147534000000	0.474693000000
H	-4.069015000000	-3.216707000000	2.133717000000
H	-3.433600000000	-0.786878000000	1.143425000000
C	-5.050188000000	-0.119583000000	-1.317189000000
H	-5.730949000000	-0.362618000000	-2.136427000000
H	-4.081600000000	0.112918000000	-1.765436000000
C	-2.746182000000	-4.355217000000	-0.434721000000
C	-4.004973000000	-3.918191000000	-1.123561000000
C	-3.727551000000	-2.687542000000	-1.752132000000
C	-2.320468000000	-2.324404000000	-1.486861000000
H	-4.641626000000	-4.687947000000	-1.542777000000
H	-4.192605000000	-2.338960000000	-2.665025000000
N	-1.801450000000	-3.316819000000	-0.619546000000
O	-2.552138000000	-5.385868000000	0.177497000000
O	-1.710772000000	-1.355401000000	-1.902793000000
C	-0.493344000000	-3.275747000000	-0.051672000000
C	0.288488000000	-4.425799000000	0.024658000000
C	0.015829000000	-2.069656000000	0.428054000000
C	1.585779000000	-4.376544000000	0.548681000000
H	-0.115934000000	-5.370665000000	-0.316172000000
C	1.309362000000	-1.989362000000	0.948554000000
H	-0.587796000000	-1.172013000000	0.376928000000

C	2.125767000000	-3.144293000000	0.987732000000
N	-5.529066000000	1.066129000000	-0.620847000000
C	-4.700577000000	2.043557000000	-0.069360000000
C	-6.864148000000	1.423574000000	-0.427984000000
C	-5.518867000000	3.013290000000	0.469898000000
C	-6.866411000000	2.631117000000	0.239983000000
H	-5.170945000000	3.906701000000	0.970904000000
H	-7.754876000000	3.178113000000	0.524604000000
C	-8.035500000000	0.596746000000	-0.875878000000
H	-7.782923000000	0.026777000000	-1.778428000000
H	-8.831896000000	1.288232000000	-1.177991000000
C	-8.611340000000	-0.379312000000	0.179128000000
H	-9.372773000000	-0.997725000000	-0.314054000000
H	-7.822277000000	-1.064574000000	0.504932000000
C	-9.229850000000	0.316459000000	1.393606000000
H	-10.059702000000	0.969009000000	1.096859000000
H	-9.622728000000	-0.415033000000	2.107460000000
H	-8.491468000000	0.933464000000	1.914084000000
C	-3.200604000000	2.028522000000	-0.127856000000
H	-2.839580000000	2.638818000000	0.705463000000
H	-2.789680000000	1.025696000000	0.034827000000
C	-2.571767000000	2.579440000000	-1.442011000000
H	-2.824176000000	1.914027000000	-2.274762000000
H	-3.044328000000	3.540381000000	-1.675296000000
C	-1.076756000000	2.694017000000	-1.381636000000
C	-0.122768000000	1.736223000000	-1.654907000000
N	-0.401269000000	3.842052000000	-0.966503000000
C	1.157270000000	2.305299000000	-1.402481000000
H	-0.334521000000	0.732798000000	-1.998187000000
C	0.970314000000	3.604731000000	-0.980355000000
H	2.115618000000	1.823886000000	-1.538022000000
C	1.966236000000	4.669606000000	-0.617083000000
H	1.928215000000	5.490027000000	-1.350807000000
H	1.695438000000	5.122291000000	0.347027000000
C	3.404477000000	4.147593000000	-0.536228000000
H	3.739153000000	3.731864000000	-1.490452000000
H	4.088233000000	4.956594000000	-0.260570000000
H	3.492040000000	3.357523000000	0.216028000000
C	-1.011095000000	5.084994000000	-0.536004000000
H	-1.977542000000	5.206855000000	-1.034710000000
H	-0.390132000000	5.922917000000	-0.874179000000
C	-1.201005000000	5.162026000000	0.952139000000
C	-0.826190000000	4.373014000000	1.998989000000
O	-1.878311000000	6.258523000000	1.409103000000
C	-1.305782000000	5.024292000000	3.186495000000
H	-0.274735000000	3.447340000000	1.927817000000
C	-1.932136000000	6.156772000000	2.772778000000
H	-1.195971000000	4.687044000000	4.206997000000
H	-2.444748000000	6.957100000000	3.282764000000
C	3.551426000000	-3.075573000000	1.539108000000
H	4.033456000000	-4.046780000000	1.392910000000
H	3.481637000000	-2.959089000000	2.624300000000
C	4.493817000000	-2.000210000000	0.995808000000
C	4.724616000000	-1.856349000000	-0.394670000000
C	5.190018000000	-1.165998000000	1.899959000000
C	5.577983000000	-0.846755000000	-0.848048000000

C	6.030409000000	-0.158115000000	1.417143000000
C	6.210317000000	0.012448000000	0.048293000000
H	5.750971000000	-0.717389000000	-1.907232000000
H	6.554144000000	0.485472000000	2.113628000000
N	7.049789000000	1.058603000000	-0.443327000000
C	8.313899000000	1.403155000000	0.077739000000
C	6.723374000000	1.918173000000	-1.511287000000
C	8.804900000000	2.557662000000	-0.745517000000
C	7.883938000000	2.856563000000	-1.664036000000
H	9.766043000000	3.017140000000	-0.557074000000
H	7.891280000000	3.626875000000	-2.423629000000
O	5.704253000000	1.896459000000	-2.168697000000
O	8.879856000000	0.870161000000	1.007633000000
C	1.769001000000	-0.643546000000	1.465110000000
H	2.198303000000	-0.719325000000	2.469469000000
H	2.529748000000	-0.191640000000	0.822956000000
H	0.927652000000	0.051934000000	1.511544000000
C	5.068605000000	-1.314260000000	3.403242000000
H	4.048680000000	-1.134102000000	3.762216000000
H	5.355931000000	-2.316093000000	3.741199000000
H	5.722696000000	-0.600290000000	3.909567000000
C	4.073096000000	-2.793932000000	-1.401884000000
H	4.171375000000	-3.821768000000	-1.031639000000
H	2.994838000000	-2.602036000000	-1.427759000000
C	4.615404000000	-2.740650000000	-2.833622000000
H	4.428326000000	-1.772023000000	-3.307539000000
H	5.692946000000	-2.933300000000	-2.870008000000
H	4.119725000000	-3.501147000000	-3.444364000000
C	2.335754000000	-5.695925000000	0.640154000000
H	1.876752000000	-6.402440000000	-0.059624000000
H	3.374679000000	-5.585023000000	0.309432000000
C	2.311588000000	-6.314584000000	2.049340000000
H	2.795400000000	-5.663625000000	2.784357000000
H	1.281413000000	-6.478722000000	2.380035000000
H	2.830651000000	-7.278799000000	2.060789000000

P1$_{endo-BISM1}$

C	-4.649894000000	-3.753698000000	0.164298000000
C	-4.079035000000	-2.957320000000	1.326566000000
C	-3.803071000000	-1.748887000000	0.828692000000
C	-4.211698000000	-1.799188000000	-0.641065000000
O	-5.310960000000	-2.734610000000	-0.605425000000
H	-5.322546000000	-4.579944000000	0.385926000000
H	-3.863079000000	-3.350366000000	2.311790000000
H	-3.325547000000	-0.908886000000	1.314528000000
C	-4.533449000000	-0.505592000000	-1.382719000000
H	-5.135845000000	-0.762213000000	-2.259346000000
H	-3.588560000000	-0.105092000000	-1.755040000000
C	-2.162845000000	-4.555724000000	-0.181277000000
C	-3.462145000000	-4.098459000000	-0.822167000000
C	-3.159633000000	-2.714349000000	-1.407006000000
C	-1.702205000000	-2.444785000000	-1.098172000000
H	-3.790798000000	-4.835668000000	-1.557893000000
H	-3.330970000000	-2.633017000000	-2.483449000000
N	-1.189126000000	-3.551779000000	-0.397112000000

O	-1.974026000000	-5.592998000000	0.414913000000
O	-1.079771000000	-1.442406000000	-1.387277000000
C	0.154527000000	-3.633473000000	0.093118000000
C	0.860374000000	-4.834178000000	-0.010031000000
C	0.761997000000	-2.513236000000	0.672254000000
C	2.165429000000	-4.908817000000	0.474320000000
H	0.391389000000	-5.704112000000	-0.451537000000
C	2.068741000000	-2.603627000000	1.140697000000
H	0.223875000000	-1.576474000000	0.737241000000
C	2.791171000000	-3.801822000000	1.056768000000
H	2.704208000000	-5.849319000000	0.395165000000
H	2.536723000000	-1.724974000000	1.574961000000
N	-5.195005000000	0.534228000000	-0.614145000000
C	-4.523046000000	1.594016000000	-0.002339000000
C	-6.568453000000	0.676731000000	-0.412535000000
C	-5.476333000000	2.397623000000	0.585201000000
C	-6.750702000000	1.831055000000	0.322871000000
H	-5.267000000000	3.304113000000	1.137307000000
H	-7.710257000000	2.223508000000	0.629873000000
C	-7.604899000000	-0.285320000000	-0.922512000000
H	-7.204675000000	-0.868101000000	-1.758948000000
H	-8.436698000000	0.300740000000	-1.334988000000
C	-8.173477000000	-1.274495000000	0.123726000000
H	-8.807953000000	-1.994738000000	-0.409679000000
H	-7.344953000000	-1.847781000000	0.549048000000
C	-8.987307000000	-0.614729000000	1.238923000000
H	-9.840543000000	-0.056509000000	0.834470000000
H	-9.382308000000	-1.365873000000	1.931255000000
H	-8.375103000000	0.083763000000	1.816780000000
C	-3.041512000000	1.820717000000	-0.084806000000
H	-2.755515000000	2.440193000000	0.770810000000
H	-2.469706000000	0.890010000000	0.010860000000
C	-2.566653000000	2.535841000000	-1.384294000000
H	-2.795947000000	1.903733000000	-2.249687000000
H	-3.168309000000	3.441674000000	-1.516604000000
C	-1.096744000000	2.834603000000	-1.404878000000
C	-0.052119000000	2.002430000000	-1.746645000000
N	-0.541980000000	4.054393000000	-1.018331000000
C	1.161593000000	2.721116000000	-1.564772000000
H	-0.158862000000	0.979305000000	-2.080401000000
C	0.844753000000	3.986099000000	-1.116658000000
H	2.160145000000	2.354753000000	-1.755776000000
C	1.724941000000	5.159280000000	-0.791077000000
H	1.497994000000	6.001676000000	-1.462677000000
H	1.506943000000	5.523001000000	0.223057000000
C	3.218912000000	4.837622000000	-0.896568000000
H	3.486056000000	4.524546000000	-1.910332000000
H	3.819866000000	5.716388000000	-0.644699000000
H	3.496345000000	4.028806000000	-0.213751000000
C	-1.269198000000	5.206688000000	-0.522628000000
H	-2.269712000000	5.222854000000	-0.965946000000
H	-0.771671000000	6.119429000000	-0.870507000000
C	-1.379122000000	5.228448000000	0.975159000000
C	-0.884537000000	4.443161000000	1.973842000000
O	-2.115671000000	6.252443000000	1.502735000000
C	-1.348418000000	5.019058000000	3.205779000000

H	-0.265744000000	3.567879000000	1.843406000000
C	-2.085738000000	6.107626000000	2.863229000000
H	-1.155282000000	4.663198000000	4.207416000000
H	-2.631928000000	6.848391000000	3.425648000000
C	4.202997000000	-3.904850000000	1.609252000000
H	4.164443000000	-3.867446000000	2.705286000000
H	4.605488000000	-4.894123000000	1.357921000000
C	5.162281000000	-2.833213000000	1.120952000000
C	5.376081000000	-2.627138000000	-0.248932000000
C	5.875867000000	-2.039237000000	2.024112000000
C	6.280530000000	-1.673581000000	-0.704471000000
H	4.826541000000	-3.222535000000	-0.972532000000
C	6.773703000000	-1.066307000000	1.586395000000
H	5.728835000000	-2.177618000000	3.092045000000
C	6.981692000000	-0.885535000000	0.216743000000
H	6.431157000000	-1.527908000000	-1.766477000000
H	7.317947000000	-0.464006000000	2.302428000000
N	7.906785000000	0.099690000000	-0.242956000000
C	9.150764000000	0.390165000000	0.357836000000
C	7.717795000000	0.932130000000	-1.367773000000
C	9.769208000000	1.481606000000	-0.463617000000
C	8.939455000000	1.796836000000	-1.459960000000
H	10.739416000000	1.891752000000	-0.217021000000
H	9.049398000000	2.535317000000	-2.242823000000
O	9.617030000000	-0.139878000000	1.343063000000
O	6.757715000000	0.937975000000	-2.107043000000

P1$_{\text{endo-BISM2}}$

C	-4.728235000000	-3.887874000000	0.124685000000
C	-4.218558000000	-3.049444000000	1.285951000000
C	-4.099706000000	-1.809125000000	0.804441000000
C	-4.544199000000	-1.882673000000	-0.653853000000
O	-5.526565000000	-2.940068000000	-0.605251000000
H	-5.293660000000	-4.790217000000	0.349128000000
H	-3.928088000000	-3.430770000000	2.256517000000
H	-3.707876000000	-0.927369000000	1.292968000000
C	-5.036288000000	-0.623339000000	-1.360172000000
H	-5.633526000000	-0.933999000000	-2.222768000000
H	-4.156265000000	-0.110837000000	-1.753411000000
C	-2.176385000000	-4.388662000000	-0.306873000000
C	-3.539396000000	-4.075642000000	-0.901427000000
C	-3.417430000000	-2.656253000000	-1.467263000000
C	-1.991462000000	-2.224613000000	-1.195636000000
H	-3.802735000000	-4.833830000000	-1.642132000000
H	-3.630376000000	-2.578050000000	-2.536519000000
N	-1.333156000000	-3.275975000000	-0.532679000000
O	-1.851406000000	-5.405964000000	0.264937000000
O	-1.497937000000	-1.152389000000	-1.483383000000
C	0.026439000000	-3.208095000000	-0.084986000000
C	0.871036000000	-4.300765000000	-0.254914000000
C	0.507762000000	-2.054023000000	0.528974000000
C	2.200874000000	-4.253101000000	0.177484000000
H	0.491241000000	-5.207201000000	-0.710395000000
C	1.834744000000	-1.973834000000	0.960001000000
H	-0.147430000000	-1.202648000000	0.664779000000
C	2.706266000000	-3.072953000000	0.773359000000

N	-5.787151000000	0.321641000000	-0.551989000000
C	-5.222075000000	1.444211000000	0.055716000000
C	-7.159823000000	0.299724000000	-0.302106000000
C	-6.241167000000	2.122864000000	0.688726000000
C	-7.449325000000	1.414679000000	0.459233000000
H	-6.119481000000	3.040140000000	1.249240000000
H	-8.436660000000	1.687925000000	0.804961000000
C	-8.094123000000	-0.770112000000	-0.794256000000
H	-7.657547000000	-1.290741000000	-1.653133000000
H	-9.002508000000	-0.280257000000	-1.168952000000
C	-8.506166000000	-1.832988000000	0.253024000000
H	-9.069962000000	-2.615953000000	-0.271324000000
H	-7.601671000000	-2.310113000000	0.640560000000
C	-9.352304000000	-1.288431000000	1.405762000000
H	-10.279298000000	-0.830385000000	1.039576000000
H	-9.631077000000	-2.089730000000	2.098431000000
H	-8.806870000000	-0.529488000000	1.974204000000
C	-3.781116000000	1.844413000000	-0.073184000000
H	-3.539448000000	2.483181000000	0.781911000000
H	-3.101175000000	0.986365000000	-0.011987000000
C	-3.440842000000	2.625817000000	-1.376692000000
H	-3.621960000000	1.979911000000	-2.243337000000
H	-4.151567000000	3.453476000000	-1.475786000000
C	-2.019559000000	3.101273000000	-1.441723000000
C	-0.893932000000	2.405538000000	-1.828263000000
N	-1.604619000000	4.377236000000	-1.061155000000
C	0.228494000000	3.266403000000	-1.680353000000
H	-0.887024000000	1.379100000000	-2.168931000000
C	-0.224333000000	4.480097000000	-1.207751000000
H	1.257094000000	3.027127000000	-1.909524000000
C	0.517087000000	5.750075000000	-0.900145000000
H	0.161332000000	6.563654000000	-1.551053000000
H	0.296382000000	6.075237000000	0.126450000000
C	2.033894000000	5.616668000000	-1.067508000000
H	2.296922000000	5.347945000000	-2.094967000000
H	2.531532000000	6.560835000000	-0.827485000000
H	2.436726000000	4.842893000000	-0.406995000000
C	-2.448618000000	5.426694000000	-0.523858000000
H	-3.459405000000	5.323853000000	-0.930646000000
H	-2.079872000000	6.396911000000	-0.876477000000
C	-2.504658000000	5.419983000000	0.977043000000
C	-1.877722000000	4.694176000000	1.945956000000
O	-3.344599000000	6.336999000000	1.545335000000
C	-2.364319000000	5.194548000000	3.201870000000
H	-1.158498000000	3.905858000000	1.780674000000
C	-3.245723000000	6.183980000000	2.901642000000
H	-2.090446000000	4.856402000000	4.190743000000
H	-3.859832000000	6.843917000000	3.493862000000
C	4.150039000000	-3.021368000000	1.277172000000
H	4.110521000000	-3.061242000000	2.369302000000
H	4.656859000000	-3.945132000000	0.983485000000
C	5.052332000000	-1.854419000000	0.870753000000
C	5.254098000000	-1.513401000000	-0.489319000000
C	5.764981000000	-1.151379000000	1.868748000000
C	6.113055000000	-0.460416000000	-0.815723000000
C	6.606549000000	-0.092349000000	1.514363000000

C	6.775404000000	0.258376000000	0.178306000000
H	6.267067000000	-0.187978000000	-1.849796000000
H	7.146542000000	0.444059000000	2.284473000000
N	7.637194000000	1.340877000000	-0.177839000000
C	8.885115000000	1.618547000000	0.418619000000
C	7.367311000000	2.296707000000	-1.180413000000
C	9.420080000000	2.836812000000	-0.273967000000
C	8.541382000000	3.230038000000	-1.197914000000
H	10.377137000000	3.263604000000	-0.005109000000
H	8.587096000000	4.065887000000	-1.883221000000
O	9.412826000000	0.994353000000	1.313893000000
O	6.381811000000	2.346073000000	-1.884280000000
C	2.273898000000	-0.695318000000	1.638586000000
H	2.754394000000	-0.892512000000	2.602379000000
H	2.990672000000	-0.133043000000	1.033680000000
H	1.413194000000	-0.047994000000	1.823909000000
C	5.671557000000	-1.503844000000	3.339918000000
H	4.655353000000	-1.395657000000	3.735930000000
H	5.984155000000	-2.535857000000	3.534923000000
H	6.321074000000	-0.851626000000	3.928554000000
C	4.568346000000	-2.292513000000	-1.603139000000
H	4.700744000000	-3.364146000000	-1.408603000000
H	3.487322000000	-2.124064000000	-1.545539000000
C	5.034645000000	-1.998251000000	-3.032090000000
H	4.824060000000	-0.965605000000	-3.327260000000
H	6.108599000000	-2.173275000000	-3.155980000000
H	4.508011000000	-2.651527000000	-3.734219000000
C	3.017409000000	-5.524765000000	0.010690000000
H	2.559323000000	-6.126418000000	-0.781794000000
H	4.032253000000	-5.302245000000	-0.336528000000
C	3.088988000000	-6.373750000000	1.292764000000
H	3.576356000000	-5.831394000000	2.109056000000
H	2.084651000000	-6.647011000000	1.630454000000
H	3.652806000000	-7.296002000000	1.117794000000

$R2_{endo\text{-}BISM1}$

C	3.678557000000	-2.837198000000	-1.299299000000
C	2.837613000000	-1.572648000000	-1.239254000000
C	3.105444000000	-1.019504000000	-0.053062000000
C	4.120702000000	-1.942352000000	0.615846000000
O	4.832612000000	-2.469082000000	-0.526014000000
H	3.963702000000	-3.224131000000	-2.275707000000
H	2.105417000000	-1.267749000000	-1.975920000000
H	2.652501000000	-0.148298000000	0.400682000000
C	5.052759000000	-1.393123000000	1.696954000000
H	5.972117000000	-1.987002000000	1.676799000000
H	4.580283000000	-1.563260000000	2.665568000000
C	1.521013000000	-4.021632000000	-0.361689000000
C	3.034240000000	-3.899100000000	-0.317613000000
C	3.350149000000	-3.265294000000	1.042771000000
C	2.010222000000	-3.042522000000	1.715505000000
H	3.492070000000	-4.877644000000	-0.476416000000
H	3.994412000000	-3.872688000000	1.683731000000
N	1.004854000000	-3.511803000000	0.852865000000
O	0.852997000000	-4.468228000000	-1.267568000000
O	1.824666000000	-2.528477000000	2.801298000000

C	-0.433629000000	2.653708000000	-0.787238000000
C	0.916036000000	2.666622000000	-0.987118000000
C	1.527405000000	2.461271000000	0.295160000000
C	0.500853000000	2.331324000000	1.179408000000
O	-0.699731000000	2.441096000000	0.538706000000
H	1.418722000000	2.813066000000	-1.932435000000
H	2.583981000000	2.429093000000	0.524835000000
C	-1.607864000000	2.794521000000	-1.705737000000
H	-1.230578000000	2.813982000000	-2.730669000000
H	-2.250373000000	1.912435000000	-1.624436000000
H	0.451021000000	2.176604000000	2.245844000000
N	-2.439760000000	3.974555000000	-1.482064000000
C	-2.248631000000	5.231369000000	-2.055794000000
C	-3.564971000000	4.001416000000	-0.662967000000
C	-3.261607000000	6.043904000000	-1.591973000000
C	-4.080826000000	5.279447000000	-0.718935000000
H	-3.398148000000	7.081206000000	-1.865939000000
H	-4.959883000000	5.627795000000	-0.196389000000
C	-0.396049000000	-3.424860000000	1.138344000000
C	-0.934560000000	-2.248558000000	1.663229000000
C	-1.223359000000	-4.524932000000	0.888152000000
C	-2.298564000000	-2.182656000000	1.946830000000
H	-0.293773000000	-1.397863000000	1.858637000000
C	-2.583634000000	-4.437854000000	1.163524000000
H	-0.805407000000	-5.432619000000	0.470870000000
C	-3.142927000000	-3.270947000000	1.702081000000
C	-1.127658000000	5.573610000000	-2.994724000000
H	-0.868924000000	4.708043000000	-3.618398000000
H	-1.504061000000	6.332773000000	-3.691512000000
C	0.163140000000	6.116135000000	-2.334262000000
H	0.538586000000	5.381535000000	-1.613772000000
H	0.930726000000	6.209365000000	-3.114103000000
C	-0.022971000000	7.467092000000	-1.639855000000
H	0.912387000000	7.806105000000	-1.182756000000
H	-0.343931000000	8.237456000000	-2.351303000000
H	-0.780900000000	7.404270000000	-0.853504000000
C	-4.074777000000	2.783867000000	0.057823000000
H	-4.490915000000	2.057787000000	-0.659142000000
H	-3.257096000000	2.264325000000	0.569937000000
C	-5.157191000000	3.126117000000	1.106007000000
H	-4.707070000000	3.773146000000	1.865792000000
H	-5.946910000000	3.722246000000	0.635840000000
C	-5.756945000000	1.936483000000	1.795406000000
C	-5.395412000000	1.323954000000	2.976721000000
N	-6.858547000000	1.247170000000	1.296570000000
C	-6.303064000000	0.251682000000	3.212638000000
H	-4.573388000000	1.630205000000	3.609989000000
C	-7.206874000000	0.224108000000	2.168666000000
H	-6.309435000000	-0.412949000000	4.064992000000
C	-7.620131000000	1.623279000000	0.115724000000
H	-6.940102000000	2.057683000000	-0.624653000000
H	-8.034759000000	0.725923000000	-0.354120000000
C	-8.713790000000	2.600261000000	0.437965000000
C	-9.125910000000	3.209191000000	1.586096000000
O	-9.488572000000	3.004112000000	-0.613210000000
C	-10.233638000000	4.047716000000	1.221530000000

H	-8.691808000000	3.075727000000	2.565771000000
C	-10.408529000000	3.885760000000	-0.116317000000
H	-10.815290000000	4.683147000000	1.873645000000
H	-11.099322000000	4.297104000000	-0.835394000000
C	-8.394980000000	-0.661147000000	1.920323000000
H	-9.291922000000	-0.041316000000	1.782428000000
H	-8.263938000000	-1.212512000000	0.979016000000
C	-8.650574000000	-1.661616000000	3.051244000000
H	-8.820191000000	-1.148845000000	4.003214000000
H	-9.533293000000	-2.269481000000	2.832387000000
H	-7.801430000000	-2.339706000000	3.179793000000
N	5.373272000000	0.019474000000	1.593221000000
C	4.686144000000	1.033405000000	2.259373000000
C	6.325000000000	0.578837000000	0.744460000000
C	5.212357000000	2.232078000000	1.825536000000
C	6.237782000000	1.948202000000	0.879967000000
H	4.907227000000	3.208506000000	2.177855000000
H	6.851817000000	2.670989000000	0.362461000000
C	3.647757000000	0.777831000000	3.313895000000
H	3.045858000000	1.689663000000	3.408843000000
H	2.949586000000	-0.013549000000	3.010486000000
C	7.243757000000	-0.261572000000	-0.095955000000
H	7.835343000000	-0.930053000000	0.549205000000
H	6.670077000000	-0.925287000000	-0.751755000000
C	8.204796000000	0.582299000000	-0.961618000000
H	8.772432000000	1.267901000000	-0.321717000000
H	7.607339000000	1.215019000000	-1.626388000000
C	9.128485000000	-0.237955000000	-1.812606000000
C	8.968307000000	-0.675848000000	-3.109914000000
N	10.332134000000	-0.760043000000	-1.346342000000
C	10.090693000000	-1.481568000000	-3.442590000000
H	8.120142000000	-0.447860000000	-3.741399000000
C	10.928504000000	-1.522243000000	-2.348017000000
H	10.272385000000	-1.989039000000	-4.380469000000
C	10.905886000000	-0.474077000000	-0.043908000000
H	10.117361000000	-0.510294000000	0.716802000000
H	11.610107000000	-1.271389000000	0.213951000000
C	11.600193000000	0.856316000000	0.015523000000
C	11.802776000000	1.865033000000	-0.878294000000
O	12.162901000000	1.174466000000	1.220811000000
C	12.543427000000	2.878508000000	-0.179721000000
H	11.464007000000	1.881532000000	-1.903379000000
C	12.733041000000	2.410534000000	1.081662000000
H	12.884216000000	3.825720000000	-0.571897000000
H	13.222580000000	2.802342000000	1.959264000000
C	12.246867000000	-2.224280000000	-2.198934000000
H	12.265318000000	-3.029332000000	-2.941630000000
H	12.311528000000	-2.725302000000	-1.223126000000
C	13.488133000000	-1.333622000000	-2.398194000000
H	13.525492000000	-0.521093000000	-1.667197000000
H	14.407162000000	-1.921697000000	-2.301429000000
H	13.476446000000	-0.879925000000	-3.393978000000
C	4.216384000000	0.425288000000	4.706367000000
H	4.858498000000	1.248476000000	5.041792000000
H	4.870590000000	-0.452576000000	4.627065000000
C	3.115515000000	0.150656000000	5.734204000000

H	3.537142000000	-0.089275000000	6.715545000000
H	2.463195000000	1.022907000000	5.858453000000
H	2.488903000000	-0.691748000000	5.421546000000
C	-4.628836000000	-3.193054000000	2.003976000000
H	-4.920691000000	-4.051516000000	2.620107000000
H	-4.826864000000	-2.294539000000	2.599802000000
C	-5.510032000000	-3.154433000000	0.766439000000
C	-6.476392000000	-4.137502000000	0.530465000000
C	-5.391276000000	-2.107066000000	-0.157760000000
C	-7.324462000000	-4.072186000000	-0.575510000000
C	-6.215539000000	-2.036739000000	-1.275199000000
C	-7.196574000000	-3.014735000000	-1.477786000000
H	-8.072520000000	-4.838201000000	-0.739715000000
H	-6.114433000000	-1.220574000000	-1.980359000000
N	-8.070442000000	-2.916932000000	-2.601654000000
C	-8.719355000000	-1.737516000000	-3.013496000000
C	-8.435919000000	-3.985183000000	-3.449180000000
C	-9.532377000000	-2.104331000000	-4.218976000000
C	-9.368031000000	-3.404779000000	-4.471588000000
H	-10.133331000000	-1.370743000000	-4.739547000000
H	-9.796861000000	-4.014293000000	-5.255749000000
O	-8.634401000000	-0.646563000000	-2.488591000000
O	-8.067690000000	-5.135028000000	-3.353370000000
H	-2.712341000000	-1.267623000000	2.362158000000
H	-3.222091000000	-5.292499000000	0.957012000000
H	-6.584411000000	-4.962120000000	1.230018000000
H	-4.643603000000	-1.335957000000	0.001111000000

$R2_{endo\text{-}BISM2}$

C	3.774819000000	-2.464499000000	-1.803979000000
C	2.994100000000	-1.231314000000	-1.379606000000
C	3.332250000000	-1.014556000000	-0.105632000000
C	4.332548000000	-2.110890000000	0.251912000000
O	4.974733000000	-2.345287000000	-1.021467000000
H	4.003042000000	-2.595212000000	-2.860026000000
H	2.248710000000	-0.720212000000	-1.975229000000
H	2.941236000000	-0.272328000000	0.577112000000
C	5.328313000000	-1.884006000000	1.390187000000
H	6.222207000000	-2.478180000000	1.175975000000
H	4.888257000000	-2.281832000000	2.306024000000
C	1.603363000000	-3.764450000000	-1.086692000000
C	3.122008000000	-3.721327000000	-1.096235000000
C	3.528075000000	-3.477647000000	0.361985000000
C	2.232490000000	-3.401576000000	1.144366000000
H	3.520164000000	-4.641901000000	-1.527998000000
H	4.177683000000	-4.248305000000	0.784684000000
N	1.167111000000	-3.579449000000	0.246273000000
O	0.875945000000	-3.919050000000	-2.042536000000
O	2.125579000000	-3.197534000000	2.338476000000
C	0.294100000000	3.003911000000	-0.461293000000
C	1.643848000000	2.844146000000	-0.583735000000
C	2.145973000000	2.537338000000	0.725554000000
C	1.060849000000	2.529589000000	1.546499000000
O	-0.076481000000	2.804927000000	0.841115000000
H	2.217264000000	2.943668000000	-1.494357000000
H	3.173302000000	2.358907000000	1.013335000000

C	-0.793338000000	3.307281000000	-1.445058000000
H	-0.342793000000	3.343475000000	-2.439865000000
H	-1.519912000000	2.488994000000	-1.460383000000
H	0.927299000000	2.367426000000	2.604432000000
N	-1.527899000000	4.546681000000	-1.206083000000
C	-1.165557000000	5.814078000000	-1.661683000000
C	-2.723877000000	4.629129000000	-0.498501000000
C	-2.142176000000	6.689367000000	-1.235641000000
C	-3.112826000000	5.952309000000	-0.505570000000
H	-2.154781000000	7.751091000000	-1.441288000000
H	-4.005270000000	6.349026000000	-0.043598000000
C	-0.215220000000	-3.482507000000	0.613646000000
C	-0.639455000000	-2.462868000000	1.463203000000
C	-1.132393000000	-4.405015000000	0.121772000000
C	-1.985896000000	-2.337887000000	1.813758000000
H	0.091658000000	-1.774616000000	1.861137000000
C	-2.489153000000	-4.296289000000	0.442567000000
H	-0.798178000000	-5.206750000000	-0.525509000000
C	-2.934136000000	-3.240171000000	1.268362000000
C	0.071613000000	6.104639000000	-2.461905000000
H	0.309954000000	5.260955000000	-3.122611000000
H	-0.160030000000	6.942780000000	-3.130780000000
C	1.334504000000	6.468979000000	-1.643897000000
H	1.568598000000	5.651233000000	-0.953878000000
H	2.180758000000	6.540994000000	-2.340073000000
C	1.204738000000	7.779163000000	-0.863925000000
H	2.117082000000	7.990841000000	-0.296828000000
H	1.028066000000	8.625950000000	-1.538027000000
H	0.369724000000	7.736164000000	-0.158470000000
C	-3.408985000000	3.422002000000	0.079687000000
H	-3.786326000000	2.770214000000	-0.723883000000
H	-2.701034000000	2.812046000000	0.652963000000
C	-4.584822000000	3.794649000000	1.009582000000
H	-4.189297000000	4.386222000000	1.841896000000
H	-5.277656000000	4.455490000000	0.477695000000
C	-5.325560000000	2.619321000000	1.577326000000
C	-5.085631000000	1.902217000000	2.730687000000
N	-6.448114000000	2.058327000000	0.974052000000
C	-6.088027000000	0.896176000000	2.842036000000
H	-4.281770000000	2.098255000000	3.427815000000
C	-6.929608000000	1.013880000000	1.754205000000
H	-6.196411000000	0.178915000000	3.643119000000
C	-7.101839000000	2.562392000000	-0.222810000000
H	-6.342001000000	2.946040000000	-0.912512000000
H	-7.591222000000	1.731245000000	-0.741697000000
C	-8.102281000000	3.638548000000	0.085617000000
C	-8.517293000000	4.240396000000	1.236020000000
O	-8.766811000000	4.163674000000	-0.987430000000
C	-9.508819000000	5.205251000000	0.849704000000
H	-8.158836000000	4.022324000000	2.230964000000
C	-9.619321000000	5.116847000000	-0.501767000000
H	-10.059710000000	5.871835000000	1.497377000000
H	-10.219582000000	5.627654000000	-1.238101000000
C	-8.177046000000	0.259082000000	1.391597000000
H	-9.011501000000	0.965386000000	1.276588000000
H	-8.061229000000	-0.224896000000	0.412635000000

C	-8.561219000000	-0.799928000000	2.428891000000
H	-8.732059000000	-0.349410000000	3.411788000000
H	-9.478134000000	-1.314938000000	2.128252000000
H	-7.774030000000	-1.552670000000	2.532517000000
N	5.699815000000	-0.500513000000	1.630167000000
C	5.068050000000	0.335865000000	2.549238000000
C	6.645770000000	0.224590000000	0.910453000000
C	5.624813000000	1.589420000000	2.405548000000
C	6.612328000000	1.519800000000	1.382371000000
H	5.365799000000	2.456398000000	2.998818000000
H	7.237549000000	2.330553000000	1.037248000000
C	4.047739000000	-0.143411000000	3.541478000000
H	3.472716000000	0.730279000000	3.870733000000
H	3.320690000000	-0.822441000000	3.076958000000
C	7.505953000000	-0.406846000000	-0.146943000000
H	8.095460000000	-1.228451000000	0.289539000000
H	6.886901000000	-0.873312000000	-0.920949000000
C	8.466100000000	0.599042000000	-0.818739000000
H	9.079277000000	1.090822000000	-0.054643000000
H	7.869061000000	1.391365000000	-1.282392000000
C	9.330769000000	-0.010088000000	-1.882419000000
C	9.109961000000	-0.113703000000	-3.239225000000
N	10.531874000000	-0.659150000000	-1.608911000000
C	10.191972000000	-0.840516000000	-3.805425000000
H	8.248104000000	0.282038000000	-3.759540000000
C	11.066243000000	-1.167628000000	-2.790367000000
H	10.323099000000	-1.107541000000	-4.845452000000
C	11.160281000000	-0.714926000000	-0.301502000000
H	10.398211000000	-0.917174000000	0.460174000000
H	11.845659000000	-1.568010000000	-0.275898000000
C	11.900900000000	0.543227000000	0.050745000000
C	12.108326000000	1.733579000000	-0.579702000000
O	12.513612000000	0.544380000000	1.273509000000
C	12.905960000000	2.527692000000	0.312965000000
H	11.736782000000	2.007252000000	-1.555875000000
C	13.120927000000	1.762360000000	1.414834000000
H	13.265874000000	3.532939000000	0.148372000000
H	13.652225000000	1.916528000000	2.340740000000
C	12.364584000000	-1.916426000000	-2.872943000000
H	12.329654000000	-2.515159000000	-3.789644000000
H	12.446501000000	-2.642698000000	-2.052431000000
C	13.627572000000	-1.034485000000	-2.901388000000
H	13.718599000000	-0.427502000000	-1.996274000000
H	14.528898000000	-1.650477000000	-2.990719000000
H	13.595951000000	-0.350009000000	-3.754655000000
C	4.638796000000	-0.836662000000	4.788996000000
H	5.311006000000	-0.134015000000	5.295932000000
H	5.266510000000	-1.682122000000	4.478928000000
C	3.554925000000	-1.330887000000	5.750643000000
H	3.992336000000	-1.811668000000	6.631536000000
H	2.929347000000	-0.502000000000	6.101904000000
H	2.899138000000	-2.058604000000	5.260279000000
C	-4.412813000000	-3.104534000000	1.631349000000
H	-4.657733000000	-3.929977000000	2.305937000000
H	-4.549629000000	-2.200005000000	2.230080000000
C	-5.468167000000	-3.055561000000	0.523185000000

C	-6.614850000000	-3.877537000000	0.628622000000
C	-5.377184000000	-2.131202000000	-0.547999000000
C	-7.641555000000	-3.774212000000	-0.316219000000
C	-6.406250000000	-2.071478000000	-1.491077000000
C	-7.538208000000	-2.873930000000	-1.370522000000
H	-8.521373000000	-4.400477000000	-0.226579000000
H	-6.348350000000	-1.373007000000	-2.313962000000
N	-8.599409000000	-2.740913000000	-2.317035000000
C	-9.149742000000	-1.515038000000	-2.735216000000
C	-9.270290000000	-3.808442000000	-2.949070000000
C	-10.232373000000	-1.854406000000	-3.716681000000
C	-10.302686000000	-3.181741000000	-3.840215000000
H	-10.819900000000	-1.084496000000	-4.198795000000
H	-10.960855000000	-3.783051000000	-4.453099000000
O	-8.813073000000	-0.408365000000	-2.366177000000
O	-9.050217000000	-4.989123000000	-2.790433000000
C	-6.811706000000	-4.862015000000	1.764165000000
H	-6.021550000000	-5.619907000000	1.801825000000
H	-6.829483000000	-4.366496000000	2.741419000000
H	-7.761359000000	-5.389944000000	1.648879000000
C	-3.430758000000	-5.344001000000	-0.108080000000
H	-4.039997000000	-5.794824000000	0.682467000000
H	-4.123251000000	-4.930670000000	-0.846846000000
H	-2.868319000000	-6.148251000000	-0.589000000000
C	-4.197048000000	-1.178700000000	-0.673285000000
H	-4.140098000000	-0.583200000000	0.246975000000
H	-3.269622000000	-1.757106000000	-0.707043000000
C	-4.214141000000	-0.222958000000	-1.869249000000
H	-4.211743000000	-0.761002000000	-2.823117000000
H	-5.084922000000	0.440269000000	-1.857428000000
H	-3.318740000000	0.405808000000	-1.848759000000
C	-2.406674000000	-1.241178000000	2.786839000000
H	-3.171575000000	-0.605275000000	2.324255000000
H	-2.904329000000	-1.710529000000	3.646194000000
C	-1.293067000000	-0.332546000000	3.315554000000
H	-0.519798000000	-0.896694000000	3.846826000000
H	-0.812044000000	0.229902000000	2.508662000000
H	-1.715280000000	0.394533000000	4.015802000000

TS2$_{\text{endo-BISM1}}$

C	2.195718000000	-0.974043000000	-1.969767000000
C	1.035206000000	-0.518345000000	-1.096553000000
C	1.406276000000	-0.923558000000	0.209344000000
C	2.733421000000	-1.662750000000	0.012064000000
O	3.317812000000	-0.913617000000	-1.071427000000
H	2.409257000000	-0.412889000000	-2.879379000000
H	0.021116000000	-0.632113000000	-1.465949000000
H	0.680001000000	-1.298344000000	0.921623000000
C	3.705926000000	-1.957101000000	1.158258000000
H	4.590195000000	-2.427306000000	0.717458000000
H	3.225952000000	-2.717427000000	1.779589000000
C	0.709187000000	-3.082334000000	-2.471241000000
C	2.091011000000	-2.532828000000	-2.170047000000
C	2.426256000000	-3.025949000000	-0.758587000000
C	1.206705000000	-3.792309000000	-0.289058000000
H	2.793350000000	-2.859896000000	-2.940383000000

H	3.301241000000	-3.680844000000	-0.713797000000
N	0.266650000000	-3.805965000000	-1.337858000000
O	0.073550000000	-2.933072000000	-3.492090000000
O	1.049723000000	-4.299408000000	0.803619000000
C	0.807371000000	1.578499000000	-0.886672000000
C	2.163969000000	1.986999000000	-0.756653000000
C	2.552615000000	1.663360000000	0.522354000000
C	1.423190000000	1.070875000000	1.133830000000
O	0.314471000000	1.375037000000	0.388399000000
H	2.789942000000	2.338230000000	-1.563600000000
H	3.540198000000	1.711053000000	0.957492000000
C	-0.213707000000	1.985335000000	-1.923775000000
H	0.143889000000	1.656561000000	-2.901256000000
H	-1.135811000000	1.431803000000	-1.725590000000
H	1.243694000000	0.901873000000	2.184985000000
N	-0.536895000000	3.408103000000	-2.008645000000
C	-0.047641000000	4.310361000000	-2.958037000000
C	-1.499630000000	4.041074000000	-1.223583000000
C	-0.704076000000	5.506079000000	-2.754120000000
C	-1.604666000000	5.341044000000	-1.669045000000
H	-0.555650000000	6.402680000000	-3.339927000000
H	-2.266471000000	6.092580000000	-1.263425000000
C	-1.029052000000	-4.408279000000	-1.247906000000
C	-1.791597000000	-4.268134000000	-0.082029000000
C	-1.542694000000	-5.118424000000	-2.335042000000
C	-3.066123000000	-4.820727000000	-0.023100000000
H	-1.387566000000	-3.735336000000	0.768912000000
C	-2.826415000000	-5.658919000000	-2.260950000000
H	-0.956899000000	-5.225512000000	-3.238896000000
C	-3.611822000000	-5.512810000000	-1.113468000000
C	1.001947000000	3.990648000000	-3.986063000000
H	1.052857000000	2.910628000000	-4.166308000000
H	0.685676000000	4.427632000000	-4.942013000000
C	2.429085000000	4.504545000000	-3.672716000000
H	2.737482000000	4.141900000000	-2.685825000000
H	3.119223000000	4.059940000000	-4.401875000000
C	2.568408000000	6.028172000000	-3.709297000000
H	3.599120000000	6.332123000000	-3.499638000000
H	2.297801000000	6.426344000000	-4.694448000000
H	1.920326000000	6.500620000000	-2.965934000000
C	-2.265581000000	3.329659000000	-0.144338000000
H	-2.900903000000	2.541554000000	-0.579140000000
H	-1.585473000000	2.810935000000	0.538303000000
C	-3.152809000000	4.279565000000	0.691844000000
H	-2.514768000000	5.056359000000	1.125770000000
H	-3.859248000000	4.802183000000	0.035486000000
C	-3.868708000000	3.589513000000	1.814682000000
C	-3.478656000000	3.426454000000	3.126856000000
N	-5.064888000000	2.896688000000	1.652712000000
C	-4.456560000000	2.626911000000	3.781796000000
H	-2.573177000000	3.829713000000	3.560228000000
C	-5.432791000000	2.313358000000	2.859662000000
H	-4.444112000000	2.310632000000	4.815050000000
C	-5.900313000000	2.917893000000	0.463194000000
H	-5.259628000000	3.082498000000	-0.409523000000
H	-6.365766000000	1.940249000000	0.311275000000

C	-6.962929000000	3.977442000000	0.533547000000
C	-7.128996000000	5.097496000000	1.291660000000
O	-8.001766000000	3.854196000000	-0.353288000000
C	-8.346665000000	5.714206000000	0.845406000000
H	-6.464321000000	5.435726000000	2.072693000000
C	-8.830995000000	4.926077000000	-0.149360000000
H	-8.795028000000	6.622932000000	1.220426000000
H	-9.700543000000	4.974669000000	-0.785921000000
C	-6.694119000000	1.511059000000	3.001747000000
H	-7.565274000000	2.138396000000	2.762530000000
H	-6.703322000000	0.697340000000	2.262534000000
C	-6.875917000000	0.917766000000	4.401997000000
H	-6.920870000000	1.704474000000	5.161506000000
H	-7.803759000000	0.340830000000	4.459538000000
H	-6.046893000000	0.251146000000	4.658948000000
N	4.146731000000	-0.866977000000	2.008518000000
C	3.555084000000	-0.496365000000	3.213394000000
C	5.246320000000	-0.051700000000	1.757905000000
C	4.272949000000	0.571305000000	3.708702000000
C	5.333829000000	0.848298000000	2.801585000000
H	4.064258000000	1.084395000000	4.637828000000
H	6.084531000000	1.618326000000	2.906698000000
C	2.422965000000	-1.251852000000	3.847445000000
H	1.683930000000	-1.563303000000	3.098596000000
H	1.895419000000	-0.564298000000	4.519621000000
C	6.122630000000	-0.233217000000	0.550727000000
H	6.583968000000	-1.233398000000	0.568318000000
H	5.525510000000	-0.202513000000	-0.367012000000
C	7.240283000000	0.828292000000	0.451721000000
H	7.834230000000	0.826152000000	1.372954000000
H	6.776419000000	1.818740000000	0.394716000000
C	8.123419000000	0.661917000000	-0.749416000000
C	8.009235000000	1.218764000000	-2.005335000000
N	9.220717000000	-0.194878000000	-0.775732000000
C	9.052054000000	0.692574000000	-2.814721000000
H	7.242596000000	1.920232000000	-2.305912000000
C	9.796756000000	-0.174989000000	-2.043672000000
H	9.242562000000	0.914685000000	-3.856104000000
C	9.726558000000	-0.923373000000	0.373668000000
H	8.885737000000	-1.349404000000	0.933551000000
H	10.315123000000	-1.775606000000	0.019574000000
C	10.560941000000	-0.074425000000	1.289402000000
C	10.943113000000	1.233784000000	1.289508000000
O	11.064560000000	-0.712818000000	2.389073000000
C	11.740126000000	1.419609000000	2.470159000000
H	10.686122000000	1.969744000000	0.542400000000
C	11.780550000000	0.214399000000	3.096146000000
H	12.214843000000	2.331974000000	2.800854000000
H	12.244649000000	-0.143014000000	4.001798000000
C	11.010832000000	-0.969222000000	-2.428961000000
H	10.991555000000	-1.072341000000	-3.519375000000
H	10.944760000000	-1.993710000000	-2.037209000000
C	12.357574000000	-0.348897000000	-2.010431000000
H	12.433790000000	-0.240189000000	-0.924934000000
H	13.193069000000	-0.970359000000	-2.350224000000
H	12.472060000000	0.646857000000	-2.449611000000

C	2.861312000000	-2.489440000000	4.661516000000
H	3.548016000000	-2.161191000000	5.450754000000
H	3.440416000000	-3.166535000000	4.020748000000
C	1.676414000000	-3.246514000000	5.266536000000
H	1.086301000000	-2.599301000000	5.925599000000
H	1.008396000000	-3.623584000000	4.484085000000
H	2.012711000000	-4.104193000000	5.857611000000
C	-5.035317000000	-6.037946000000	-1.058588000000
H	-5.231841000000	-6.632876000000	-1.959418000000
H	-5.149890000000	-6.722374000000	-0.209746000000
C	-6.073415000000	-4.930503000000	-0.940865000000
C	-7.108613000000	-5.001901000000	-0.002538000000
C	-6.007706000000	-3.802267000000	-1.769712000000
C	-8.035793000000	-3.968404000000	0.134815000000
C	-6.931466000000	-2.770254000000	-1.654978000000
C	-7.938018000000	-2.844183000000	-0.686898000000
H	-8.823165000000	-4.031511000000	0.876473000000
H	-6.859085000000	-1.898321000000	-2.293682000000
N	-8.835655000000	-1.746247000000	-0.526688000000
C	-8.432282000000	-0.402249000000	-0.450120000000
C	-10.236456000000	-1.833064000000	-0.376537000000
C	-9.678883000000	0.408563000000	-0.268276000000
C	-10.724770000000	-0.421012000000	-0.225559000000
H	-9.637901000000	1.487630000000	-0.193841000000
H	-11.776363000000	-0.197589000000	-0.104594000000
O	-7.287765000000	0.000709000000	-0.512786000000
O	-10.893399000000	-2.850397000000	-0.366978000000
H	-3.222730000000	-6.197338000000	-3.117770000000
H	-3.657616000000	-4.690471000000	0.878856000000
H	-7.185022000000	-5.869614000000	0.647418000000
H	-5.211637000000	-3.721375000000	-2.503597000000

TS2$_{endo\text{-}BISM2}$

C	2.311272000000	-0.922249000000	-1.535436000000
C	1.523589000000	0.093395000000	-0.719819000000
C	2.031215000000	-0.049151000000	0.595951000000
C	3.029227000000	-1.206689000000	0.488518000000
O	3.565373000000	-0.996025000000	-0.832622000000
H	2.482003000000	-0.715504000000	-2.591688000000
H	0.463595000000	0.217931000000	-0.914554000000
H	1.388799000000	0.027459000000	1.465975000000
C	4.093594000000	-1.495173000000	1.551084000000
H	4.703295000000	-2.324253000000	1.179226000000
H	3.557929000000	-1.870280000000	2.426734000000
C	0.231314000000	-2.496583000000	-1.199773000000
C	1.743102000000	-2.357675000000	-1.230686000000
C	2.198471000000	-2.540464000000	0.221844000000
C	0.927576000000	-2.705032000000	1.033163000000
H	2.165462000000	-3.083178000000	-1.929893000000
H	2.838331000000	-3.413996000000	0.377835000000
N	-0.160895000000	-2.702507000000	0.142316000000
O	-0.526751000000	-2.436603000000	-2.143079000000
O	0.850143000000	-2.809469000000	2.241088000000
C	1.913862000000	2.151458000000	-1.125266000000
C	3.325220000000	2.113884000000	-1.304798000000
C	3.878938000000	2.003547000000	-0.050556000000

C	2.789659000000	1.974286000000	0.853093000000
O	1.673227000000	2.436545000000	0.202538000000
H	3.830478000000	2.032888000000	-2.256708000000
H	4.910848000000	1.829954000000	0.217455000000
C	0.852380000000	2.605627000000	-2.098498000000
H	1.093424000000	2.186463000000	-3.078126000000
H	-0.108888000000	2.178171000000	-1.804163000000
H	2.806696000000	2.143026000000	1.919434000000
N	0.673182000000	4.049336000000	-2.228377000000
C	1.261868000000	4.877993000000	-3.185710000000
C	-0.234640000000	4.787765000000	-1.471447000000
C	0.718716000000	6.134792000000	-3.020115000000
C	-0.210548000000	6.081087000000	-1.947137000000
H	0.964361000000	7.000240000000	-3.620039000000
H	-0.802962000000	6.901662000000	-1.568848000000
C	-1.527681000000	-2.848759000000	0.546467000000
C	-2.031394000000	-2.098276000000	1.605522000000
C	-2.347625000000	-3.757321000000	-0.112897000000
C	-3.363177000000	-2.235374000000	2.006408000000
H	-1.371907000000	-1.419775000000	2.127642000000
C	-3.686621000000	-3.912204000000	0.259135000000
H	-1.947075000000	-4.351317000000	-0.926041000000
C	-4.216601000000	-3.129795000000	1.309953000000
C	2.306143000000	4.432449000000	-4.169396000000
H	2.223485000000	3.355267000000	-4.358234000000
H	2.093046000000	4.913358000000	-5.132575000000
C	3.769504000000	4.756597000000	-3.778238000000
H	3.977670000000	4.342970000000	-2.785424000000
H	4.433575000000	4.237296000000	-4.482314000000
C	4.098222000000	6.251179000000	-3.783043000000
H	5.144507000000	6.422715000000	-3.509888000000
H	3.937154000000	6.689555000000	-4.775228000000
H	3.471010000000	6.794271000000	-3.070427000000
C	-1.064886000000	4.175962000000	-0.378139000000
H	-1.777929000000	3.446737000000	-0.795844000000
H	-0.433265000000	3.608804000000	0.313117000000
C	-1.847239000000	5.228421000000	0.438400000000
H	-1.131411000000	5.951560000000	0.842037000000
H	-2.508140000000	5.797788000000	-0.226139000000
C	-2.617723000000	4.654196000000	1.589895000000
C	-2.255368000000	4.535413000000	2.914045000000
N	-3.883051000000	4.089311000000	1.459265000000
C	-3.322030000000	3.898747000000	3.608895000000
H	-1.316504000000	4.871076000000	3.333453000000
C	-4.324720000000	3.637876000000	2.698473000000
H	-3.353307000000	3.660464000000	4.662400000000
C	-4.692731000000	4.120811000000	0.255013000000
H	-4.038447000000	4.040046000000	-0.619489000000
H	-5.337638000000	3.235336000000	0.232868000000
C	-5.528711000000	5.363821000000	0.157318000000
C	-5.658649000000	6.477388000000	0.932019000000
O	-6.351324000000	5.447007000000	-0.931698000000
C	-6.629559000000	7.309864000000	0.278061000000
H	-5.127340000000	6.676485000000	1.850776000000
C	-7.013771000000	6.640617000000	-0.840417000000
H	-6.987644000000	8.275760000000	0.603656000000

H	-7.708558000000	6.853013000000	-1.637624000000
C	-5.670801000000	2.996411000000	2.881160000000
H	-6.450035000000	3.625505000000	2.429966000000
H	-5.710358000000	2.036466000000	2.342809000000
C	-6.023987000000	2.746104000000	4.350893000000
H	-6.032467000000	3.681903000000	4.917754000000
H	-7.013330000000	2.287426000000	4.435955000000
H	-5.300330000000	2.074843000000	4.824015000000
N	4.983155000000	-0.427081000000	1.968413000000
C	4.776107000000	0.411063000000	3.060951000000
C	6.189104000000	-0.103957000000	1.353805000000
C	5.845483000000	1.279378000000	3.117261000000
C	6.732416000000	0.956135000000	2.052449000000
H	5.980895000000	2.052914000000	3.861176000000
H	7.671088000000	1.442114000000	1.828247000000
C	3.634070000000	0.251185000000	4.022693000000
H	2.701021000000	0.012989000000	3.496675000000
H	3.464960000000	1.220500000000	4.507233000000
C	6.715961000000	-0.863410000000	0.168901000000
H	6.880492000000	-1.917970000000	0.441561000000
H	5.974972000000	-0.881933000000	-0.637507000000
C	8.035733000000	-0.280425000000	-0.382588000000
H	8.782679000000	-0.235042000000	0.418326000000
H	7.858302000000	0.756280000000	-0.687919000000
C	8.573744000000	-1.025610000000	-1.568152000000
C	8.359465000000	-0.806713000000	-2.912267000000
N	9.367105000000	-2.164434000000	-1.456617000000
C	9.030389000000	-1.829519000000	-3.635577000000
H	7.767382000000	0.000019000000	-3.323080000000
C	9.653330000000	-2.658889000000	-2.726816000000
H	9.055502000000	-1.957744000000	-4.709367000000
C	9.881702000000	-2.692426000000	-0.205977000000
H	9.090908000000	-2.666422000000	0.552911000000
H	10.130518000000	-3.749153000000	-0.345471000000
C	11.085571000000	-1.948673000000	0.297114000000
C	11.798587000000	-0.877362000000	-0.151609000000
O	11.614227000000	-2.408322000000	1.471709000000
C	12.842725000000	-0.658010000000	0.810345000000
H	11.601167000000	-0.316725000000	-1.053030000000
C	12.684534000000	-1.608920000000	1.767840000000
H	13.604281000000	0.107822000000	0.784579000000
H	13.211874000000	-1.849722000000	2.677382000000
C	10.499698000000	-3.871065000000	-2.986731000000
H	10.229454000000	-4.241315000000	-3.981656000000
H	10.239580000000	-4.682023000000	-2.292401000000
C	12.020459000000	-3.628416000000	-2.940992000000
H	12.344788000000	-3.272600000000	-1.959081000000
H	12.567183000000	-4.550190000000	-3.167136000000
H	12.308494000000	-2.871385000000	-3.676833000000
C	3.871628000000	-0.808471000000	5.121435000000
H	4.767951000000	-0.528083000000	5.687295000000
H	4.098906000000	-1.776525000000	4.657159000000
C	2.673234000000	-0.961031000000	6.061525000000
H	2.431380000000	-0.012067000000	6.553979000000
H	1.782508000000	-1.289433000000	5.514373000000
H	2.873503000000	-1.699618000000	6.844130000000

C	-5.670142000000	-3.293385000000	1.763925000000
H	-5.725016000000	-4.217045000000	2.347912000000
H	-5.903883000000	-2.499078000000	2.477820000000
C	-6.791349000000	-3.305339000000	0.725234000000
C	-7.728749000000	-4.362915000000	0.723565000000
C	-6.960085000000	-2.232810000000	-0.184147000000
C	-8.781840000000	-4.362656000000	-0.196094000000
C	-8.007344000000	-2.270377000000	-1.108881000000
C	-8.913398000000	-3.329867000000	-1.119731000000
H	-9.492711000000	-5.179607000000	-0.197559000000
H	-8.141040000000	-1.459881000000	-1.810704000000
N	-9.982155000000	-3.347441000000	-2.067409000000
C	-10.750832000000	-2.227526000000	-2.448614000000
C	-10.428607000000	-4.489677000000	-2.765336000000
C	-11.743388000000	-2.723581000000	-3.458099000000
C	-11.556027000000	-4.031775000000	-3.642450000000
H	-12.463462000000	-2.057589000000	-3.914547000000
H	-12.079812000000	-4.719988000000	-4.292378000000
O	-10.627292000000	-1.093276000000	-2.039154000000
O	-9.985772000000	-5.613641000000	-2.667454000000
C	-7.663209000000	-5.511921000000	1.709614000000
H	-6.733348000000	-6.085679000000	1.624623000000
H	-7.733915000000	-5.167905000000	2.747734000000
H	-8.488649000000	-6.207062000000	1.538860000000
C	-4.501482000000	-4.947210000000	-0.485635000000
H	-5.039896000000	-5.611208000000	0.196950000000
H	-5.248839000000	-4.492224000000	-1.141871000000
H	-3.848551000000	-5.568824000000	-1.103750000000
C	-6.023793000000	-1.032866000000	-0.157884000000
H	-5.948963000000	-0.679680000000	0.879216000000
H	-5.011274000000	-1.358801000000	-0.419927000000
C	-6.406794000000	0.152080000000	-1.048953000000
H	-6.387661000000	-0.111791000000	-2.111173000000
H	-7.405007000000	0.536232000000	-0.816049000000
H	-5.689949000000	0.967109000000	-0.908244000000
C	-3.868033000000	-1.431957000000	3.201272000000
H	-4.709289000000	-0.800537000000	2.884197000000
H	-4.290449000000	-2.127424000000	3.938335000000
C	-2.845493000000	-0.541324000000	3.913698000000
H	-1.993246000000	-1.118544000000	4.286018000000
H	-2.465084000000	0.251360000000	3.262088000000
H	-3.316600000000	-0.054883000000	4.773068000000

P2$_{endo\text{-}BISM1}$

C	1.775232000000	-0.556108000000	-1.794279000000
C	1.102886000000	0.560377000000	-0.968910000000
C	1.337752000000	0.053018000000	0.492265000000
C	2.006798000000	-1.322955000000	0.234787000000
O	2.812995000000	-1.064762000000	-0.933933000000
H	2.204144000000	-0.268932000000	-2.754129000000
H	0.043310000000	0.661924000000	-1.214865000000
H	0.409713000000	-0.050703000000	1.057698000000
C	2.771218000000	-2.062963000000	1.340010000000
H	3.252054000000	-2.927949000000	0.872143000000
H	2.017240000000	-2.453830000000	2.028436000000
C	-0.650481000000	-1.547979000000	-2.071557000000

C	0.830948000000	-1.799851000000	-1.846583000000
C	0.941662000000	-2.305818000000	-0.400089000000
C	-0.468187000000	-2.268068000000	0.157781000000
H	1.192993000000	-2.508277000000	-2.595115000000
H	1.314695000000	-3.331352000000	-0.324328000000
N	-1.333925000000	-1.842440000000	-0.868642000000
O	-1.175070000000	-1.158798000000	-3.091672000000
O	-0.802083000000	-2.548036000000	1.290807000000
C	1.713695000000	2.019167000000	-0.906465000000
C	3.227928000000	1.913209000000	-0.792772000000
C	3.470344000000	1.469092000000	0.441959000000
C	2.109527000000	1.300355000000	1.090504000000
O	1.347772000000	2.343804000000	0.451095000000
H	3.930120000000	2.055630000000	-1.602277000000
H	4.409909000000	1.178229000000	0.891839000000
C	1.086101000000	2.975494000000	-1.919573000000
H	1.348082000000	2.626328000000	-2.922434000000
H	-0.000983000000	2.886147000000	-1.839066000000
H	2.056020000000	1.398217000000	2.172356000000
N	1.433696000000	4.383872000000	-1.800110000000
C	2.410433000000	5.070918000000	-2.528062000000
C	0.698664000000	5.294254000000	-1.041806000000
C	2.284531000000	6.407217000000	-2.210378000000
C	1.222078000000	6.546380000000	-1.278895000000
H	2.886908000000	7.204275000000	-2.622767000000
H	0.873630000000	7.468203000000	-0.836086000000
C	-2.750132000000	-1.694515000000	-0.707532000000
C	-3.266686000000	-1.086090000000	0.437661000000
C	-3.615670000000	-2.166186000000	-1.701157000000
C	-4.647523000000	-0.955891000000	0.583390000000
H	-2.601248000000	-0.731481000000	1.214452000000
C	-4.989576000000	-2.021335000000	-1.542673000000
H	-3.214319000000	-2.630029000000	-2.593423000000
C	-5.529659000000	-1.414689000000	-0.399822000000
C	3.379888000000	4.422733000000	-3.478550000000
H	3.065308000000	3.397350000000	-3.702451000000
H	3.337908000000	4.953510000000	-4.439307000000
C	4.857795000000	4.387041000000	-3.015529000000
H	4.914719000000	3.931361000000	-2.020838000000
H	5.409527000000	3.727139000000	-3.698570000000
C	5.546508000000	5.753459000000	-2.978164000000
H	6.599188000000	5.650695000000	-2.694946000000
H	5.511943000000	6.242792000000	-3.958846000000
H	5.069205000000	6.416952000000	-2.252221000000
C	-0.476266000000	4.886349000000	-0.198462000000
H	-1.277687000000	4.476277000000	-0.834534000000
H	-0.197153000000	4.077601000000	0.483547000000
C	-1.052568000000	6.054074000000	0.631797000000
H	-0.265250000000	6.432352000000	1.291649000000
H	-1.315531000000	6.886834000000	-0.031789000000
C	-2.224456000000	5.668515000000	1.484672000000
C	-2.262982000000	5.291105000000	2.809196000000
N	-3.527162000000	5.592200000000	0.999399000000
C	-3.613331000000	4.989701000000	3.144351000000
H	-1.403141000000	5.231313000000	3.462713000000
C	-4.383866000000	5.188872000000	2.018487000000

H	-3.980525000000	4.660199000000	4.105805000000
C	-3.958094000000	6.034045000000	-0.314031000000
H	-3.161050000000	5.836091000000	-1.038369000000
H	-4.814111000000	5.429753000000	-0.633811000000
C	-4.324568000000	7.489731000000	-0.338431000000
C	-4.277487000000	8.490250000000	0.585488000000
O	-4.806854000000	7.965804000000	-1.526342000000
C	-4.763897000000	9.669567000000	-0.074831000000
H	-3.937174000000	8.395173000000	1.605864000000
C	-5.068121000000	9.296737000000	-1.345616000000
H	-4.869421000000	10.658490000000	0.347238000000
H	-5.458610000000	9.818602000000	-2.205037000000
C	-5.862590000000	5.039171000000	1.802410000000
H	-6.285481000000	5.985439000000	1.436085000000
H	-6.056310000000	4.301487000000	1.008645000000
C	-6.616023000000	4.612225000000	3.065765000000
H	-6.487852000000	5.346378000000	3.866992000000
H	-7.686542000000	4.516980000000	2.862266000000
H	-6.254586000000	3.647417000000	3.434949000000
N	3.764926000000	-1.337323000000	2.107738000000
C	3.538413000000	-0.749554000000	3.352635000000
C	5.104050000000	-1.182552000000	1.753898000000
C	4.730456000000	-0.193304000000	3.761731000000
C	5.709924000000	-0.466023000000	2.764285000000
H	4.890833000000	0.336951000000	4.689216000000
H	6.750720000000	-0.177492000000	2.793979000000
C	2.217938000000	-0.851449000000	4.064921000000
H	1.984970000000	-1.907886000000	4.271492000000
H	1.395830000000	-0.496982000000	3.427086000000
C	5.676551000000	-1.768598000000	0.494538000000
H	5.566990000000	-2.864682000000	0.507322000000
H	5.112922000000	-1.428955000000	-0.380640000000
C	7.167463000000	-1.419577000000	0.291530000000
H	7.743023000000	-1.731285000000	1.170689000000
H	7.265942000000	-0.330186000000	0.237501000000
C	7.755707000000	-1.999706000000	-0.960527000000
C	7.847214000000	-1.451643000000	-2.222154000000
N	8.265770000000	-3.293255000000	-1.034206000000
C	8.420405000000	-2.426230000000	-3.083056000000
H	7.519984000000	-0.456451000000	-2.491705000000
C	8.679571000000	-3.557154000000	-2.337656000000
H	8.621235000000	-2.322480000000	-4.140888000000
C	8.416514000000	-4.186796000000	0.100089000000
H	7.518088000000	-4.135046000000	0.726330000000
H	8.468584000000	-5.216563000000	-0.267262000000
C	9.627908000000	-3.882520000000	0.933982000000
C	10.616124000000	-2.946038000000	0.871434000000
O	9.818140000000	-4.697137000000	2.015980000000
C	11.480026000000	-3.197879000000	1.991232000000
H	10.712628000000	-2.174596000000	0.122153000000
C	10.952166000000	-4.264033000000	2.647494000000
H	12.372008000000	-2.652628000000	2.263607000000
H	11.236183000000	-4.814513000000	3.530468000000
C	9.296708000000	-4.852650000000	-2.778276000000
H	9.150889000000	-4.923185000000	-3.861678000000
H	8.751045000000	-5.706679000000	-2.353593000000

C	10.798201000000	-5.000540000000	-2.466351000000
H	10.995395000000	-4.953217000000	-1.391730000000
H	11.179864000000	-5.956337000000	-2.841176000000
H	11.367759000000	-4.195717000000	-2.941071000000
C	2.175480000000	-0.078453000000	5.390525000000
H	2.968464000000	-0.451525000000	6.049541000000
H	2.404808000000	0.977405000000	5.200810000000
C	0.821805000000	-0.195676000000	6.095699000000
H	0.012163000000	0.201755000000	5.473460000000
H	0.580767000000	-1.240075000000	6.323416000000
H	0.818391000000	0.359452000000	7.038841000000
C	-7.032060000000	-1.249232000000	-0.244055000000
H	-7.233652000000	-0.754510000000	0.713884000000
H	-7.403728000000	-0.569262000000	-1.020399000000
C	-7.818197000000	-2.547524000000	-0.316554000000
C	-8.835620000000	-2.724441000000	-1.259156000000
C	-7.552049000000	-3.598823000000	0.571563000000
C	-9.578478000000	-3.903348000000	-1.315072000000
C	-8.271896000000	-4.787773000000	0.521007000000
C	-9.293967000000	-4.941106000000	-0.424174000000
H	-10.360077000000	-4.021546000000	-2.054570000000
H	-8.054778000000	-5.587253000000	1.217797000000
N	-10.040525000000	-6.156502000000	-0.478454000000
C	-10.467965000000	-6.899346000000	0.643485000000
C	-10.463655000000	-6.799355000000	-1.661945000000
C	-11.209017000000	-8.085920000000	0.103594000000
C	-11.204407000000	-8.029370000000	-1.229615000000
H	-11.647937000000	-8.823684000000	0.761906000000
H	-11.635942000000	-8.710246000000	-1.951077000000
O	-10.269014000000	-6.625796000000	1.807190000000
O	-10.265539000000	-6.423191000000	-2.796904000000
H	-9.062988000000	-1.925132000000	-1.959606000000
H	-6.759724000000	-3.491617000000	1.307103000000
H	-5.654754000000	-2.395720000000	-2.315554000000
H	-5.040604000000	-0.481675000000	1.478570000000

$P2_{endo\text{-}BISM2}$

C	2.359986000000	-0.434724000000	-1.828971000000
C	1.727027000000	0.677737000000	-0.968523000000
C	1.973828000000	0.132594000000	0.477279000000
C	2.614922000000	-1.249128000000	0.178571000000
O	3.404267000000	-0.980389000000	-0.999404000000
H	2.775810000000	-0.137144000000	-2.791388000000
H	0.666163000000	0.807205000000	-1.193776000000
H	1.051528000000	0.031015000000	1.052388000000
C	3.385320000000	-2.026248000000	1.253721000000
H	3.842446000000	-2.888861000000	0.758585000000
H	2.636345000000	-2.419151000000	1.946380000000
C	-0.093621000000	-1.363836000000	-2.067231000000
C	1.387379000000	-1.656497000000	-1.886863000000
C	1.521103000000	-2.199175000000	-0.456076000000
C	0.125306000000	-2.148899000000	0.135117000000
H	1.714059000000	-2.354720000000	-2.660803000000
H	1.878703000000	-3.231957000000	-0.412265000000
N	-0.753207000000	-1.671196000000	-0.855237000000
O	-0.631726000000	-0.935304000000	-3.064481000000

O	-0.186125000000	-2.457901000000	1.267436000000
C	2.367751000000	2.122303000000	-0.881953000000
C	3.881087000000	1.981700000000	-0.789394000000
C	4.129773000000	1.509132000000	0.433477000000
C	2.773222000000	1.353123000000	1.093722000000
O	2.024714000000	2.425214000000	0.486049000000
H	4.575387000000	2.121841000000	-1.606594000000
H	5.068220000000	1.188611000000	0.865162000000
C	1.755621000000	3.119410000000	-1.867313000000
H	2.108386000000	2.865717000000	-2.871328000000
H	0.672450000000	2.971588000000	-1.877607000000
H	2.733079000000	1.428639000000	2.177968000000
N	2.010228000000	4.528962000000	-1.610936000000
C	3.010853000000	5.322620000000	-2.178064000000
C	1.161391000000	5.330575000000	-0.849565000000
C	2.784411000000	6.617298000000	-1.759404000000
C	1.635075000000	6.622161000000	-0.925645000000
H	3.379792000000	7.474584000000	-2.040548000000
H	1.198726000000	7.482961000000	-0.439933000000
C	-2.159988000000	-1.484536000000	-0.646440000000
C	-2.613886000000	-0.865110000000	0.515488000000
C	-3.069826000000	-1.908237000000	-1.608733000000
C	-3.980807000000	-0.691622000000	0.746036000000
H	-1.894444000000	-0.528669000000	1.247800000000
C	-4.443758000000	-1.732368000000	-1.414936000000
H	-2.713543000000	-2.356348000000	-2.528230000000
C	-4.916326000000	-1.149643000000	-0.217208000000
C	4.101739000000	4.807773000000	-3.075848000000
H	3.847310000000	3.809439000000	-3.449547000000
H	4.150310000000	5.446608000000	-3.967842000000
C	5.516639000000	4.750045000000	-2.447721000000
H	5.472737000000	4.181710000000	-1.512465000000
H	6.167464000000	4.187432000000	-3.130841000000
C	6.144159000000	6.119037000000	-2.174542000000
H	7.156574000000	6.009816000000	-1.772016000000
H	6.213482000000	6.715275000000	-3.092282000000
H	5.555240000000	6.685050000000	-1.447807000000
C	-0.063655000000	4.790891000000	-0.166404000000
H	-0.788193000000	4.428002000000	-0.913645000000
H	0.190947000000	3.921891000000	0.448564000000
C	-0.760842000000	5.838474000000	0.728760000000
H	-0.063490000000	6.141948000000	1.516071000000
H	-0.968654000000	6.743588000000	0.145771000000
C	-2.015208000000	5.340417000000	1.383185000000
C	-2.201143000000	4.789947000000	2.632730000000
N	-3.254400000000	5.342674000000	0.748875000000
C	-3.580060000000	4.461687000000	2.770671000000
H	-1.421759000000	4.640710000000	3.367728000000
C	-4.219208000000	4.817233000000	1.601255000000
H	-4.053563000000	4.019044000000	3.635264000000
C	-3.533622000000	5.946247000000	-0.541438000000
H	-2.657129000000	5.832073000000	-1.187765000000
H	-4.342989000000	5.392450000000	-1.029591000000
C	-3.904095000000	7.396702000000	-0.428625000000
C	-3.967041000000	8.277112000000	0.609624000000
O	-4.252487000000	8.017443000000	-1.596225000000

C	-4.382904000000	9.531797000000	0.047286000000
H	-3.742935000000	8.056510000000	1.642641000000
C	-4.540066000000	9.318571000000	-1.285468000000
H	-4.540876000000	10.462884000000	0.572009000000
H	-4.834358000000	9.944079000000	-2.113441000000
C	-5.664478000000	4.721460000000	1.203433000000
H	-6.036116000000	5.712394000000	0.906715000000
H	-5.771177000000	4.086113000000	0.310847000000
C	-6.561371000000	4.166800000000	2.314214000000
H	-6.519260000000	4.799058000000	3.206270000000
H	-7.602285000000	4.119695000000	1.981492000000
H	-6.254161000000	3.157383000000	2.605957000000
N	4.405201000000	-1.335703000000	2.019775000000
C	4.210944000000	-0.772861000000	3.281352000000
C	5.740632000000	-1.197385000000	1.646285000000
C	5.419991000000	-0.248168000000	3.682380000000
C	6.377090000000	-0.515724000000	2.662093000000
H	5.605965000000	0.257537000000	4.618768000000
H	7.423460000000	-0.247037000000	2.680096000000
C	2.901105000000	-0.867758000000	4.013944000000
H	2.655020000000	-1.924201000000	4.204669000000
H	2.074279000000	-0.488956000000	3.396604000000
C	6.279863000000	-1.762464000000	0.362902000000
H	6.146792000000	-2.855898000000	0.349296000000
H	5.709197000000	-1.388463000000	-0.493526000000
C	7.774420000000	-1.439578000000	0.144016000000
H	8.357886000000	-1.790552000000	1.003000000000
H	7.895860000000	-0.351400000000	0.121460000000
C	8.328421000000	-1.992375000000	-1.135736000000
C	8.414424000000	-1.404706000000	-2.379794000000
N	8.804585000000	-3.294869000000	-1.260491000000
C	8.949554000000	-2.363683000000	-3.281723000000
H	8.108236000000	-0.393263000000	-2.610826000000
C	9.191454000000	-3.525050000000	-2.578544000000
H	9.136491000000	-2.229557000000	-4.338686000000
C	8.949970000000	-4.229531000000	-0.159148000000
H	8.063372000000	-4.175717000000	0.483503000000
H	8.969000000000	-5.247424000000	-0.561202000000
C	10.181969000000	-3.984710000000	0.664057000000
C	11.194420000000	-3.073661000000	0.615152000000
O	10.366340000000	-4.838348000000	1.716605000000
C	12.068108000000	-3.384339000000	1.712358000000
H	11.300674000000	-2.281582000000	-0.110904000000
C	11.521306000000	-4.456342000000	2.343091000000
H	12.978750000000	-2.872268000000	1.987376000000
H	11.803590000000	-5.042229000000	3.203550000000
C	9.767980000000	-4.820111000000	-3.072200000000
H	9.603237000000	-4.850413000000	-4.154761000000
H	9.207253000000	-5.673901000000	-2.667131000000
C	11.269922000000	-5.016387000000	-2.790582000000
H	11.485345000000	-5.010651000000	-1.718435000000
H	11.620772000000	-5.968222000000	-3.203655000000
H	11.852476000000	-4.210547000000	-3.247488000000
C	2.892683000000	-0.119314000000	5.354173000000
H	3.690702000000	-0.516493000000	5.992705000000
H	3.135006000000	0.936388000000	5.180284000000

C	1.549372000000	-0.229775000000	6.079975000000
H	0.735281000000	0.189909000000	5.478444000000
H	1.297077000000	-1.274557000000	6.293260000000
H	1.569572000000	0.308401000000	7.032706000000
C	-6.413973000000	-0.930848000000	0.014172000000
H	-6.560869000000	-0.563603000000	1.033219000000
H	-6.721104000000	-0.100274000000	-0.628316000000
C	-7.387884000000	-2.091387000000	-0.193779000000
C	-8.518550000000	-1.910867000000	-1.022574000000
C	-7.225022000000	-3.319941000000	0.492279000000
C	-9.434309000000	-2.953520000000	-1.193456000000
C	-8.142161000000	-4.353653000000	0.282953000000
C	-9.239817000000	-4.176190000000	-0.557830000000
H	-10.294552000000	-2.811591000000	-1.835821000000
H	-8.022888000000	-5.296471000000	0.796973000000
N	-10.171107000000	-5.241804000000	-0.753525000000
C	-10.643745000000	-6.105920000000	0.256491000000
C	-10.758545000000	-5.588584000000	-1.988533000000
C	-11.593595000000	-7.054393000000	-0.413222000000
C	-11.658950000000	-6.756022000000	-1.712168000000
H	-12.104107000000	-7.828477000000	0.144067000000
H	-12.234481000000	-7.223492000000	-2.499968000000
O	-10.337683000000	-6.082188000000	1.429237000000
O	-10.569603000000	-5.044740000000	-3.055211000000
C	-8.810938000000	-0.602989000000	-1.730341000000
H	-8.919723000000	0.230050000000	-1.026609000000
H	-8.023585000000	-0.323622000000	-2.439498000000
H	-9.743029000000	-0.676840000000	-2.295619000000
C	-5.367133000000	-2.173068000000	-2.529499000000
H	-5.963115000000	-3.047738000000	-2.254488000000
H	-6.071698000000	-1.383150000000	-2.807473000000
H	-4.789622000000	-2.424988000000	-3.422512000000
C	-6.075993000000	-3.523115000000	1.469812000000
H	-5.124937000000	-3.466254000000	0.929293000000
H	-6.061810000000	-2.670746000000	2.161495000000
C	-4.435895000000	0.003929000000	2.025368000000
H	-5.049423000000	0.874117000000	1.756829000000
H	-5.107046000000	-0.667003000000	2.577704000000
C	-6.098976000000	-4.812737000000	2.295550000000
H	-7.022835000000	-4.912166000000	2.874224000000
H	-5.999131000000	-5.703150000000	1.666268000000
H	-5.260386000000	-4.816357000000	2.998156000000
C	-3.336114000000	0.473257000000	2.982375000000
H	-2.693378000000	1.231602000000	2.525357000000
H	-3.790017000000	0.926573000000	3.868514000000
H	-2.708762000000	-0.357639000000	3.320833000000

R1$_{exo\text{-}BISM1}$

C	4.136432000000	-1.654594000000	2.584918000000
C	5.452843000000	-1.885960000000	2.841398000000
C	6.171736000000	-1.385844000000	1.707218000000
C	5.243034000000	-0.881758000000	0.841376000000
O	3.990453000000	-1.047873000000	1.370295000000
H	3.224275000000	-1.840202000000	3.130057000000
H	5.863158000000	-2.345934000000	3.728705000000
H	7.242104000000	-1.388955000000	1.558192000000

C	5.344755000000	-0.228486000000	-0.504768000000
H	4.797114000000	-0.816232000000	-1.247816000000
H	6.395537000000	-0.251588000000	-0.803245000000
C	3.413537000000	-3.564636000000	-1.124944000000
C	4.446809000000	-4.133896000000	-0.201298000000
C	3.845938000000	-4.545130000000	0.918608000000
C	2.373676000000	-4.281294000000	0.805316000000
H	5.494741000000	-4.157386000000	-0.466721000000
H	4.274275000000	-4.995377000000	1.803152000000
N	2.178142000000	-3.710432000000	-0.469845000000
O	3.591436000000	-3.059617000000	-2.215518000000
O	1.513111000000	-4.502755000000	1.628843000000
C	0.913021000000	-3.329823000000	-1.011516000000
C	0.499964000000	-3.854765000000	-2.239253000000
C	0.086628000000	-2.446821000000	-0.316050000000
C	-0.738024000000	-3.492431000000	-2.761060000000
H	1.144326000000	-4.541749000000	-2.776722000000
C	-1.153783000000	-2.095063000000	-0.848623000000
H	0.409073000000	-2.024782000000	0.628291000000
C	-1.586252000000	-2.610643000000	-2.076138000000
H	-1.053208000000	-3.907174000000	-3.715259000000
H	-1.783859000000	-1.406532000000	-0.296125000000
N	4.861930000000	1.147758000000	-0.564162000000
C	5.618936000000	2.294684000000	-0.320707000000
C	3.564498000000	1.511203000000	-0.914670000000
C	4.786103000000	3.375036000000	-0.522757000000
C	3.502775000000	2.887547000000	-0.889038000000
H	5.081102000000	4.410767000000	-0.421290000000
H	2.626867000000	3.480523000000	-1.109578000000
C	7.064648000000	2.285958000000	0.084103000000
H	7.521727000000	3.199631000000	-0.315628000000
H	7.597198000000	1.457443000000	-0.401579000000
C	7.339709000000	2.230878000000	1.606664000000
H	8.422012000000	2.114930000000	1.753765000000
H	6.870494000000	1.335287000000	2.028578000000
C	6.852606000000	3.468975000000	2.362826000000
H	7.057327000000	3.381776000000	3.434949000000
H	7.352697000000	4.375514000000	2.001291000000
H	5.775549000000	3.609554000000	2.233226000000
C	2.533260000000	0.493649000000	-1.317385000000
H	2.511824000000	-0.337819000000	-0.605196000000
H	2.808185000000	0.047212000000	-2.283815000000
C	1.107425000000	1.052291000000	-1.451719000000
H	0.521519000000	0.311489000000	-2.013943000000
H	1.130065000000	1.954582000000	-2.077342000000
C	0.424825000000	1.327195000000	-0.140930000000
C	0.819844000000	1.094544000000	1.158636000000
N	-0.867810000000	1.841361000000	-0.089616000000
C	-0.249028000000	1.482579000000	2.018728000000
H	1.781229000000	0.702839000000	1.457851000000
C	-1.283087000000	1.947138000000	1.235321000000
H	-0.257789000000	1.425977000000	3.097905000000
C	-1.614119000000	2.328367000000	-1.232822000000
H	-1.390793000000	1.701302000000	-2.103195000000
H	-2.684777000000	2.207707000000	-1.036401000000
C	-1.306105000000	3.763641000000	-1.547991000000

C	-0.503956000000	4.703950000000	-0.973624000000
O	-1.947111000000	4.289890000000	-2.635714000000
C	-0.657508000000	5.896752000000	-1.759632000000
H	0.115077000000	4.560482000000	-0.100433000000
C	-1.538381000000	5.591480000000	-2.747982000000
H	-0.172180000000	6.849228000000	-1.603143000000
H	-1.961489000000	6.146352000000	-3.570513000000
C	-2.637940000000	2.481045000000	1.603366000000
H	-3.430080000000	1.879388000000	1.136625000000
H	-2.762858000000	3.496507000000	1.200255000000
C	-2.886541000000	2.512780000000	3.113989000000
H	-3.894667000000	2.881495000000	3.321545000000
H	-2.797064000000	1.511928000000	3.548362000000
H	-2.168746000000	3.164153000000	3.622706000000
C	-2.915186000000	-2.209768000000	-2.707118000000
H	-2.730016000000	-1.420982000000	-3.448136000000
H	-3.294988000000	-3.065922000000	-3.279493000000
C	-3.986780000000	-1.733223000000	-1.748318000000
C	-4.484734000000	-0.427827000000	-1.815018000000
C	-4.513973000000	-2.589686000000	-0.771336000000
C	-5.474682000000	0.019515000000	-0.940246000000
H	-4.102496000000	0.252942000000	-2.571071000000
C	-5.488380000000	-2.155949000000	0.121889000000
H	-4.143607000000	-3.608155000000	-0.695745000000
C	-5.970549000000	-0.844290000000	0.039237000000
H	-5.848699000000	1.033423000000	-1.009129000000
H	-5.881897000000	-2.830834000000	0.871554000000
N	-6.963391000000	-0.387759000000	0.956719000000
C	-8.082932000000	-1.135724000000	1.383572000000
C	-6.965437000000	0.872568000000	1.588391000000
C	-8.828824000000	-0.259732000000	2.345369000000
C	-8.181051000000	0.900979000000	2.465497000000
H	-9.740255000000	-0.593855000000	2.822783000000
H	-8.420871000000	1.765993000000	3.069246000000
O	-6.144401000000	1.753752000000	1.443535000000
O	-8.370204000000	-2.260136000000	1.036301000000

R1$_{exo\text{-}BISM2}$

C	4.386057000000	-4.013519000000	1.079149000000
C	5.372427000000	-4.938790000000	1.215400000000
C	6.437365000000	-4.513031000000	0.353419000000
C	6.021064000000	-3.359278000000	-0.243902000000
O	4.760819000000	-3.047320000000	0.189700000000
H	3.409558000000	-3.890668000000	1.520308000000
H	5.352363000000	-5.811462000000	1.851747000000
H	7.388556000000	-5.002106000000	0.198769000000
C	6.633581000000	-2.468094000000	-1.280558000000
H	6.030439000000	-2.495506000000	-2.194064000000
H	7.609339000000	-2.885092000000	-1.540251000000
C	-4.318635000000	-6.848729000000	-0.269961000000
C	-4.157012000000	-7.933829000000	0.753293000000
C	-3.874046000000	-7.377412000000	1.932588000000
C	-3.827396000000	-5.887013000000	1.768026000000
H	-4.268538000000	-8.977466000000	0.490990000000
H	-3.696112000000	-7.844798000000	2.891914000000
N	-4.104724000000	-5.630942000000	0.408633000000

O	-4.582862000000	-6.984314000000	-1.445326000000
O	-3.597065000000	-5.062297000000	2.625926000000
C	-4.162489000000	-4.333361000000	-0.187393000000
C	-5.153325000000	-4.034090000000	-1.117523000000
C	-3.214064000000	-3.369353000000	0.150610000000
C	-5.215562000000	-2.765904000000	-1.703216000000
H	-5.868919000000	-4.793975000000	-1.405857000000
C	-3.260463000000	-2.088959000000	-0.408005000000
H	-2.438339000000	-3.622067000000	0.858966000000
C	-4.288758000000	-1.766873000000	-1.327685000000
N	6.799181000000	-1.069728000000	-0.897357000000
C	7.928447000000	-0.514095000000	-0.295910000000
C	5.858932000000	-0.070953000000	-1.135344000000
C	7.687270000000	0.836784000000	-0.159968000000
C	6.394802000000	1.114361000000	-0.680595000000
H	8.379418000000	1.548233000000	0.269902000000
H	5.906853000000	2.077711000000	-0.715148000000
C	9.143452000000	-1.297724000000	0.107584000000
H	9.997136000000	-0.609553000000	0.079407000000
H	9.369197000000	-2.073270000000	-0.637018000000
C	9.089649000000	-1.952878000000	1.508904000000
H	9.987543000000	-2.573696000000	1.629607000000
H	8.232338000000	-2.633159000000	1.559387000000
C	9.010521000000	-0.941328000000	2.654518000000
H	8.961908000000	-1.447457000000	3.624216000000
H	9.889628000000	-0.285816000000	2.664951000000
H	8.124736000000	-0.306685000000	2.558039000000
C	4.562574000000	-0.347228000000	-1.847079000000
H	4.074347000000	-1.234677000000	-1.429239000000
H	4.763444000000	-0.581566000000	-2.903819000000
C	3.563075000000	0.821555000000	-1.818853000000
H	2.793647000000	0.613323000000	-2.577543000000
H	4.071132000000	1.734377000000	-2.157152000000
C	2.908493000000	1.042448000000	-0.484073000000
C	2.949953000000	0.308497000000	0.680068000000
N	2.040741000000	2.109813000000	-0.266497000000
C	2.099183000000	0.945448000000	1.630250000000
H	3.539782000000	-0.582955000000	0.836371000000
C	1.547920000000	2.060000000000	1.033708000000
H	1.917495000000	0.625014000000	2.646212000000
C	1.787952000000	3.182906000000	-1.207864000000
H	1.782464000000	2.779568000000	-2.226680000000
H	0.782457000000	3.580574000000	-1.031217000000
C	2.800712000000	4.286891000000	-1.104732000000
C	3.854690000000	4.525098000000	-0.273828000000
O	2.674572000000	5.303260000000	-2.011004000000
C	4.422425000000	5.776123000000	-0.694314000000
H	4.180495000000	3.887927000000	0.534939000000
C	3.672323000000	6.201455000000	-1.744297000000
H	5.275851000000	6.282578000000	-0.267245000000
H	3.706047000000	7.068440000000	-2.385118000000
C	0.598493000000	3.095295000000	1.565001000000
H	-0.329714000000	3.109915000000	0.977504000000
H	1.033947000000	4.097813000000	1.448089000000
C	0.231424000000	2.884138000000	3.036357000000
H	-0.471799000000	3.656122000000	3.359020000000

H	-0.242593000000	1.909377000000	3.189330000000
H	1.116816000000	2.931015000000	3.678466000000
C	-4.363209000000	-0.388790000000	-1.986424000000
H	-3.512601000000	-0.307091000000	-2.669185000000
H	-5.243895000000	-0.362880000000	-2.634365000000
C	-4.409920000000	0.867039000000	-1.112560000000
C	-3.554139000000	1.959360000000	-1.392037000000
C	-5.354312000000	0.991629000000	-0.066179000000
C	-3.633385000000	3.124100000000	-0.620268000000
C	-5.396483000000	2.158643000000	0.701982000000
C	-4.539911000000	3.223423000000	0.431379000000
H	-2.973249000000	3.954927000000	-0.838984000000
H	-6.121322000000	2.242658000000	1.502290000000
N	-4.598882000000	4.412354000000	1.221468000000
C	-5.786089000000	5.033100000000	1.668577000000
C	-3.483409000000	5.140695000000	1.678028000000
C	-5.359283000000	6.233486000000	2.460252000000
C	-4.026341000000	6.294597000000	2.467211000000
H	-6.083326000000	6.893696000000	2.918725000000
H	-3.370289000000	7.016293000000	2.935290000000
O	-2.316095000000	4.883907000000	1.468138000000
O	-6.918878000000	4.662051000000	1.451790000000
C	-6.292476000000	-2.531365000000	-2.743746000000
H	-6.990427000000	-1.736231000000	-2.457905000000
H	-5.869485000000	-2.252955000000	-3.715597000000
H	-6.879428000000	-3.440563000000	-2.894302000000
C	-6.351960000000	-0.096476000000	0.264449000000
H	-6.898710000000	-0.427012000000	-0.624238000000
H	-5.872430000000	-0.981865000000	0.691143000000
H	-7.087834000000	0.269862000000	0.984619000000
C	-2.188549000000	-1.071872000000	-0.040435000000
H	-1.804460000000	-0.624884000000	-0.966261000000
H	-2.652940000000	-0.241446000000	0.503202000000
C	-1.000795000000	-1.591156000000	0.775208000000
H	-1.310691000000	-1.949165000000	1.762547000000
H	-0.486749000000	-2.412806000000	0.264767000000
H	-0.272634000000	-0.789533000000	0.928093000000
C	-2.562447000000	1.971890000000	-2.544769000000
H	-1.798875000000	2.728193000000	-2.332117000000
H	-2.023758000000	1.020471000000	-2.611738000000
C	-3.206907000000	2.293392000000	-3.905147000000
H	-3.710454000000	3.264701000000	-3.874855000000
H	-2.450598000000	2.327243000000	-4.696317000000
H	-3.953411000000	1.544075000000	-4.185888000000

TS1$_{exo\text{-}BISM1}$

C	5.182568000000	-0.634454000000	1.630245000000
C	6.467433000000	-0.094432000000	1.297114000000
C	6.386637000000	0.266913000000	-0.024586000000
C	5.044250000000	0.033161000000	-0.435377000000
O	4.287413000000	-0.172657000000	0.677791000000
H	4.758966000000	-0.742007000000	2.621474000000
H	7.348301000000	-0.145543000000	1.921908000000
H	7.185674000000	0.586335000000	-0.679150000000
C	4.345386000000	0.472257000000	-1.698039000000
H	3.723683000000	-0.340924000000	-2.078742000000

H	5.114858000000	0.648776000000	-2.453097000000
C	4.047983000000	-2.700882000000	-1.022753000000
C	5.356783000000	-2.298109000000	-0.486313000000
C	5.334884000000	-2.450960000000	0.921336000000
C	3.978985000000	-3.015858000000	1.277706000000
H	6.227801000000	-2.341241000000	-1.123792000000
H	6.169400000000	-2.831461000000	1.499222000000
N	3.238537000000	-3.076594000000	0.086192000000
O	3.672845000000	-2.707975000000	-2.184024000000
O	3.585993000000	-3.330905000000	2.381971000000
C	1.865604000000	-3.453116000000	0.002357000000
C	1.436056000000	-4.308615000000	-1.015306000000
C	0.944988000000	-2.967872000000	0.940086000000
C	0.091748000000	-4.672359000000	-1.089299000000
H	2.144162000000	-4.672991000000	-1.748536000000
C	-0.390358000000	-3.350211000000	0.857266000000
H	1.270705000000	-2.297793000000	1.725951000000
C	-0.840387000000	-4.209431000000	-0.155286000000
H	-0.231446000000	-5.339079000000	-1.884634000000
H	-1.095092000000	-2.963547000000	1.588076000000
N	3.523997000000	1.666771000000	-1.552640000000
C	3.931845000000	2.985377000000	-1.771902000000
C	2.161146000000	1.642749000000	-1.260707000000
C	2.815872000000	3.779535000000	-1.616172000000
C	1.712804000000	2.944225000000	-1.292126000000
H	2.801411000000	4.854940000000	-1.730833000000
H	0.698390000000	3.261179000000	-1.098120000000
C	5.338961000000	3.395431000000	-2.097379000000
H	5.287300000000	4.247527000000	-2.786508000000
H	5.853981000000	2.602772000000	-2.655425000000
C	6.214847000000	3.804111000000	-0.887173000000
H	7.245735000000	3.940349000000	-1.241091000000
H	6.239447000000	2.982664000000	-0.161898000000
C	5.744039000000	5.082229000000	-0.189633000000
H	6.389531000000	5.327474000000	0.660083000000
H	5.760307000000	5.936273000000	-0.877114000000
H	4.721384000000	4.972237000000	0.182364000000
C	1.415711000000	0.354502000000	-1.046290000000
H	1.952464000000	-0.287600000000	-0.340417000000
H	1.372699000000	-0.211354000000	-1.988048000000
C	-0.025969000000	0.526562000000	-0.542141000000
H	-0.533457000000	-0.438927000000	-0.676614000000
H	-0.553592000000	1.235678000000	-1.194037000000
C	-0.133006000000	0.949666000000	0.896442000000
C	0.827490000000	1.127829000000	1.868693000000
N	-1.367567000000	1.190936000000	1.490261000000
C	0.165907000000	1.494436000000	3.076330000000
H	1.892773000000	1.025507000000	1.720984000000
C	-1.188967000000	1.534118000000	2.826296000000
H	0.634819000000	1.705067000000	4.026929000000
C	-2.640034000000	1.182690000000	0.793759000000
H	-2.632819000000	0.395100000000	0.033181000000
H	-3.437663000000	0.914081000000	1.493518000000
C	-2.944572000000	2.505004000000	0.150031000000
C	-2.340421000000	3.726415000000	0.170890000000
O	-4.067195000000	2.550592000000	-0.633981000000

C	-3.140863000000	4.588704000000	-0.653983000000
H	-1.437623000000	3.975737000000	0.708468000000
C	-4.169047000000	3.830186000000	-1.113931000000
H	-2.964429000000	5.631877000000	-0.873089000000
H	-5.011075000000	4.023050000000	-1.759678000000
C	-2.346875000000	1.859824000000	3.726022000000
H	-3.030522000000	1.000406000000	3.791860000000
H	-2.942761000000	2.674709000000	3.290861000000
C	-1.915639000000	2.256806000000	5.141125000000
H	-2.788394000000	2.488077000000	5.758682000000
H	-1.362968000000	1.446969000000	5.627451000000
H	-1.269036000000	3.139635000000	5.123107000000
C	-2.296879000000	-4.640921000000	-0.222596000000
H	-2.422468000000	-5.322541000000	-1.072306000000
H	-2.544280000000	-5.222782000000	0.673665000000
C	-3.287556000000	-3.496154000000	-0.347306000000
C	-4.232268000000	-3.244968000000	0.652675000000
C	-3.285459000000	-2.664054000000	-1.476171000000
C	-5.154563000000	-2.205103000000	0.537476000000
H	-4.258318000000	-3.877426000000	1.536031000000
C	-4.189828000000	-1.613765000000	-1.603138000000
H	-2.558705000000	-2.835053000000	-2.265578000000
C	-5.131260000000	-1.383160000000	-0.591541000000
H	-5.877440000000	-2.025652000000	1.323555000000
H	-4.178928000000	-0.984371000000	-2.484238000000
N	-6.059753000000	-0.306531000000	-0.708696000000
C	-6.735327000000	0.061238000000	-1.891962000000
C	-6.407066000000	0.577928000000	0.331144000000
C	-7.579383000000	1.249333000000	-1.538933000000
C	-7.387139000000	1.549924000000	-0.252877000000
H	-8.214888000000	1.727800000000	-2.272140000000
H	-7.820007000000	2.341422000000	0.344033000000
O	-5.993944000000	0.545439000000	1.471747000000
O	-6.643704000000	-0.482969000000	-2.970576000000

TS1$_{exo\text{-}BISM2}$

C	5.368437000000	-0.815547000000	1.722761000000
C	6.692253000000	-0.351671000000	1.432199000000
C	6.667756000000	0.039760000000	0.116848000000
C	5.324397000000	-0.103134000000	-0.331504000000
O	4.527981000000	-0.280169000000	0.760075000000
H	4.914123000000	-0.921332000000	2.700399000000
H	7.552274000000	-0.471295000000	2.076381000000
H	7.502042000000	0.318535000000	-0.511642000000
C	4.685365000000	0.404949000000	-1.599180000000
H	3.977239000000	-0.334796000000	-1.977728000000
H	5.472659000000	0.486704000000	-2.351835000000
C	4.171329000000	-2.746053000000	-1.007837000000
C	5.491386000000	-2.437772000000	-0.433078000000
C	5.427587000000	-2.627312000000	0.968426000000
C	4.031123000000	-3.112846000000	1.280790000000
H	6.372427000000	-2.523094000000	-1.052481000000
H	6.224390000000	-3.070645000000	1.554505000000
N	3.316655000000	-3.098609000000	0.072804000000
O	3.823564000000	-2.699794000000	-2.176389000000
O	3.594232000000	-3.426486000000	2.369395000000

C	1.917363000000	-3.349937000000	-0.051425000000
C	1.440270000000	-4.155999000000	-1.080759000000
C	1.024159000000	-2.800169000000	0.867022000000
C	0.067634000000	-4.379382000000	-1.231712000000
H	2.137864000000	-4.602208000000	-1.778617000000
C	-0.351108000000	-3.020553000000	0.751634000000
H	1.410412000000	-2.212619000000	1.687615000000
C	-0.848888000000	-3.778479000000	-0.338126000000
N	4.004704000000	1.687712000000	-1.468661000000
C	4.556364000000	2.947002000000	-1.719731000000
C	2.645562000000	1.822570000000	-1.188597000000
C	3.534357000000	3.863662000000	-1.594541000000
C	2.344143000000	3.164304000000	-1.258147000000
H	3.639759000000	4.930718000000	-1.736091000000
H	1.370269000000	3.596418000000	-1.079713000000
C	6.003029000000	3.192055000000	-2.037961000000
H	6.052181000000	4.019419000000	-2.756803000000
H	6.436759000000	2.329322000000	-2.559707000000
C	6.902358000000	3.547742000000	-0.827982000000
H	7.946843000000	3.555153000000	-1.167968000000
H	6.824610000000	2.757251000000	-0.072656000000
C	6.567832000000	4.896018000000	-0.186102000000
H	7.225918000000	5.100764000000	0.664672000000
H	6.687572000000	5.715960000000	-0.904170000000
H	5.534822000000	4.914109000000	0.172801000000
C	1.759853000000	0.632159000000	-0.943735000000
H	2.203609000000	-0.032432000000	-0.194846000000
H	1.681787000000	0.032894000000	-1.862553000000
C	0.330162000000	0.980087000000	-0.498317000000
H	-0.270899000000	0.066167000000	-0.602859000000
H	-0.101362000000	1.703407000000	-1.203384000000
C	0.222197000000	1.488206000000	0.912429000000
C	1.172847000000	1.651651000000	1.897109000000
N	-1.003494000000	1.854843000000	1.460363000000
C	0.515793000000	2.137544000000	3.064578000000
H	2.229741000000	1.458982000000	1.782859000000
C	-0.826907000000	2.262586000000	2.778255000000
H	0.979476000000	2.369150000000	4.012801000000
C	-2.260486000000	1.896924000000	0.736178000000
H	-2.294844000000	1.071503000000	0.017683000000
H	-3.088436000000	1.719803000000	1.430395000000
C	-2.459062000000	3.200699000000	0.018165000000
C	-1.767269000000	4.374327000000	-0.017030000000
O	-3.565565000000	3.284140000000	-0.784987000000
C	-2.492245000000	5.245498000000	-0.900261000000
H	-0.855996000000	4.587523000000	0.521825000000
C	-3.567554000000	4.539247000000	-1.335038000000
H	-2.237376000000	6.259542000000	-1.172428000000
H	-4.385735000000	4.756805000000	-2.003096000000
C	-1.975071000000	2.726042000000	3.628774000000
H	-2.721757000000	1.924299000000	3.728016000000
H	-2.498953000000	3.554807000000	3.131635000000
C	-1.543996000000	3.175092000000	5.028259000000
H	-2.409373000000	3.506760000000	5.609313000000
H	-1.063622000000	2.357689000000	5.574901000000
H	-0.832991000000	4.005364000000	4.974358000000

C	-2.351930000000	-4.012491000000	-0.520259000000
H	-2.502765000000	-4.626849000000	-1.412703000000
H	-2.695857000000	-4.633078000000	0.311133000000
C	-3.270060000000	-2.790938000000	-0.640273000000
C	-4.407212000000	-2.664336000000	0.199252000000
C	-3.034997000000	-1.808860000000	-1.630713000000
C	-5.223589000000	-1.534958000000	0.087903000000
C	-3.865407000000	-0.685283000000	-1.710691000000
C	-4.943733000000	-0.539830000000	-0.844706000000
H	-6.084662000000	-1.417049000000	0.731605000000
H	-3.678249000000	0.069815000000	-2.465670000000
N	-5.773895000000	0.619288000000	-0.917066000000
C	-6.343941000000	1.138809000000	-2.096453000000
C	-6.087654000000	1.448808000000	0.174095000000
C	-7.102092000000	2.366744000000	-1.684625000000
C	-6.952102000000	2.547264000000	-0.370447000000
H	-7.655602000000	2.957061000000	-2.402812000000
H	-7.346036000000	3.326322000000	0.268478000000
O	-5.725049000000	1.293684000000	1.321992000000
O	-6.235130000000	0.676343000000	-3.211013000000
C	-0.368687000000	-5.300125000000	-2.353841000000
H	-0.942499000000	-6.156377000000	-1.981782000000
H	-0.992456000000	-4.793361000000	-3.098030000000
H	0.503556000000	-5.695770000000	-2.879677000000
C	-1.927658000000	-1.931289000000	-2.653380000000
H	-0.933247000000	-1.934986000000	-2.200287000000
H	-2.020788000000	-2.860318000000	-3.226311000000
H	-1.974396000000	-1.104984000000	-3.366833000000
C	-4.776880000000	-3.753386000000	1.201734000000
H	-4.860890000000	-4.708359000000	0.666702000000
H	-3.950917000000	-3.889710000000	1.910506000000
C	-6.062784000000	-3.538783000000	2.005747000000
H	-6.012216000000	-2.637003000000	2.624038000000
H	-6.940999000000	-3.456350000000	1.357314000000
H	-6.225795000000	-4.388572000000	2.675231000000
C	-1.285649000000	-2.474269000000	1.821460000000
H	-1.974298000000	-1.750823000000	1.371672000000
H	-1.921209000000	-3.301610000000	2.163524000000
C	-0.627096000000	-1.834519000000	3.047637000000
H	0.053103000000	-2.529403000000	3.551126000000
H	-0.066029000000	-0.931289000000	2.790287000000
H	-1.398222000000	-1.543265000000	3.767419000000

P1$_{exo\text{-}BISM1}$

C	-1.359575000000	-0.351429000000	2.454641000000
C	-2.478063000000	0.542880000000	2.965780000000
C	-2.673475000000	1.453664000000	2.008299000000
C	-1.679049000000	1.116360000000	0.897270000000
O	-1.568562000000	-0.319442000000	1.033381000000
H	-1.293483000000	-1.374026000000	2.823086000000
H	-2.933055000000	0.482511000000	3.946426000000
H	-3.338190000000	2.306535000000	2.004247000000
C	-1.993301000000	1.531410000000	-0.548945000000
H	-1.686860000000	0.705695000000	-1.198149000000
H	-1.367709000000	2.380610000000	-0.822032000000
C	0.876321000000	1.321083000000	0.469550000000

C	-0.253335000000	1.541171000000	1.465955000000
C	-0.031710000000	0.480545000000	2.560565000000
C	1.169387000000	-0.331003000000	2.096977000000
H	-0.243042000000	2.581882000000	1.795235000000
H	0.150384000000	0.861094000000	3.567562000000
N	1.644168000000	0.223587000000	0.892850000000
O	1.088405000000	1.980140000000	-0.528098000000
O	1.635579000000	-1.299285000000	2.655717000000
C	2.762297000000	-0.294408000000	0.162644000000
C	3.713922000000	0.581824000000	-0.371278000000
C	2.904500000000	-1.672049000000	-0.012383000000
C	4.796871000000	0.072972000000	-1.080210000000
H	3.598060000000	1.650585000000	-0.242869000000
C	4.001110000000	-2.164819000000	-0.718836000000
H	2.175146000000	-2.351013000000	0.410443000000
C	4.959971000000	-1.306825000000	-1.266766000000
H	5.534012000000	0.759475000000	-1.486827000000
H	4.105400000000	-3.238687000000	-0.849006000000
N	-3.380236000000	1.878366000000	-0.806878000000
C	-3.870150000000	3.179052000000	-0.942335000000
C	-4.425384000000	0.970475000000	-0.962329000000
C	-5.226318000000	3.077246000000	-1.169991000000
C	-5.574237000000	1.699862000000	-1.181457000000
H	-5.891314000000	3.915231000000	-1.330513000000
H	-6.558784000000	1.284453000000	-1.339444000000
C	-3.017048000000	4.415379000000	-0.899206000000
H	-3.567687000000	5.197072000000	-1.435794000000
H	-2.091365000000	4.266347000000	-1.470713000000
C	-2.654556000000	4.960026000000	0.502220000000
H	-1.969945000000	5.808000000000	0.373824000000
H	-2.090094000000	4.201851000000	1.060210000000
C	-3.869664000000	5.402444000000	1.321091000000
H	-3.574355000000	5.753909000000	2.315089000000
H	-4.398666000000	6.222608000000	0.822279000000
H	-4.585499000000	4.583408000000	1.444445000000
C	-4.191531000000	-0.515432000000	-0.980595000000
H	-3.646849000000	-0.839543000000	-0.087726000000
H	-3.534227000000	-0.763282000000	-1.827510000000
C	-5.469222000000	-1.357514000000	-1.132528000000
H	-5.158872000000	-2.372967000000	-1.420563000000
H	-6.049864000000	-0.972630000000	-1.981492000000
C	-6.323542000000	-1.428473000000	0.102485000000
C	-6.101167000000	-0.989461000000	1.388643000000
N	-7.556769000000	-2.074749000000	0.097079000000
C	-7.224019000000	-1.364090000000	2.181220000000
H	-5.227370000000	-0.449631000000	1.723272000000
C	-8.117516000000	-2.028876000000	1.369783000000
H	-7.359616000000	-1.169316000000	3.235536000000
C	-8.216657000000	-2.602086000000	-1.081546000000
H	-7.463223000000	-2.991973000000	-1.774227000000
H	-8.834048000000	-3.459927000000	-0.792239000000
C	-9.061481000000	-1.572202000000	-1.774832000000
C	-9.388825000000	-0.278370000000	-1.497052000000
O	-9.663938000000	-1.972893000000	-2.934958000000
C	-10.253628000000	0.153986000000	-2.559732000000
H	-9.055742000000	0.289718000000	-0.641105000000

C	-10.386064000000	-0.906626000000	-3.398504000000
H	-10.709360000000	1.126510000000	-2.676634000000
H	-10.922219000000	-1.068964000000	-4.320235000000
C	-9.456650000000	-2.637945000000	1.672555000000
H	-9.437328000000	-3.718873000000	1.464918000000
H	-10.219537000000	-2.221205000000	0.999603000000
C	-9.900719000000	-2.425109000000	3.122969000000
H	-10.879705000000	-2.880954000000	3.296891000000
H	-9.189847000000	-2.873855000000	3.823587000000
H	-9.976222000000	-1.359122000000	3.358494000000
C	6.137663000000	-1.851051000000	-2.058028000000
H	6.047067000000	-1.535620000000	-3.104774000000
H	6.079313000000	-2.946347000000	-2.066068000000
C	7.498900000000	-1.422831000000	-1.536566000000
C	8.406839000000	-0.746409000000	-2.357005000000
C	7.885951000000	-1.708087000000	-0.219834000000
C	9.667827000000	-0.372648000000	-1.893295000000
H	8.132213000000	-0.512744000000	-3.382279000000
C	9.133383000000	-1.329807000000	0.265117000000
H	7.195339000000	-2.223093000000	0.442050000000
C	10.032119000000	-0.661984000000	-0.576114000000
H	10.355017000000	0.153260000000	-2.543674000000
H	9.416345000000	-1.562082000000	1.283881000000
N	11.316115000000	-0.275026000000	-0.086665000000
C	12.128589000000	-1.051417000000	0.767735000000
C	11.966424000000	0.942444000000	-0.382363000000
C	13.369924000000	-0.246366000000	1.012232000000
C	13.275093000000	0.907679000000	0.349049000000
H	14.168308000000	-0.618178000000	1.640456000000
H	13.974037000000	1.731513000000	0.293370000000
O	11.544917000000	1.831672000000	-1.089847000000
O	11.866007000000	-2.148013000000	1.211802000000

P1$_{exo\text{-}BISM2}$

C	-2.144408000000	-0.260825000000	2.575135000000
C	-3.301097000000	0.589013000000	3.077188000000
C	-3.479509000000	1.533737000000	2.149719000000
C	-2.436103000000	1.261912000000	1.065850000000
O	-2.300256000000	-0.175951000000	1.149144000000
H	-2.070022000000	-1.295918000000	2.905149000000
H	-3.791568000000	0.480176000000	4.036336000000
H	-4.161701000000	2.372709000000	2.154079000000
C	-2.704012000000	1.728926000000	-0.373843000000
H	-2.354226000000	0.937209000000	-1.043121000000
H	-2.087626000000	2.601419000000	-0.587953000000
C	0.128393000000	1.531725000000	0.744952000000
C	-1.042667000000	1.691461000000	1.705266000000
C	-0.840450000000	0.593217000000	2.765831000000
C	0.396164000000	-0.173809000000	2.319308000000
H	-1.066477000000	2.718450000000	2.074604000000
H	-0.706688000000	0.937189000000	3.793360000000
N	0.902886000000	0.435274000000	1.156296000000
O	0.361713000000	2.232922000000	-0.218823000000
O	0.862564000000	-1.151931000000	2.861284000000
C	2.059092000000	-0.035406000000	0.451476000000
C	3.017783000000	0.864952000000	0.000479000000

C	2.213563000000	-1.397669000000	0.205787000000
C	4.149898000000	0.414661000000	-0.686587000000
H	2.877215000000	1.927059000000	0.159237000000
C	3.341306000000	-1.878855000000	-0.463662000000
H	1.450981000000	-2.080450000000	0.551066000000
C	4.340162000000	-0.969548000000	-0.897562000000
N	-4.087702000000	2.056669000000	-0.672070000000
C	-4.602066000000	3.350897000000	-0.773466000000
C	-5.105068000000	1.133765000000	-0.903526000000
C	-5.946221000000	3.229433000000	-1.056418000000
C	-6.261637000000	1.846514000000	-1.136575000000
H	-6.623905000000	4.058802000000	-1.208465000000
H	-7.229695000000	1.416751000000	-1.348810000000
C	-3.779480000000	4.602285000000	-0.649167000000
H	-4.327342000000	5.393223000000	-1.174970000000
H	-2.830114000000	4.495870000000	-1.190511000000
C	-3.481580000000	5.097355000000	0.785429000000
H	-2.810567000000	5.962978000000	0.716931000000
H	-2.922878000000	4.328514000000	1.334478000000
C	-4.735753000000	5.483062000000	1.573579000000
H	-4.485572000000	5.800135000000	2.591251000000
H	-5.262484000000	6.312168000000	1.087274000000
H	-5.438552000000	4.646107000000	1.637130000000
C	-4.835679000000	-0.344503000000	-0.973739000000
H	-4.320084000000	-0.694255000000	-0.073313000000
H	-4.138554000000	-0.541259000000	-1.802025000000
C	-6.085649000000	-1.207074000000	-1.214411000000
H	-5.739986000000	-2.201737000000	-1.533214000000
H	-6.640790000000	-0.797758000000	-2.069001000000
C	-6.986857000000	-1.351667000000	-0.019731000000
C	-6.825996000000	-0.965918000000	1.292422000000
N	-8.203511000000	-2.023445000000	-0.103659000000
C	-7.970364000000	-1.399777000000	2.021470000000
H	-5.979113000000	-0.422630000000	1.685637000000
C	-8.815206000000	-2.046679000000	1.145992000000
H	-8.152178000000	-1.255341000000	3.076928000000
C	-8.803990000000	-2.510966000000	-1.330208000000
H	-8.015065000000	-2.853412000000	-2.008298000000
H	-9.412737000000	-3.393643000000	-1.104149000000
C	-9.643878000000	-1.468837000000	-2.011090000000
C	-10.012746000000	-0.196638000000	-1.688994000000
O	-10.189135000000	-1.828643000000	-3.212215000000
C	-10.843677000000	0.265343000000	-2.766121000000
H	-9.728008000000	0.338521000000	-0.795219000000
C	-10.916856000000	-0.758085000000	-3.656749000000
H	-11.317074000000	1.232112000000	-2.857863000000
H	-11.411135000000	-0.889332000000	-4.606418000000
C	-10.150775000000	-2.697268000000	1.367240000000
H	-10.098263000000	-3.767168000000	1.113313000000
H	-10.896032000000	-2.267248000000	0.682967000000
C	-10.656503000000	-2.559111000000	2.806458000000
H	-11.630810000000	-3.043037000000	2.920508000000
H	-9.963629000000	-3.023493000000	3.514870000000
H	-10.765795000000	-1.506588000000	3.085277000000
C	5.562653000000	-1.489994000000	-1.658799000000
H	5.409784000000	-1.253202000000	-2.715957000000

H	5.563664000000	-2.582321000000	-1.615190000000
C	6.960344000000	-1.021204000000	-1.252673000000
C	7.831132000000	-0.495618000000	-2.234125000000
C	7.431132000000	-1.178582000000	0.074014000000
C	9.123265000000	-0.095250000000	-1.880437000000
C	8.718554000000	-0.746619000000	0.404767000000
C	9.561166000000	-0.203579000000	-0.563913000000
H	9.783160000000	0.314199000000	-2.635257000000
H	9.084252000000	-0.856604000000	1.415472000000
N	10.876701000000	0.225907000000	-0.208607000000
C	11.749012000000	-0.458852000000	0.663267000000
C	11.500330000000	1.401020000000	-0.678284000000
C	13.003038000000	0.361598000000	0.732042000000
C	12.858665000000	1.438224000000	-0.042919000000
H	13.844940000000	0.054952000000	1.338319000000
H	13.549849000000	2.247756000000	-0.236115000000
O	11.026815000000	2.212271000000	-1.444397000000
O	11.523177000000	-1.501039000000	1.239847000000
C	7.435443000000	-0.367185000000	-3.691454000000
H	7.167928000000	-1.334470000000	-4.131671000000
H	6.578319000000	0.299823000000	-3.837113000000
H	8.264140000000	0.039684000000	-4.275862000000
C	5.121666000000	1.459731000000	-1.189352000000
H	6.072802000000	1.437385000000	-0.650104000000
H	5.353943000000	1.319776000000	-2.249587000000
H	4.693735000000	2.459180000000	-1.077816000000
C	6.561126000000	-1.830030000000	1.139782000000
H	5.673065000000	-1.212123000000	1.311210000000
H	6.176304000000	-2.775286000000	0.735091000000
C	7.229462000000	-2.119605000000	2.486985000000
H	8.114918000000	-2.753837000000	2.377272000000
H	7.535450000000	-1.200579000000	2.997422000000
H	6.525030000000	-2.636913000000	3.145047000000
C	3.464762000000	-3.375926000000	-0.731164000000
H	3.594949000000	-3.534406000000	-1.809826000000
H	4.389069000000	-3.748789000000	-0.270212000000
C	2.306576000000	-4.256200000000	-0.252081000000
H	2.169018000000	-4.195156000000	0.831939000000
H	1.360567000000	-3.980462000000	-0.728945000000
H	2.509180000000	-5.301923000000	-0.501947000000

$R2_{exo\text{-}BISM1}$

C	-1.476383000000	0.924279000000	-2.586883000000
C	-2.807216000000	1.381966000000	-3.160424000000
C	-3.737995000000	0.815709000000	-2.390784000000
C	-2.980971000000	0.022759000000	-1.322200000000
O	-1.772338000000	0.802851000000	-1.185323000000
H	-0.593947000000	1.539651000000	-2.751238000000
H	-2.932754000000	1.967271000000	-4.061817000000
H	-4.812584000000	0.832848000000	-2.506893000000
C	-3.668721000000	-0.239620000000	0.017643000000
H	-2.916386000000	-0.562465000000	0.739522000000
H	-4.353505000000	-1.080811000000	-0.106169000000
C	-1.604871000000	-2.188711000000	-1.173616000000
C	-2.391108000000	-1.244358000000	-2.076811000000
C	-1.326715000000	-0.588477000000	-2.982066000000

C	0.004062000000	-1.169966000000	-2.531706000000
H	-3.170172000000	-1.811587000000	-2.590548000000
H	-1.446978000000	-0.753779000000	-4.054625000000
N	-0.244999000000	-2.105477000000	-1.509043000000
O	-2.065160000000	-2.905316000000	-0.307406000000
O	1.096703000000	-0.880964000000	-2.965833000000
C	-0.101586000000	4.917923000000	-2.559678000000
C	-1.088964000000	5.383512000000	-3.378929000000
C	-2.334444000000	5.105072000000	-2.725267000000
C	-2.018336000000	4.484917000000	-1.556571000000
O	-0.663101000000	4.357768000000	-1.441465000000
H	-0.944857000000	5.868893000000	-4.333663000000
H	-3.328614000000	5.337187000000	-3.078334000000
H	-2.605227000000	4.075874000000	-0.748687000000
C	1.391022000000	4.882196000000	-2.675834000000
H	1.648329000000	5.094951000000	-3.717850000000
H	1.751623000000	3.873162000000	-2.462148000000
N	2.119155000000	5.807042000000	-1.808960000000
C	2.848651000000	5.464541000000	-0.671892000000
C	2.259374000000	7.170021000000	-2.056682000000
C	3.435171000000	6.621164000000	-0.203167000000
C	3.066853000000	7.687322000000	-1.065853000000
H	4.069527000000	6.685150000000	0.671001000000
H	3.368326000000	8.721112000000	-0.977849000000
C	1.624689000000	7.844700000000	-3.238970000000
H	1.920957000000	7.337010000000	-4.171189000000
H	0.530846000000	7.750081000000	-3.188823000000
C	1.987497000000	9.330363000000	-3.362969000000
H	1.681819000000	9.851519000000	-2.447736000000
H	3.077590000000	9.431831000000	-3.426204000000
C	1.334176000000	9.996943000000	-4.576444000000
H	0.241099000000	9.937557000000	-4.522933000000
H	1.605546000000	11.055310000000	-4.641960000000
H	1.646015000000	9.516601000000	-5.511103000000
C	2.916652000000	4.090142000000	-0.074076000000
H	2.886392000000	3.303844000000	-0.837484000000
H	3.898921000000	3.989765000000	0.401494000000
C	1.807561000000	3.797790000000	0.978372000000
H	1.806581000000	4.609393000000	1.715799000000
H	0.834962000000	3.840772000000	0.481857000000
C	1.935046000000	2.461383000000	1.645413000000
C	1.300349000000	1.269436000000	1.362195000000
N	2.823538000000	2.213257000000	2.688497000000
C	1.797762000000	0.280594000000	2.259136000000
H	0.555247000000	1.127481000000	0.590674000000
C	2.733420000000	0.880100000000	3.075198000000
H	1.506964000000	-0.759094000000	2.295121000000
C	3.595810000000	3.222516000000	3.389695000000
H	4.538204000000	2.778982000000	3.730756000000
H	3.871720000000	4.016326000000	2.688789000000
C	2.854284000000	3.810127000000	4.555448000000
C	1.630771000000	3.582662000000	5.110731000000
O	3.510258000000	4.792589000000	5.244348000000
C	1.519260000000	4.487361000000	6.221002000000
H	0.904018000000	2.859695000000	4.771467000000
C	2.679791000000	5.193075000000	6.255542000000

H	0.682721000000	4.592657000000	6.896490000000
H	3.061103000000	5.975045000000	6.893128000000
C	3.564124000000	0.316262000000	4.192415000000
H	4.634358000000	0.429008000000	3.960994000000
H	3.397926000000	0.894073000000	5.112499000000
C	3.273198000000	-1.161473000000	4.471370000000
H	2.227282000000	-1.309522000000	4.756783000000
H	3.467580000000	-1.776431000000	3.587040000000
H	3.902379000000	-1.530472000000	5.286538000000
C	0.782179000000	-2.866517000000	-0.862707000000
C	1.944937000000	-2.234139000000	-0.411644000000
C	0.622538000000	-4.242914000000	-0.695667000000
C	2.941560000000	-2.988135000000	0.200948000000
H	2.068033000000	-1.165776000000	-0.535894000000
C	1.625968000000	-4.981175000000	-0.069814000000
H	-0.279577000000	-4.730452000000	-1.045199000000
C	2.798769000000	-4.370264000000	0.386814000000
N	-4.404811000000	0.896508000000	0.551619000000
C	-5.750574000000	1.166651000000	0.295211000000
C	-3.846456000000	1.956112000000	1.264125000000
C	-6.030593000000	2.399304000000	0.841733000000
C	-4.843288000000	2.893995000000	1.446749000000
H	-7.001023000000	2.877404000000	0.829513000000
H	-4.736026000000	3.824452000000	1.985783000000
C	-2.411827000000	1.968967000000	1.718325000000
H	-2.120277000000	0.967974000000	2.066331000000
H	-1.752477000000	2.175025000000	0.867760000000
C	-6.703236000000	0.199155000000	-0.344340000000
H	-7.571244000000	0.777435000000	-0.675937000000
H	-6.289592000000	-0.264678000000	-1.248792000000
C	-7.186536000000	-0.926775000000	0.598131000000
H	-6.317161000000	-1.508465000000	0.925232000000
H	-7.590548000000	-0.458289000000	1.507546000000
C	-8.202067000000	-1.830526000000	-0.042504000000
C	-9.030954000000	-1.622671000000	-1.124508000000
N	-8.495044000000	-3.093798000000	0.461324000000
C	-9.838031000000	-2.783448000000	-1.289041000000
H	-9.066928000000	-0.727756000000	-1.729583000000
C	-9.491179000000	-3.685917000000	-0.306731000000
H	-10.595666000000	-2.937159000000	-2.043894000000
C	-7.765576000000	-3.754194000000	1.543051000000
H	-7.536670000000	-3.021824000000	2.321000000000
H	-8.431372000000	-4.496020000000	1.992426000000
C	-6.486528000000	-4.400122000000	1.110570000000
C	-5.170090000000	-4.115856000000	1.328204000000
O	-6.592723000000	-5.493500000000	0.293168000000
C	-4.410596000000	-5.087181000000	0.595989000000
H	-4.779217000000	-3.313100000000	1.936684000000
C	-5.321473000000	-5.893626000000	-0.010027000000
H	-3.335253000000	-5.142448000000	0.522527000000
H	-5.243962000000	-6.752847000000	-0.657661000000
C	-10.016961000000	-5.064635000000	-0.021172000000
H	-9.194944000000	-5.791736000000	-0.049981000000
H	-10.421717000000	-5.110527000000	1.001526000000
C	-11.110802000000	-5.504594000000	-0.999272000000
H	-11.970001000000	-4.827717000000	-0.964133000000

H -10.736421000000 -5.518462000000 -2.027474000000
H -11.462993000000 -6.510866000000 -0.754359000000
C -2.112474000000 2.972420000000 2.844785000000
H -1.023338000000 3.029927000000 2.951323000000
H -2.438761000000 3.975096000000 2.541009000000
C -2.737370000000 2.607780000000 4.195538000000
H -2.489325000000 3.353007000000 4.958874000000
H -3.827204000000 2.543066000000 4.129362000000
H -2.368076000000 1.636987000000 4.546638000000
C 3.878042000000 -5.178098000000 1.087789000000
H 3.935574000000 -4.868624000000 2.138764000000
H 3.573948000000 -6.232189000000 1.101517000000
C 5.261876000000 -5.062617000000 0.471318000000
C 5.475342000000 -5.361298000000 -0.881325000000
C 6.364011000000 -4.678072000000 1.241074000000
C 6.745145000000 -5.295095000000 -1.445247000000
C 7.641433000000 -4.588143000000 0.689384000000
C 7.835144000000 -4.903418000000 -0.657906000000
H 6.889857000000 -5.528343000000 -2.492278000000
H 8.481480000000 -4.290774000000 1.303736000000
N 9.140696000000 -4.827136000000 -1.230493000000
C 10.335892000000 -5.209725000000 -0.583843000000
C 9.434444000000 -4.358498000000 -2.529839000000
C 11.443140000000 -4.955918000000 -1.562573000000
C 10.922173000000 -4.461131000000 -2.686939000000
H 12.474338000000 -5.169079000000 -1.314556000000
H 11.414279000000 -4.159198000000 -3.601805000000
O 10.438364000000 -5.652027000000 0.539980000000
O 8.636894000000 -3.958108000000 -3.349558000000
H 4.634623000000 -5.651199000000 -1.505243000000
H 6.226852000000 -4.440059000000 2.292632000000
H 3.846010000000 -2.490835000000 0.538957000000
H 1.491664000000 -6.051403000000 0.063178000000

R2$_{\textbf{exo-BISM2}}$
C -1.962737000000 1.065210000000 -2.586749000000
C -3.347252000000 1.355695000000 -3.142171000000
C -4.192285000000 0.694002000000 -2.350188000000
C -3.330058000000 0.011110000000 -1.284960000000
O -2.219864000000 0.930126000000 -1.178675000000
H -1.161488000000 1.777319000000 -2.774590000000
H -3.554989000000 1.909162000000 -4.048459000000
H -5.263060000000 0.582533000000 -2.448058000000
C -3.963729000000 -0.309713000000 0.068647000000
H -3.169449000000 -0.531576000000 0.783683000000
H -4.547052000000 -1.226740000000 -0.034784000000
C -1.694471000000 -2.018500000000 -1.133025000000
C -2.606059000000 -1.187994000000 -2.031936000000
C -1.644283000000 -0.424595000000 -2.967468000000
C -0.245765000000 -0.843612000000 -2.542444000000
H -3.320072000000 -1.852966000000 -2.522716000000
H -1.767229000000 -0.616958000000 -4.035235000000
N -0.362977000000 -1.786898000000 -1.504163000000
O -2.049736000000 -2.767715000000 -0.244365000000
O 0.796204000000 -0.438588000000 -3.007638000000
C -0.954615000000 5.119670000000 -2.625766000000

C	-1.975145000000	5.453822000000	-3.468319000000
C	-3.194110000000	5.090890000000	-2.806159000000
C	-2.830113000000	4.556405000000	-1.609385000000
O	-1.469995000000	4.561223000000	-1.484738000000
H	-1.869880000000	5.906901000000	-4.443769000000
H	-4.202956000000	5.213381000000	-3.172397000000
H	-3.381753000000	4.130161000000	-0.785748000000
C	0.535849000000	5.217128000000	-2.735737000000
H	0.779738000000	5.406856000000	-3.785450000000
H	0.986335000000	4.255977000000	-2.476710000000
N	1.169382000000	6.242050000000	-1.907926000000
C	1.925226000000	6.016914000000	-0.758827000000
C	1.179434000000	7.601115000000	-2.210517000000
C	2.396936000000	7.242204000000	-0.337496000000
C	1.930848000000	8.232709000000	-1.242114000000
H	3.020030000000	7.401525000000	0.532580000000
H	2.131871000000	9.293284000000	-1.196217000000
C	0.486604000000	8.163854000000	-3.418426000000
H	0.831033000000	7.647952000000	-4.329323000000
H	-0.593616000000	7.969162000000	-3.362407000000
C	0.708693000000	9.670600000000	-3.604615000000
H	0.353727000000	10.198280000000	-2.711164000000
H	1.784563000000	9.871256000000	-3.674250000000
C	-0.002062000000	10.222067000000	-4.843318000000
H	-1.084869000000	10.062846000000	-4.785104000000
H	0.169098000000	11.297564000000	-4.953271000000
H	0.355102000000	9.734662000000	-5.757879000000
C	2.123587000000	4.680122000000	-0.107404000000
H	2.191230000000	3.867634000000	-0.840563000000
H	3.101337000000	4.701500000000	0.387178000000
C	1.030478000000	4.311463000000	0.937596000000
H	0.930400000000	5.144350000000	1.643708000000
H	0.068320000000	4.234743000000	0.425241000000
C	1.283195000000	3.020904000000	1.657274000000
C	0.793153000000	1.756784000000	1.401076000000
N	2.158174000000	2.909248000000	2.734612000000
C	1.366397000000	0.860754000000	2.349171000000
H	0.091541000000	1.506819000000	0.616340000000
C	2.202797000000	1.589035000000	3.169603000000
H	1.186233000000	-0.202255000000	2.418866000000
C	2.806305000000	4.020236000000	3.407463000000
H	3.781654000000	3.690594000000	3.783239000000
H	3.014570000000	4.809766000000	2.678980000000
C	1.983795000000	4.574740000000	4.534429000000
C	0.781290000000	4.242618000000	5.082837000000
O	2.519604000000	5.650150000000	5.187501000000
C	0.554610000000	5.177401000000	6.149832000000
H	0.140808000000	3.432826000000	4.766847000000
C	1.633250000000	6.003338000000	6.168383000000
H	-0.300594000000	5.222162000000	6.808454000000
H	1.918110000000	6.848294000000	6.775251000000
C	3.046748000000	1.164549000000	4.337747000000
H	4.109298000000	1.361728000000	4.128540000000
H	2.799958000000	1.774888000000	5.217653000000
C	2.881049000000	-0.315301000000	4.696276000000
H	1.842943000000	-0.543378000000	4.956407000000

H	3.164444000000	-0.959768000000	3.858396000000
H	3.510286000000	-0.576208000000	5.552157000000
C	0.763159000000	-2.431163000000	-0.893217000000
C	1.838372000000	-1.678147000000	-0.438509000000
C	0.777289000000	-3.818079000000	-0.765960000000
C	2.957924000000	-2.305426000000	0.120913000000
H	1.812639000000	-0.597557000000	-0.511503000000
C	1.875305000000	-4.471772000000	-0.202380000000
H	-0.082242000000	-4.382281000000	-1.100487000000
C	2.999321000000	-3.714125000000	0.217519000000
N	-4.822164000000	0.739621000000	0.597973000000
C	-6.194275000000	0.842828000000	0.360555000000
C	-4.384232000000	1.869573000000	1.285937000000
C	-6.611695000000	2.041650000000	0.894715000000
C	-5.483351000000	2.684214000000	1.472635000000
H	-7.632503000000	2.400026000000	0.892035000000
H	-5.480164000000	3.629533000000	1.996261000000
C	-2.954736000000	2.062077000000	1.714432000000
H	-2.538976000000	1.109422000000	2.071608000000
H	-2.338591000000	2.331829000000	0.849366000000
C	-7.035803000000	-0.239720000000	-0.250101000000
H	-7.970213000000	0.229289000000	-0.573294000000
H	-6.585683000000	-0.664924000000	-1.156104000000
C	-7.370603000000	-1.401219000000	0.714174000000
H	-6.435743000000	-1.885546000000	1.017689000000
H	-7.793888000000	-0.968786000000	1.632520000000
C	-8.306806000000	-2.411015000000	0.111184000000
C	-9.223204000000	-2.282003000000	-0.910946000000
N	-8.418726000000	-3.709022000000	0.599786000000
C	-9.899910000000	-3.525710000000	-1.054489000000
H	-9.402862000000	-1.386339000000	-1.488709000000
C	-9.387907000000	-4.399263000000	-0.119391000000
H	-10.682508000000	-3.752989000000	-1.763956000000
C	-7.546553000000	-4.302447000000	1.612742000000
H	-7.344170000000	-3.562078000000	2.390244000000
H	-8.093771000000	-5.120381000000	2.089234000000
C	-6.239100000000	-4.797339000000	1.077901000000
C	-4.950291000000	-4.366648000000	1.198433000000
O	-6.283739000000	-5.892867000000	0.257065000000
C	-4.144932000000	-5.243403000000	0.399160000000
H	-4.603941000000	-3.527364000000	1.783875000000
C	-5.002656000000	-6.145773000000	-0.146147000000
H	-3.078420000000	-5.175936000000	0.248357000000
H	-4.878738000000	-6.988954000000	-0.807535000000
C	-9.732675000000	-5.834625000000	0.163167000000
H	-8.837609000000	-6.461828000000	0.060844000000
H	-10.058365000000	-5.946062000000	1.208825000000
C	-10.834292000000	-6.378592000000	-0.751874000000
H	-11.760123000000	-5.805520000000	-0.642145000000
H	-10.532262000000	-6.330131000000	-1.802592000000
H	-11.052665000000	-7.423081000000	-0.511076000000
C	-2.761458000000	3.112707000000	2.820777000000
H	-1.685857000000	3.303305000000	2.907014000000
H	-3.212093000000	4.063330000000	2.508718000000
C	-3.315822000000	2.697026000000	4.187510000000
H	-3.148960000000	3.479522000000	4.935556000000

H	-4.390470000000	2.498782000000	4.141481000000
H	-2.825193000000	1.784412000000	4.546002000000
C	4.203519000000	-4.426243000000	0.839526000000
H	4.149320000000	-4.270092000000	1.921048000000
H	4.080106000000	-5.504604000000	0.708347000000
C	5.614757000000	-4.074643000000	0.367473000000
C	5.972874000000	-4.147871000000	-1.000880000000
C	6.606019000000	-3.751955000000	1.321616000000
C	7.283168000000	-3.853060000000	-1.387309000000
C	7.905503000000	-3.446400000000	0.905474000000
C	8.242968000000	-3.488870000000	-0.444031000000
H	7.563841000000	-3.898183000000	-2.429662000000
H	8.659153000000	-3.195986000000	1.641488000000
N	9.572759000000	-3.176194000000	-0.863114000000
C	10.750992000000	-3.567091000000	-0.193106000000
C	9.906367000000	-2.425711000000	-2.010737000000
C	11.892820000000	-3.014010000000	-0.993373000000
C	11.404195000000	-2.352362000000	-2.044001000000
H	12.920276000000	-3.174182000000	-0.694918000000
H	11.925541000000	-1.824568000000	-2.831410000000
O	10.819947000000	-4.218193000000	0.827207000000
O	9.131671000000	-1.942045000000	-2.807607000000
C	6.328207000000	-3.732205000000	2.811453000000
H	5.559516000000	-3.001450000000	3.088374000000
H	5.989718000000	-4.707902000000	3.177967000000
H	7.235253000000	-3.475184000000	3.363708000000
C	4.079301000000	-1.415592000000	0.610522000000
H	4.465664000000	-1.740762000000	1.580185000000
H	4.926755000000	-1.407292000000	-0.082413000000
H	3.721569000000	-0.389695000000	0.725193000000
C	4.956838000000	-4.567757000000	-2.053863000000
H	4.444987000000	-5.471533000000	-1.699045000000
H	4.173778000000	-3.804471000000	-2.121684000000
C	1.840132000000	-5.988475000000	-0.039979000000
H	2.677943000000	-6.432725000000	-0.594146000000
H	2.027061000000	-6.235420000000	1.013202000000
C	0.553188000000	-6.695871000000	-0.474486000000
H	-0.316354000000	-6.336276000000	0.084986000000
H	0.350776000000	-6.558017000000	-1.541534000000
H	0.641963000000	-7.771154000000	-0.293344000000
C	5.499872000000	-4.839191000000	-3.459996000000
H	5.926848000000	-3.939694000000	-3.914291000000
H	6.271826000000	-5.616020000000	-3.457476000000
H	4.688237000000	-5.178639000000	-4.110508000000

TS2$_{exo\text{-}BISM1}$

C	2.275193000000	0.411684000000	-2.242176000000
C	3.219016000000	1.591886000000	-2.023443000000
C	2.582525000000	2.318901000000	-0.983161000000
C	1.261368000000	1.590107000000	-0.736318000000
O	1.655620000000	0.222210000000	-0.957231000000
H	2.674634000000	-0.533876000000	-2.608280000000
H	3.632140000000	2.077885000000	-2.901115000000
H	2.616484000000	3.399899000000	-0.924029000000
C	0.445162000000	1.825939000000	0.542439000000
H	-0.330783000000	1.060953000000	0.593367000000

H	-0.081580000000	2.772163000000	0.407728000000
C	-1.045466000000	1.234834000000	-1.950990000000
C	0.355020000000	1.833392000000	-2.042295000000
C	1.080162000000	0.962750000000	-3.081108000000
C	0.105063000000	-0.143824000000	-3.445187000000
H	0.274762000000	2.897111000000	-2.274631000000
H	1.407630000000	1.472644000000	-3.989769000000
N	-1.095081000000	0.076449000000	-2.743411000000
O	-1.980139000000	1.669337000000	-1.305377000000
O	0.319928000000	-1.069083000000	-4.196426000000
C	5.019290000000	0.959541000000	-1.179013000000
C	5.603764000000	2.248136000000	-0.963999000000
C	5.008098000000	2.760802000000	0.162557000000
C	4.095672000000	1.771747000000	0.609140000000
O	4.402911000000	0.592705000000	-0.002624000000
H	6.258142000000	2.756769000000	-1.657821000000
H	5.095470000000	3.758062000000	0.571055000000
H	3.600358000000	1.686419000000	1.563610000000
C	5.575896000000	-0.150424000000	-2.044036000000
H	5.975481000000	0.318291000000	-2.950242000000
H	4.767508000000	-0.807142000000	-2.362503000000
N	6.600736000000	-0.997536000000	-1.443080000000
C	6.412104000000	-2.326950000000	-1.060027000000
C	7.943235000000	-0.657802000000	-1.277206000000
C	7.631682000000	-2.805084000000	-0.633561000000
C	8.587607000000	-1.764488000000	-0.767248000000
H	7.813319000000	-3.809951000000	-0.276258000000
H	9.639896000000	-1.822398000000	-0.529469000000
C	8.503175000000	0.687034000000	-1.641883000000
H	8.125049000000	1.001014000000	-2.627513000000
H	8.153973000000	1.453043000000	-0.935549000000
C	10.037449000000	0.721356000000	-1.681918000000
H	10.429053000000	0.446533000000	-0.695253000000
H	10.397727000000	-0.043091000000	-2.380504000000
C	10.584490000000	2.094090000000	-2.082278000000
H	10.262032000000	2.871950000000	-1.380671000000
H	11.678879000000	2.095831000000	-2.097929000000
H	10.238841000000	2.385421000000	-3.080956000000
C	5.109159000000	-3.068551000000	-1.122967000000
H	4.523121000000	-2.799564000000	-2.010369000000
H	5.353906000000	-4.127872000000	-1.258816000000
C	4.201592000000	-2.912935000000	0.130843000000
H	4.794133000000	-3.154470000000	1.021563000000
H	3.917630000000	-1.862414000000	0.234492000000
C	2.948764000000	-3.735179000000	0.074483000000
C	1.672096000000	-3.369475000000	-0.299297000000
N	2.915268000000	-5.096480000000	0.366474000000
C	0.841586000000	-4.523660000000	-0.219008000000
H	1.375678000000	-2.372600000000	-0.595986000000
C	1.622128000000	-5.580692000000	0.198879000000
H	-0.214510000000	-4.574124000000	-0.443065000000
C	4.022811000000	-5.871537000000	0.895240000000
H	3.953211000000	-6.899044000000	0.521410000000
H	4.960786000000	-5.469508000000	0.499255000000
C	4.066896000000	-5.882370000000	2.395803000000
C	3.355261000000	-5.251397000000	3.371322000000

O	5.029886000000	-6.677928000000	2.953573000000
C	3.914997000000	-5.684977000000	4.621248000000
H	2.538498000000	-4.562547000000	3.217121000000
C	4.921526000000	-6.543224000000	4.310869000000
H	3.603418000000	-5.391746000000	5.613292000000
H	5.622610000000	-7.116956000000	4.896306000000
C	1.269945000000	-7.016972000000	0.460750000000
H	1.865258000000	-7.676504000000	-0.189014000000
H	1.545501000000	-7.288313000000	1.489641000000
C	-0.214930000000	-7.323086000000	0.243372000000
H	-0.842327000000	-6.719102000000	0.906016000000
H	-0.514931000000	-7.112319000000	-0.787808000000
H	-0.424402000000	-8.377082000000	0.447149000000
C	-2.225297000000	-0.800680000000	-2.815845000000
C	-2.034299000000	-2.182112000000	-2.741794000000
C	-3.514870000000	-0.278303000000	-2.960386000000
C	-3.136989000000	-3.031925000000	-2.817789000000
H	-1.036269000000	-2.589292000000	-2.636672000000
C	-4.604611000000	-1.140894000000	-3.022789000000
H	-3.662116000000	0.792999000000	-3.009175000000
C	-4.435395000000	-2.530810000000	-2.958072000000
N	1.138043000000	1.870088000000	1.817572000000
C	1.615472000000	3.024438000000	2.436189000000
C	1.429693000000	0.761476000000	2.607899000000
C	2.226484000000	2.633307000000	3.609114000000
C	2.112157000000	1.220145000000	3.717455000000
H	2.692429000000	3.303316000000	4.319327000000
H	2.478795000000	0.604710000000	4.526266000000
C	1.027343000000	-0.633505000000	2.217498000000
H	-0.000430000000	-0.627548000000	1.826454000000
H	1.648773000000	-0.983425000000	1.386108000000
C	1.421935000000	4.419409000000	1.917391000000
H	2.169250000000	5.044486000000	2.415754000000
H	1.635740000000	4.493439000000	0.844532000000
C	0.024627000000	5.022374000000	2.186909000000
H	-0.726826000000	4.444304000000	1.636078000000
H	-0.209231000000	4.875928000000	3.251704000000
C	-0.062701000000	6.476414000000	1.814903000000
C	0.930323000000	7.414808000000	1.628603000000
N	-1.280018000000	7.134054000000	1.669709000000
C	0.307722000000	8.665725000000	1.358212000000
H	1.993539000000	7.232018000000	1.693346000000
C	-1.057148000000	8.475852000000	1.381510000000
H	0.808035000000	9.605588000000	1.174430000000
C	-2.589603000000	6.481961000000	1.669667000000
H	-2.588858000000	5.682572000000	2.414013000000
H	-3.331785000000	7.216621000000	1.994104000000
C	-2.986850000000	5.899943000000	0.348883000000
C	-3.082072000000	4.625885000000	-0.129048000000
O	-3.310136000000	6.790045000000	-0.642576000000
C	-3.484783000000	4.730990000000	-1.501051000000
H	-2.888405000000	3.710986000000	0.410744000000
C	-3.607507000000	6.060158000000	-1.757694000000
H	-3.652597000000	3.915474000000	-2.187969000000
H	-3.885609000000	6.630210000000	-2.630338000000
C	-2.179265000000	9.452480000000	1.166710000000

H	-2.816304000000	9.116122000000	0.338932000000
H	-2.830654000000	9.478008000000	2.053817000000
C	-1.689776000000	10.875260000000	0.878916000000
H	-1.088747000000	11.263569000000	1.706946000000
H	-1.073659000000	10.903944000000	-0.025105000000
H	-2.537672000000	11.550394000000	0.730986000000
C	1.093624000000	-1.661593000000	3.358573000000
H	0.961276000000	-2.652170000000	2.908047000000
H	2.099577000000	-1.657487000000	3.796729000000
C	0.041229000000	-1.458818000000	4.454031000000
H	0.131118000000	-2.222254000000	5.234049000000
H	0.138056000000	-0.478391000000	4.928907000000
H	-0.971229000000	-1.527655000000	4.039491000000
C	-5.628145000000	-3.466929000000	-3.060795000000
H	-6.048392000000	-3.407233000000	-4.072489000000
H	-5.275074000000	-4.498691000000	-2.941885000000
C	-6.735478000000	-3.194641000000	-2.057158000000
C	-8.047238000000	-2.953184000000	-2.476770000000
C	-6.473681000000	-3.194945000000	-0.680066000000
C	-9.074990000000	-2.730522000000	-1.560857000000
C	-7.482425000000	-2.959242000000	0.247937000000
C	-8.791779000000	-2.729743000000	-0.192902000000
H	-10.083288000000	-2.542192000000	-1.906687000000
H	-7.261687000000	-2.966513000000	1.307670000000
N	-9.833105000000	-2.491645000000	0.754097000000
C	-10.875194000000	-1.552186000000	0.598601000000
C	-9.976267000000	-3.158282000000	1.990289000000
C	-11.717459000000	-1.651019000000	1.835337000000
C	-11.199035000000	-2.581544000000	2.639055000000
H	-12.591244000000	-1.028327000000	1.973464000000
H	-11.537951000000	-2.924048000000	3.607632000000
O	-11.048818000000	-0.818373000000	-0.350254000000
O	-9.249775000000	-4.021975000000	2.431784000000
H	-5.461006000000	-3.368933000000	-0.327369000000
H	-8.277985000000	-2.947103000000	-3.538825000000
H	-2.979323000000	-4.105940000000	-2.766369000000
H	-5.604087000000	-0.726799000000	-3.120553000000

TS2$_{exo\text{-}BISM2}$

C	2.580296000000	0.366725000000	-2.255375000000
C	3.660473000000	1.428506000000	-2.104069000000
C	3.205237000000	2.216740000000	-1.040860000000
C	1.823903000000	1.653037000000	-0.689402000000
O	2.029893000000	0.247990000000	-0.930482000000
H	2.840106000000	-0.616851000000	-2.646586000000
H	4.145933000000	1.823164000000	-2.987796000000
H	3.392629000000	3.279793000000	-0.963823000000
C	1.128411000000	1.989383000000	0.637896000000
H	0.288045000000	1.304830000000	0.758962000000
H	0.685709000000	2.979249000000	0.516259000000
C	-0.586875000000	1.564357000000	-1.742875000000
C	0.863201000000	2.004447000000	-1.930745000000
C	1.409418000000	1.068433000000	-3.018101000000
C	0.284503000000	0.101216000000	-3.338144000000
H	0.884392000000	3.072333000000	-2.157519000000
H	1.746511000000	1.547589000000	-3.939674000000

N	-0.832804000000	0.443525000000	-2.553324000000
O	-1.411549000000	2.087839000000	-1.017811000000
O	0.340216000000	-0.817909000000	-4.126027000000
C	5.506026000000	0.522732000000	-1.297634000000
C	6.221430000000	1.736092000000	-1.171810000000
C	5.738851000000	2.360179000000	-0.035852000000
C	4.745117000000	1.510278000000	0.481807000000
O	4.856057000000	0.290542000000	-0.114231000000
H	6.904200000000	2.136615000000	-1.907534000000
H	5.963071000000	3.353811000000	0.325951000000
H	4.272397000000	1.514539000000	1.450410000000
C	5.819339000000	-0.664845000000	-2.173616000000
H	6.185221000000	-0.273980000000	-3.129428000000
H	4.901973000000	-1.211786000000	-2.388346000000
N	6.780177000000	-1.632130000000	-1.648621000000
C	6.463213000000	-2.923316000000	-1.223298000000
C	8.164688000000	-1.466643000000	-1.622263000000
C	7.648536000000	-3.551525000000	-0.909335000000
C	8.711040000000	-2.643516000000	-1.156846000000
H	7.735341000000	-4.568357000000	-0.550229000000
H	9.765799000000	-2.834180000000	-1.021414000000
C	8.850512000000	-0.205713000000	-2.063577000000
H	8.422247000000	0.146550000000	-3.015169000000
H	8.668119000000	0.603002000000	-1.342169000000
C	10.366224000000	-0.364943000000	-2.247665000000
H	10.812674000000	-0.680338000000	-1.297080000000
H	10.560193000000	-1.173279000000	-2.962632000000
C	11.039500000000	0.925303000000	-2.722938000000
H	10.884865000000	1.742200000000	-2.008753000000
H	12.119054000000	0.789162000000	-2.841077000000
H	10.639299000000	1.251185000000	-3.690069000000
C	5.076221000000	-3.488182000000	-1.132636000000
H	4.447239000000	-3.180614000000	-1.977437000000
H	5.170693000000	-4.575181000000	-1.232506000000
C	4.322433000000	-3.161736000000	0.188072000000
H	4.974576000000	-3.415908000000	1.032252000000
H	4.162491000000	-2.082047000000	0.247567000000
C	2.991759000000	-3.841367000000	0.313627000000
C	1.728134000000	-3.364633000000	0.033208000000
N	2.847251000000	-5.161406000000	0.733834000000
C	0.794325000000	-4.405412000000	0.305420000000
H	1.507471000000	-2.369381000000	-0.328275000000
C	1.500627000000	-5.507101000000	0.741542000000
H	-0.279990000000	-4.350651000000	0.202171000000
C	3.919880000000	-6.014769000000	1.212124000000
H	3.700573000000	-7.053347000000	0.940768000000
H	4.843989000000	-5.754323000000	0.686307000000
C	4.131541000000	-5.914326000000	2.694940000000
C	3.603223000000	-5.133751000000	3.678716000000
O	5.063282000000	-6.767965000000	3.218700000000
C	4.251963000000	-5.529828000000	4.897681000000
H	2.850050000000	-4.371380000000	3.548767000000
C	5.122714000000	-6.517499000000	4.562543000000
H	4.086198000000	-5.127433000000	5.886527000000
H	5.822051000000	-7.119512000000	5.121040000000
C	1.030388000000	-6.866879000000	1.172086000000

H	1.473285000000	-7.641256000000	0.527259000000
H	1.393171000000	-7.083824000000	2.186656000000
C	-0.493690000000	-7.015636000000	1.142999000000
H	-0.972405000000	-6.292788000000	1.810731000000
H	-0.887498000000	-6.853726000000	0.134851000000
H	-0.788478000000	-8.018908000000	1.463684000000
C	-2.071185000000	-0.280156000000	-2.575847000000
C	-2.057042000000	-1.672690000000	-2.635248000000
C	-3.284830000000	0.399513000000	-2.565387000000
C	-3.250404000000	-2.398118000000	-2.670248000000
H	-1.108387000000	-2.186417000000	-2.680356000000
C	-4.495403000000	-0.301077000000	-2.577628000000
H	-3.297429000000	1.481520000000	-2.539259000000
C	-4.490869000000	-1.713650000000	-2.602248000000
N	1.906751000000	1.991622000000	1.863830000000
C	2.543160000000	3.104229000000	2.411976000000
C	2.126910000000	0.881208000000	2.674312000000
C	3.182791000000	2.682109000000	3.558626000000
C	2.924386000000	1.293849000000	3.723651000000
H	3.762769000000	3.317091000000	4.214983000000
H	3.274176000000	0.665208000000	4.529760000000
C	1.553628000000	-0.471907000000	2.358277000000
H	0.511393000000	-0.365000000000	2.024519000000
H	2.083656000000	-0.914236000000	1.507664000000
C	2.465640000000	4.497867000000	1.860029000000
H	3.307171000000	5.050260000000	2.289188000000
H	2.613722000000	4.521252000000	0.773919000000
C	1.162342000000	5.257074000000	2.197280000000
H	0.318674000000	4.754228000000	1.709632000000
H	0.982220000000	5.160144000000	3.278054000000
C	1.211458000000	6.703767000000	1.791112000000
C	2.287459000000	7.520413000000	1.514067000000
N	0.067736000000	7.491675000000	1.709977000000
C	1.790703000000	8.828249000000	1.252813000000
H	3.325678000000	7.220252000000	1.512459000000
C	0.418142000000	8.794178000000	1.372425000000
H	2.378633000000	9.702040000000	1.011472000000
C	-1.303190000000	6.991227000000	1.813428000000
H	-1.338049000000	6.205990000000	2.571785000000
H	-1.934212000000	7.808442000000	2.173762000000
C	-1.856479000000	6.441689000000	0.535469000000
C	-2.119316000000	5.180385000000	0.087340000000
O	-2.159040000000	7.351927000000	-0.444410000000
C	-2.610676000000	5.315207000000	-1.252876000000
H	-1.981067000000	4.254480000000	0.625367000000
C	-2.613443000000	6.647561000000	-1.522129000000
H	-2.914606000000	4.516644000000	-1.912568000000
H	-2.895542000000	7.235738000000	-2.381360000000
C	-0.599996000000	9.887780000000	1.209248000000
H	-1.326263000000	9.612326000000	0.434223000000
H	-1.181711000000	10.001119000000	2.136969000000
C	0.024568000000	11.241119000000	0.855558000000
H	0.720736000000	11.572437000000	1.632243000000
H	0.576450000000	11.185042000000	-0.087821000000
H	-0.750678000000	12.005355000000	0.748029000000
C	1.575233000000	-1.464990000000	3.531625000000

H	1.308511000000	-2.448133000000	3.126852000000
H	2.600172000000	-1.558384000000	3.912374000000
C	0.619009000000	-1.111312000000	4.675938000000
H	0.671080000000	-1.855082000000	5.478091000000
H	0.850921000000	-0.133333000000	5.107091000000
H	-0.417899000000	-1.079690000000	4.321687000000
C	-5.799380000000	-2.508471000000	-2.639384000000
H	-6.212317000000	-2.409752000000	-3.647828000000
H	-5.564879000000	-3.571185000000	-2.536277000000
C	-6.900973000000	-2.191883000000	-1.627828000000
C	-8.214369000000	-1.936772000000	-2.083172000000
C	-6.649214000000	-2.230443000000	-0.234502000000
C	-9.235742000000	-1.680036000000	-1.163502000000
C	-7.683289000000	-1.945200000000	0.661456000000
C	-8.969846000000	-1.666784000000	0.202529000000
H	-10.238544000000	-1.477333000000	-1.518658000000
H	-7.499431000000	-1.965556000000	1.725971000000
N	-10.016159000000	-1.383809000000	1.133434000000
C	-11.000416000000	-0.386670000000	0.968193000000
C	-10.222357000000	-2.056733000000	2.356028000000
C	-11.874946000000	-0.454091000000	2.185066000000
C	-11.425595000000	-1.421065000000	2.987303000000
H	-12.716180000000	0.214007000000	2.312982000000
H	-11.803770000000	-1.756106000000	3.943924000000
O	-11.111913000000	0.368099000000	0.026241000000
O	-9.555412000000	-2.965496000000	2.802118000000
C	-8.586581000000	-1.956232000000	-3.552185000000
H	-8.043920000000	-1.202345000000	-4.133598000000
H	-8.380813000000	-2.927834000000	-4.015510000000
H	-9.653099000000	-1.754130000000	-3.676840000000
C	-5.770726000000	0.513082000000	-2.589331000000
H	-6.452968000000	0.189243000000	-3.381166000000
H	-6.319556000000	0.437839000000	-1.646393000000
H	-5.544887000000	1.568699000000	-2.761236000000
C	-5.271792000000	-2.596640000000	0.300043000000
H	-4.944042000000	-3.516398000000	-0.202016000000
H	-4.549277000000	-1.832475000000	-0.006790000000
C	-3.191405000000	-3.917193000000	-2.804070000000
H	-3.641614000000	-4.379794000000	-1.914725000000
H	-3.832554000000	-4.218798000000	-3.642197000000
C	-5.158551000000	-2.806074000000	1.812961000000
H	-5.358595000000	-1.884777000000	2.369591000000
H	-5.851255000000	-3.573408000000	2.173042000000
H	-4.143147000000	-3.124301000000	2.067358000000
C	-1.805919000000	-4.533645000000	-3.023232000000
H	-1.320051000000	-4.127559000000	-3.915918000000
H	-1.135566000000	-4.370537000000	-2.173824000000
H	-1.902106000000	-5.615205000000	-3.159293000000

$P2_{exo\text{-}BISM1}$

C	2.297183000000	-0.185263000000	-2.258982000000
C	3.752269000000	0.279551000000	-2.006801000000
C	3.539136000000	1.203082000000	-0.749587000000
C	1.990730000000	1.216109000000	-0.619688000000
O	1.650911000000	-0.139790000000	-0.973228000000
H	2.146260000000	-1.166974000000	-2.705936000000

H	4.155684000000	0.796559000000	-2.881027000000
H	3.933269000000	2.214318000000	-0.870481000000
C	1.310995000000	1.683985000000	0.684986000000
H	0.359423000000	1.152419000000	0.758267000000
H	1.055069000000	2.736263000000	0.563944000000
C	-0.072266000000	2.285554000000	-1.825668000000
C	1.427599000000	2.005858000000	-1.879230000000
C	1.588648000000	0.968041000000	-3.004870000000
C	0.174028000000	0.598461000000	-3.425706000000
H	1.953327000000	2.952654000000	-2.022900000000
H	2.140518000000	1.308707000000	-3.884465000000
N	-0.722694000000	1.421453000000	-2.721708000000
O	-0.628547000000	3.115562000000	-1.135907000000
O	-0.134016000000	-0.253853000000	-4.228560000000
C	4.769395000000	-0.791583000000	-1.451443000000
C	6.058017000000	-0.001042000000	-1.189516000000
C	5.831175000000	0.708349000000	-0.083170000000
C	4.394267000000	0.393445000000	0.310444000000
O	4.259483000000	-0.969902000000	-0.127211000000
H	6.909692000000	0.040206000000	-1.854631000000
H	6.455502000000	1.461899000000	0.380893000000
H	4.122675000000	0.485296000000	1.355825000000
C	4.864253000000	-2.090308000000	-2.263979000000
H	5.423717000000	-1.863049000000	-3.179045000000
H	3.864872000000	-2.384692000000	-2.581939000000
N	5.456993000000	-3.248104000000	-1.610862000000
C	4.727714000000	-4.336864000000	-1.125615000000
C	6.820062000000	-3.523508000000	-1.489534000000
C	5.634483000000	-5.270236000000	-0.674664000000
C	6.939649000000	-4.763372000000	-0.899021000000
H	5.376047000000	-6.229857000000	-0.247122000000
H	7.871318000000	-5.260793000000	-0.671275000000
C	7.907527000000	-2.606797000000	-1.973407000000
H	7.605441000000	-2.122815000000	-2.914375000000
H	8.072195000000	-1.789070000000	-1.258218000000
C	9.248422000000	-3.318148000000	-2.210970000000
H	9.589415000000	-3.768695000000	-1.271455000000
H	9.097992000000	-4.146696000000	-2.912953000000
C	10.329496000000	-2.371578000000	-2.739700000000
H	10.517017000000	-1.549376000000	-2.039527000000
H	11.276988000000	-2.897568000000	-2.893385000000
H	10.037621000000	-1.928633000000	-3.699114000000
C	3.232527000000	-4.464171000000	-1.153534000000
H	2.794300000000	-3.970123000000	-2.028743000000
H	3.007662000000	-5.527961000000	-1.291275000000
C	2.493000000000	-3.949050000000	0.113560000000
H	2.925269000000	-4.438461000000	0.994874000000
H	2.700740000000	-2.882241000000	0.227215000000
C	1.007201000000	-4.144505000000	0.047955000000
C	0.019990000000	-3.249020000000	-0.307795000000
N	0.386463000000	-5.373988000000	0.260667000000
C	-1.225803000000	-3.938577000000	-0.298730000000
H	0.189428000000	-2.209935000000	-0.554738000000
C	-0.984764000000	-5.248150000000	0.058437000000
H	-2.195062000000	-3.521889000000	-0.533215000000
C	1.034022000000	-6.564050000000	0.780228000000

H	0.572333000000	-7.449215000000	0.326621000000
H	2.080632000000	-6.572731000000	0.462653000000
C	0.965094000000	-6.660880000000	2.277225000000
C	0.334696000000	-5.940526000000	3.247193000000
O	1.662324000000	-7.695795000000	2.837029000000
C	0.665143000000	-6.570529000000	4.495208000000
H	-0.288576000000	-5.073238000000	3.088870000000
C	1.467671000000	-7.623390000000	4.189524000000
H	0.345756000000	-6.271277000000	5.482956000000
H	1.964686000000	-8.380175000000	4.775740000000
C	-1.923903000000	-6.409727000000	0.218961000000
H	-1.645156000000	-7.217453000000	-0.475047000000
H	-1.824613000000	-6.838238000000	1.226118000000
C	-3.389693000000	-6.037310000000	-0.023736000000
H	-3.721131000000	-5.265030000000	0.677130000000
H	-3.537631000000	-5.654843000000	-1.038346000000
H	-4.034940000000	-6.911182000000	0.105162000000
C	-2.143821000000	1.374715000000	-2.891793000000
C	-2.800512000000	0.139792000000	-2.921573000000
C	-2.870360000000	2.558749000000	-3.029145000000
C	-4.180376000000	0.100066000000	-3.092745000000
H	-2.233356000000	-0.777326000000	-2.821724000000
C	-4.254203000000	2.499765000000	-3.192275000000
H	-2.361824000000	3.514361000000	-2.997226000000
C	-4.930699000000	1.276085000000	-3.231708000000
N	2.030712000000	1.541086000000	1.936483000000
C	2.897820000000	2.496614000000	2.465276000000
C	2.028571000000	0.410319000000	2.747125000000
C	3.449269000000	1.955120000000	3.606785000000
C	2.906965000000	0.652279000000	3.784426000000
H	4.156270000000	2.457264000000	4.253687000000
H	3.130144000000	-0.032505000000	4.589506000000
C	1.195742000000	-0.798526000000	2.424874000000
H	0.156984000000	-0.494739000000	2.225110000000
H	1.548748000000	-1.250221000000	1.491116000000
C	3.096478000000	3.870170000000	1.893997000000
H	4.048359000000	4.246050000000	2.282469000000
H	3.206008000000	3.848365000000	0.802274000000
C	1.988126000000	4.881821000000	2.259567000000
H	1.032023000000	4.538988000000	1.842677000000
H	1.861532000000	4.859992000000	3.351945000000
C	2.291975000000	6.274371000000	1.784854000000
C	3.422846000000	6.797340000000	1.194439000000
N	1.387588000000	7.319898000000	1.936913000000
C	3.201481000000	8.186769000000	0.976994000000
H	4.321182000000	6.247216000000	0.952900000000
C	1.939002000000	8.494013000000	1.436700000000
H	3.897681000000	8.887074000000	0.538422000000
C	0.023610000000	7.177153000000	2.445024000000
H	0.021443000000	6.430509000000	3.242639000000
H	-0.269128000000	8.128205000000	2.898681000000
C	-0.983512000000	6.768631000000	1.416085000000
C	-1.648387000000	5.605043000000	1.164304000000
O	-1.338670000000	7.712624000000	0.488027000000
C	-2.461173000000	5.836489000000	0.006556000000
H	-1.567817000000	4.686477000000	1.726919000000

C	-2.234082000000	7.125561000000	-0.359832000000
H	-3.108350000000	5.125635000000	-0.485102000000
H	-2.606548000000	7.752475000000	-1.154795000000
C	1.202751000000	9.804014000000	1.460203000000
H	0.249849000000	9.707772000000	0.924398000000
H	0.937577000000	10.068895000000	2.495383000000
C	2.008363000000	10.956447000000	0.852334000000
H	2.945858000000	11.114672000000	1.394316000000
H	2.256039000000	10.753886000000	-0.194226000000
H	1.435240000000	11.887448000000	0.889754000000
C	1.180664000000	-1.870041000000	3.526524000000
H	0.765863000000	-2.785168000000	3.090981000000
H	2.211842000000	-2.113473000000	3.810223000000
C	0.368540000000	-1.485606000000	4.768343000000
H	0.747931000000	-0.570943000000	5.233112000000
H	-0.682396000000	-1.312709000000	4.508803000000
H	0.397218000000	-2.281844000000	5.519776000000
C	-6.434535000000	1.219777000000	-3.442482000000
H	-6.645376000000	0.767208000000	-4.419297000000
H	-6.820318000000	2.245328000000	-3.495933000000
C	-7.195233000000	0.450586000000	-2.375804000000
C	-8.017112000000	-0.629309000000	-2.712445000000
C	-7.106822000000	0.815383000000	-1.025343000000
C	-8.728847000000	-1.332549000000	-1.741102000000
C	-7.823387000000	0.137855000000	-0.044522000000
C	-8.636549000000	-0.945028000000	-0.401985000000
H	-9.361441000000	-2.164256000000	-2.023361000000
H	-7.741765000000	0.433809000000	0.993473000000
N	-9.369408000000	-1.649942000000	0.599732000000
C	-8.892017000000	-1.957934000000	1.892487000000
C	-10.682736000000	-2.145798000000	0.449181000000
C	-9.997805000000	-2.698879000000	2.582862000000
C	-11.034220000000	-2.805244000000	1.749134000000
H	-9.888591000000	-3.056376000000	3.598037000000
H	-11.998920000000	-3.270169000000	1.902478000000
O	-11.379032000000	-2.054531000000	-0.538609000000
O	-7.804871000000	-1.673611000000	2.346077000000
H	-6.467479000000	1.644346000000	-0.734852000000
H	-8.104369000000	-0.933278000000	-3.752242000000
H	-4.814075000000	3.425091000000	-3.300262000000
H	-4.684693000000	-0.861787000000	-3.108792000000

P2$_{exo\text{-}BISM2}$

C	2.580360000000	-0.287795000000	-2.236290000000
C	4.078432000000	0.083517000000	-2.133338000000
C	4.051038000000	1.014203000000	-0.863991000000
C	2.527102000000	1.114237000000	-0.570098000000
O	2.074461000000	-0.217371000000	-0.890228000000
H	2.323332000000	-1.253108000000	-2.670324000000
H	4.423486000000	0.577117000000	-3.045160000000
H	4.487050000000	2.001632000000	-1.029426000000
C	2.019555000000	1.608965000000	0.802063000000
H	1.058233000000	1.122756000000	0.982124000000
H	1.800624000000	2.672218000000	0.707737000000
C	0.395567000000	2.274676000000	-1.546484000000
C	1.872623000000	1.941898000000	-1.757557000000

C	1.875199000000	0.915576000000	-2.904383000000
C	0.412824000000	0.628890000000	-3.205001000000
H	2.416362000000	2.870697000000	-1.945900000000
H	2.368012000000	1.236832000000	-3.825119000000
N	-0.375263000000	1.463552000000	-2.394627000000
O	-0.054742000000	3.104239000000	-0.782355000000
O	-0.003877000000	-0.179980000000	-4.004547000000
C	5.077384000000	-1.051519000000	-1.681266000000
C	6.433708000000	-0.344288000000	-1.561954000000
C	6.368887000000	0.379207000000	-0.443408000000
C	4.965325000000	0.153781000000	0.102797000000
O	4.700894000000	-1.198496000000	-0.309541000000
H	7.211429000000	-0.357166000000	-2.313404000000
H	7.084165000000	1.092615000000	-0.053024000000
H	4.814560000000	0.261331000000	1.170756000000
C	5.000590000000	-2.352141000000	-2.492786000000
H	5.482741000000	-2.163198000000	-3.459146000000
H	3.956734000000	-2.573782000000	-2.710614000000
N	5.569423000000	-3.551578000000	-1.896134000000
C	4.814573000000	-4.590148000000	-1.344442000000
C	6.914235000000	-3.924845000000	-1.910434000000
C	5.691563000000	-5.590550000000	-0.988112000000
C	7.001641000000	-5.176057000000	-1.338603000000
H	5.408087000000	-6.532758000000	-0.538257000000
H	7.912953000000	-5.740752000000	-1.204756000000
C	8.011289000000	-3.081005000000	-2.494474000000
H	7.659199000000	-2.580274000000	-3.409088000000
H	8.289315000000	-2.274113000000	-1.802461000000
C	9.278096000000	-3.877216000000	-2.842651000000
H	9.670032000000	-4.347390000000	-1.933076000000
H	9.014619000000	-4.695353000000	-3.523218000000
C	10.367171000000	-3.002732000000	-3.469908000000
H	10.666722000000	-2.193118000000	-2.794484000000
H	11.262318000000	-3.588553000000	-3.701334000000
H	10.021757000000	-2.543557000000	-4.403558000000
C	3.319196000000	-4.606551000000	-1.214475000000
H	2.828094000000	-4.065374000000	-2.031710000000
H	3.001190000000	-5.648170000000	-1.337406000000
C	2.764724000000	-4.060270000000	0.131536000000
H	3.260131000000	-4.590401000000	0.954161000000
H	3.058954000000	-3.012110000000	0.226382000000
C	1.271398000000	-4.151059000000	0.242259000000
C	0.314285000000	-3.181526000000	0.025621000000
N	0.595363000000	-5.336825000000	0.524600000000
C	-0.966774000000	-3.780612000000	0.193629000000
H	0.525598000000	-2.153562000000	-0.235015000000
C	-0.777404000000	-5.110545000000	0.504974000000
H	-1.925513000000	-3.290041000000	0.102222000000
C	1.216485000000	-6.579075000000	0.945180000000
H	0.637091000000	-7.421013000000	0.547890000000
H	2.209870000000	-6.656054000000	0.494095000000
C	1.334631000000	-6.697487000000	2.437533000000
C	0.891525000000	-5.949994000000	3.487327000000
O	2.018729000000	-7.790664000000	2.893037000000
C	1.332720000000	-6.624706000000	4.676332000000
H	0.319990000000	-5.036548000000	3.418773000000

C	2.007220000000	-7.728166000000	4.259917000000
H	1.167542000000	-6.320429000000	5.699721000000
H	2.517598000000	-8.529841000000	4.770037000000
C	-1.769573000000	-6.205203000000	0.777602000000
H	-1.644493000000	-7.016427000000	0.044060000000
H	-1.568941000000	-6.659800000000	1.757842000000
C	-3.223609000000	-5.725341000000	0.737154000000
H	-3.403669000000	-4.948031000000	1.485956000000
H	-3.476272000000	-5.310655000000	-0.243534000000
H	-3.907780000000	-6.554473000000	0.939595000000
C	-1.808489000000	1.472895000000	-2.421918000000
C	-2.506768000000	0.268057000000	-2.438744000000
C	-2.499674000000	2.679519000000	-2.455860000000
C	-3.903992000000	0.250953000000	-2.476504000000
H	-1.952276000000	-0.659313000000	-2.449700000000
C	-3.897693000000	2.692632000000	-2.472762000000
H	-1.952306000000	3.614290000000	-2.461407000000
C	-4.616950000000	1.474973000000	-2.456537000000
N	2.865873000000	1.430129000000	1.967066000000
C	3.835030000000	2.339082000000	2.390040000000
C	2.892310000000	0.303687000000	2.783160000000
C	4.478293000000	1.772125000000	3.469510000000
C	3.890199000000	0.500991000000	3.716680000000
H	5.277708000000	2.237292000000	4.030811000000
H	4.163694000000	-0.192628000000	4.498333000000
C	1.968474000000	-0.860647000000	2.561886000000
H	0.930738000000	-0.504584000000	2.475490000000
H	2.193566000000	-1.329557000000	1.597560000000
C	4.045270000000	3.697981000000	1.788516000000
H	5.054301000000	4.019571000000	2.065468000000
H	4.030596000000	3.668068000000	0.691648000000
C	3.044015000000	4.772052000000	2.267126000000
H	2.030355000000	4.485904000000	1.957324000000
H	3.035063000000	4.755726000000	3.366870000000
C	3.375941000000	6.145506000000	1.756654000000
C	4.467007000000	6.603142000000	1.048766000000
N	2.555831000000	7.241875000000	2.000578000000
C	4.306212000000	8.003996000000	0.852361000000
H	5.301068000000	6.002123000000	0.715488000000
C	3.119584000000	8.383356000000	1.441932000000
H	4.992480000000	8.663705000000	0.341199000000
C	1.245184000000	7.176402000000	2.646598000000
H	1.280668000000	6.423314000000	3.437227000000
H	1.058289000000	8.138641000000	3.131429000000
C	0.114481000000	6.839716000000	1.725590000000
C	-0.625960000000	5.713116000000	1.520824000000
O	-0.293430000000	7.822661000000	0.861546000000
C	-1.545306000000	6.010394000000	0.462202000000
H	-0.528633000000	4.776461000000	2.049756000000
C	-1.299857000000	7.297936000000	0.102078000000
H	-2.273908000000	5.341506000000	0.029176000000
H	-1.726126000000	7.961856000000	-0.633622000000
C	2.468879000000	9.734515000000	1.540428000000
H	1.461145000000	9.697661000000	1.107560000000
H	2.329508000000	10.009365000000	2.597364000000
C	3.273644000000	10.840176000000	0.850169000000

H	4.270418000000	10.939903000000	1.290816000000
H	3.398052000000	10.628524000000	-0.216321000000
H	2.763931000000	11.803308000000	0.946166000000
C	2.020428000000	-1.930240000000	3.664219000000
H	1.515190000000	-2.823219000000	3.281379000000
H	3.062975000000	-2.225056000000	3.835137000000
C	1.368744000000	-1.505764000000	4.985048000000
H	1.841290000000	-0.611119000000	5.400819000000
H	0.305625000000	-1.280727000000	4.840517000000
H	1.439620000000	-2.302362000000	5.733303000000
C	-6.146004000000	1.509188000000	-2.501886000000
H	-6.451006000000	1.203956000000	-3.507100000000
H	-6.470273000000	2.550088000000	-2.416753000000
C	-6.945509000000	0.700683000000	-1.478385000000
C	-7.983911000000	-0.162129000000	-1.901701000000
C	-6.709759000000	0.855826000000	-0.091961000000
C	-8.721413000000	-0.882817000000	-0.955529000000
C	-7.473968000000	0.137828000000	0.831765000000
C	-8.469612000000	-0.739601000000	0.406096000000
H	-9.510937000000	-1.545627000000	-1.288102000000
H	-7.281743000000	0.259363000000	1.890660000000
N	-9.232722000000	-1.477479000000	1.361170000000
C	-8.710619000000	-2.083786000000	2.523826000000
C	-10.622211000000	-1.710639000000	1.287185000000
C	-9.870995000000	-2.738384000000	3.212831000000
C	-10.976864000000	-2.521224000000	2.498337000000
H	-9.743030000000	-3.282254000000	4.139225000000
H	-11.994093000000	-2.837457000000	2.687084000000
O	-11.372674000000	-1.330236000000	0.414475000000
O	-7.554303000000	-2.071519000000	2.886448000000
C	-5.664958000000	1.805182000000	0.451593000000
H	-4.647518000000	1.470006000000	0.231086000000
H	-5.769504000000	2.808767000000	0.026334000000
H	-5.760277000000	1.895823000000	1.536513000000
C	-4.582603000000	4.043306000000	-2.537090000000
H	-5.218390000000	4.141089000000	-3.424138000000
H	-5.217847000000	4.235079000000	-1.664745000000
H	-3.839729000000	4.843874000000	-2.579992000000
C	-4.630846000000	-1.084429000000	-2.557510000000
H	-5.155796000000	-1.269866000000	-1.613339000000
H	-5.422795000000	-0.998871000000	-3.310918000000
C	-3.771954000000	-2.306575000000	-2.899746000000
H	-3.218218000000	-2.165710000000	-3.833524000000
H	-3.046465000000	-2.540552000000	-2.114319000000
H	-4.413124000000	-3.185361000000	-3.018420000000
C	-8.392129000000	-0.332247000000	-3.356359000000
H	-7.515834000000	-0.407242000000	-4.009426000000
H	-8.917983000000	-1.287775000000	-3.457140000000
C	-9.313681000000	0.791666000000	-3.863565000000
H	-9.613084000000	0.610943000000	-4.901230000000
H	-10.219769000000	0.852128000000	-3.253046000000
H	-8.820473000000	1.767908000000	-3.820877000000

References

1. Lima, G.M.R.; Orozco, F.; Picchioni, F.; Moreno-Villoslada, I.; Pucci, A.; Bose, R.K.; Araya-Hermosilla, R. Electrically Self-Healing Thermoset MWCNTs Composites Based on Diels-Alder and Hydrogen Bonds. *Polymers (Basel)* **2019**, *11*, 1885, doi:10.3390/polym11111885.
